# CD38‐NADase is a new major contributor to Duchenne muscular dystrophic phenotype

**DOI:** 10.15252/emmm.202012860

**Published:** 2022-03-17

**Authors:** Antoine de Zélicourt, Abdallah Fayssoil, Mbarka Dakouane‐Giudicelli, Isley De Jesus, Ahmed Karoui, Faouzi Zarrouki, Florence Lefebvre, Arnaud Mansart, Jean‐Marie Launay, Jerome Piquereau, Mariana G Tarragó, Marcel Bonay, Anne Forand, Sophie Moog, France Piétri‐Rouxel, Elise Brisebard, Claudia C S Chini, Sonu Kashyap, Matthew J Fogarty, Gary C Sieck, Mathias Mericskay, Eduardo N Chini, Ana Maria Gomez, José‐Manuel Cancela, Sabine de la Porte

**Affiliations:** ^1^ Université Paris‐Saclay UVSQ Inserm END‐ICAP Versailles France; ^2^ Institut des Neurosciences Paris‐Saclay, CNRS, Université Paris‐Saclay Saclay France; ^3^ Signalisation et Physiopathologie Cardiovasculaire INSERM UMR‐S 1180 ‐ Université Paris‐Saclay Châtenay‐Malabry France; ^4^ Université Paris‐Saclay UVSQ Inserm 2I Versailles France; ^5^ Service de Biochimie INSERM UMR S942 Hôpital Lariboisière Paris France; ^6^ Department of Anesthesiology and Kogod Aging Center Mayo Clinic Rochester Minnesota USA; ^7^ Centre de Recherche en Myologie Faculté de Médecine de la Pitié Salpêtrière Sorbonne Université‐UMRS974‐Inserm‐Institut de Myologie Paris France; ^8^ Inovarion Paris France; ^9^ INRAE Oniris UMR0703 APEX Nantes France

**Keywords:** calcium, cardiomyopathy, CD38, DMD, NAD^+^, Musculoskeletal System

## Abstract

Duchenne muscular dystrophy (DMD) is characterized by progressive muscle degeneration. Two important deleterious features are a Ca^2+^ dysregulation linked to Ca^2+^ influxes associated with ryanodine receptor hyperactivation, and a muscular nicotinamide adenine dinucleotide (NAD^+^) deficit. Here, we identified that deletion in *mdx* mice of CD38, a NAD^+^ glycohydrolase‐producing modulators of Ca^2+^ signaling, led to a fully restored heart function and structure, with skeletal muscle performance improvements, associated with a reduction in inflammation and senescence markers. Muscle NAD^+^ levels were also fully restored, while the levels of the two main products of CD38, nicotinamide and ADP‐ribose, were reduced, in heart, diaphragm, and limb. In cardiomyocytes from *mdx/CD38* ^−/−^ mice, the pathological spontaneous Ca^2+^ activity was reduced, as well as in myotubes from DMD patients treated with isatuximab (SARCLISA^®^) a monoclonal anti‐CD38 antibody. Finally, treatment of *mdx* and utrophin–dystrophin‐deficient (*mdx/utr* ^−/−^ *)* mice with CD38 inhibitors resulted in improved skeletal muscle performances. Thus, we demonstrate that CD38 actively contributes to DMD physiopathology. We propose that a selective anti‐CD38 therapeutic intervention could be highly relevant to develop for DMD patients.

## Graphical abstract

Duchenne muscular dystrophy (DMD) is characterized by muscle NAD^+^ deficit and muscle Ca^2+^ overload. In various dystrophic mouse models and a human DMD cell culture, we have targeted the multifunctional enzyme CD38, a NAD^+^ glycohydrolase that generates Ca^2+^ signaling modulators from NAD^+^, by genetic or pharmacological inhibition.

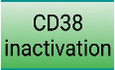

**The paper explained**

**Problem**

Duchenne muscular dystrophy (DMD) originates from the loss of the subsarcolemmal protein dystrophin. The disease is manifested by a dramatic degeneration of patient’s skeletal, smooth, and cardiac muscles. Since dilated cardiomyopathy and heart failure are now the main causes of patient death, cardiac muscle preservation is of high interest in DMD. However, today no treatments are available, and patient’s death occurred at around 30 years old.

The absence of dystrophin leads to a fragile plasma membrane and to the activation of numerous Ca^2+^ channels, resulting in a Ca^2+^ homeostasis dysregulation, which is now considered as a major feature of the DMD pathogenesis and responsible for the muscle cell death. This massive Ca^2+^ homeostasis dysregulation is also associated with a severe deficit in muscle NAD^+^ levels, a molecule required in numerous cell processes and necessary for cell survival. An important recognized strategy in DMD is to prevent the occurrence of the deleterious Ca^2+^ homeostasis dysregulation by targeting the cellular players regulating the Ca^2+^ signaling in muscle. More recently, another strategy under investigation consists in restoring muscle NAD^+^ levels. However, despite considerable effort, no efficient target has been found so far.

**Results**

Here, we found that the enzyme CD38 (which produces modulators of Ca^2+^ channels from NAD^+^) is expressed in muscle tissues and highly contributes to NAD^+^ deficit and Ca^2+^ dysregulation observed in DMD. To investigate the role of CD38 in the DMD phenotype, we used a mouse model (*mdx*) of DMD where CD38 has been knocked out. We found that CD38 deletion in *mdx* mice led to fully restored heart function and cardiac muscle NAD^+^ levels. Limb and diaphragm were also functionally improved with a reduction in the muscular fibrosis/regeneration cycles and inflammation markers, associated with restored NAD^+^ levels, suggesting that CD38 deletion prevented cell damage and necrosis in *mdx* mice. The pathological Ca^2+^ channel activity was also prevented in *mdx* mice deleted for CD38 and in human DMD‐cultured muscles treated with a monoclonal anti‐CD38 antibody. Finally, treatment of *mdx* and *mdx/utr* ^−/−^ mice with CD38 inhibitors resulted in improved skeletal muscle performance.

**Impact**

Our paper could have a great clinical impact since treatments available have a very limited effect on the onset of the disease, which remains very costly for individuals and for caregivers. Currently, in the absence of any curative treatment, only palliative and comfort treatments are available for DMD patients. A pharmacological therapy to treat DMD has several advantages: It could be applicable in the short term, has much less risk of immune rejection, and is less prone to tumorigenicity.

In view of the important role of Ca^2+^ dysregulation in the pathogenesis of DMD, targeting important Ca^2+^ regulators could turn to be very effective. Furthermore, restoring the lower NAD^+^ muscle levels is also an important therapeutic goal. Here, we demonstrated that CD38 participates in DMD physiopathology and that its inhibition can restore simultaneously these two parameters. Since an anti‐CD38 therapy, such as isatuximab, is approved for the treatment of multiple myeloma, this study proposes that CD38 could be a possible therapeutic target in DMD, especially for one of its main features, namely the dilated cardiomyopathy.

## Introduction

Duchenne muscular dystrophy (DMD), a common disease among the rare diseases, affects only males with an incidence evaluated between 1 in 4,500 and 1 in 6,500 newborns (Mendell & Lloyd‐Puryear, 2013; Ryder *et al*, 2017). DMD originates from the loss of function of a gene carried by the X chromosome, encoding dystrophin, a protein of the subsarcolemmal cytoskeleton complex (Hoffman *et al*, 1987). The disease is manifested by a dramatic degeneration of patient’s skeletal, smooth, and cardiac muscles, and in the absence of a curative treatment, only palliative treatments are available and considerable multidisciplinary care is required to extend the patient’s median life expectancy to around 30 years (Birnkrant *et al*, 2018a,b,c; Landfeldt *et al*, 2020).

The absence of dystrophin leads to a fragile plasma membrane and to the activation of numerous Ca^2+^ channels, resulting in a Ca^2+^ homeostasis dysregulation, which is considered as a major feature in DMD (Allen *et al*, 2016; Espinosa *et al*, 2016). Importantly, numerous studies established that Ca^2+^ influx through plasma membrane Ca^2+^ channels, associated with Ca^2+^ release through oversensitive ryanodine receptors (RyRs), is central to the development of muscle weakness, cardiomyopathy, and myocyte cell death (Bellinger *et al*, 2009; Fauconnier *et al*, 2010; Sarma *et al*, 2010; Prosser *et al*, 2011; Ather *et al*, 2013). Intriguingly, another major deleterious consequence of the lack of dystrophin has recently been reported in DMD: a cellular NAD^+^ deficit, which leads to mitochondrial activity impairments and muscle degeneration (Chalkiadaki *et al*, 2014; Ryu *et al*, 2016; Zhang *et al*, 2016). However, it is still unclear what are the mechanisms involved in these processes, and the possible contribution of a major signaling enzyme in Ca^2+^ and NAD^+^ metabolism, namely CD38, has never been explored. CD38 is a 45‐kDa single‐chain transmembrane enzyme, a member of the ADP‐ribosyl cyclase family. In muscle and cardiomyocytes, CD38 is expressed as both a type II and a type III transmembrane protein, with its catalytic site exposed to the extracellular space and to the cytosol, respectively (Zhao *et al*, 2012; Lin *et al*, 2017; Liu *et al*, 2017). Through its NAD^+^ glycohydrolase activity (Lee, 2006; Partida‐Sanchez *et al*, 2007; Camacho‐Pereira *et al*, 2016; Fliegert *et al*, 2017a), CD38 is now considered to be a major NAD^+^ consumer in mammals, including in heart and skeletal muscles (Aksoy *et al*, 2006; Camacho‐Pereira *et al*, 2016; Wang *et al*, 2018). Importantly, from NAD^+^, CD38 produces positive modulators not only of RyRs but also of a plasma membrane Ca^2+^ channel, the transient receptor potential cation channel subtype melastatin 2 (TRPM2) present in cardiac and skeletal muscles (Rakovic *et al*, 1999; Higashida *et al*, 2000; Mojzisová *et al*, 2001; Patel *et al*, 2001; Hohenegger *et al*, 2002; Cancela *et al*, 2003; Lee, 2006; Cosker *et al*, 2010; Miller *et al*, 2013; Nebel *et al*, 2013; Chen *et al*, 2020; Fliegert *et al*, 2020). Last but not least, studies in wild‐type (WT) mice have shown that isoproterenol and angiotensin II pathways require CD38 activity, particularly in the heart (Kim *et al*, 2010; Nebel *et al*, 2013; Gul *et al*, 2016; Guan *et al*, 2017; Lin *et al*, 2017). This is important since both are involved in the DMD cardiomyopathy (Fayssoil *et al*, 2017), and although all muscles are severely altered in DMD, the dilated cardiomyopathy and heart failure are the main causes of DMD patient death. Thus, cardiac muscle preservation is of high interest in this genetic disease (Nigro *et al*, 1990; Kieny *et al*, 2013).

For all these reasons, we hypothesized that CD38 has a key role in many aspects of the DMD pathophysiology and that its inhibition could lead to a potential therapeutic approach for this devastating disease.

## Results

### Restoration of NAD^+^ levels in *mdx/CD38^−/−^* mice

**Figure 1:**
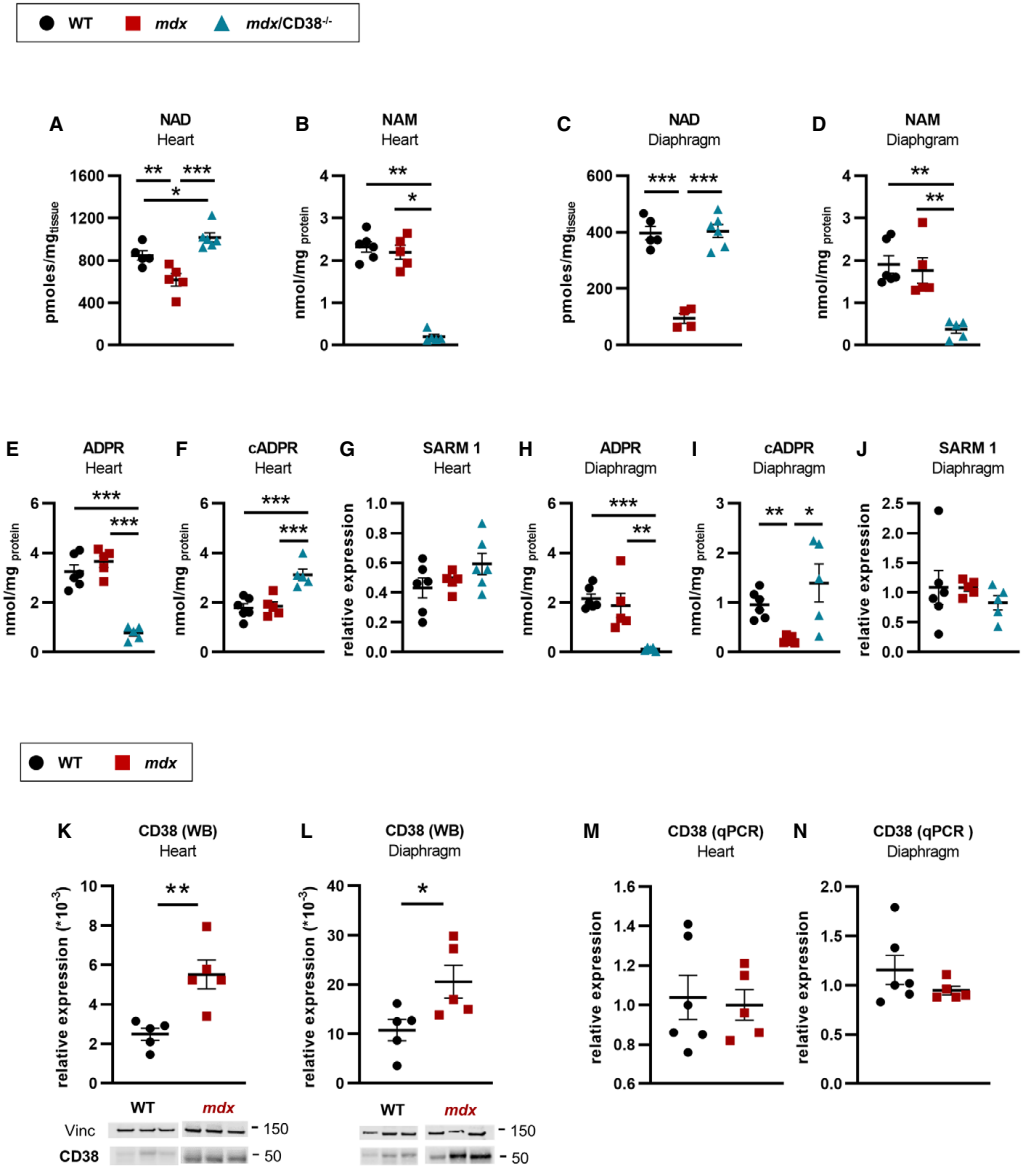
Restoration of NAD^+^ levels in *mdx/CD38* ^−/−^ mice A, BNAD and nicotinamide (NAM) levels in the heart of WT (*n* = 5 and *n* = 6, respectively), *mdx* (*n* = 5 and *n* = 5, respectively), and *mdx*/*CD38* ^−/−^ (*n* = 6 and *n* = 5, respectively) mice.C, DNAD and NAM levels in diaphragm of WT (*n* = 5 and *n* = 6, respectively), *mdx* (*n* = 4 and *n* = 5, respectively), and *mdx*/*CD38* ^−/−^ (*n* = 6 and *n* = 5, respectively) mice.E–GLevels of ADP‐ribose (ADPR) (E), cyclic ADP‐ribose (cADPR) (F) expressed as nmol/mg protein, and (G) qPCR analysis of mRNA levels of sterile alpha and Toll/interleukin‐1 receptor motif‐containing 1 (SARM1) in the heart of WT (*n* = 6), *mdx* (*n* = 5) and *mdx*/*CD38* ^−/−^ (*n* = 5, *n* = 5, and *n* = 6, respectively) mice.H–JLevels of ADPR (H), cADPR (I) expressed as nmol/mg protein, and (J) qPCR analysis of mRNA levels of SARM1 in the diaphragm of WT (*n* = 6), *mdx* (*n* = 5) and *mdx*/*CD38* ^−/−^ (*n* = 5) mice.K, LWestern blot analysis of CD38 protein expression in heart (K) and diaphragm (L) of WT (*n* = 5 and *n* = 6, respectively) and *mdx* (*n* = 5) mice. Vinculin is used as housekeeping protein control, and the dot plots show the ratio of CD38 to vinculin.M, NqPCR analysis of CD38 mRNA levels in the heart (M) and diaphragm (N) of WT (*n* = 6) and *mdx* (*n* = 5) mice. Data information: A–N: Each dot of the graphs represents a mouse and is measured in duplicate except for K,L, a value/mouse. After normality and variance comparison tests, significance was assessed using: A,C,E,F,G,H: ANOVA followed by Fisher's LSD test; B: the Kruskal–Wallis test followed by Dunn's test; D: the Kruskal–Wallis followed by the Mann–Whitney tests; I: Welch’s ANOVA followed by Welch’s *t*‐tests; J: ANOVA; K,L,M: unpaired Student’s *t*‐test; and N: unpaired Welch’s *t*‐test. Values are expressed as means ± SEM. Significance: *P < 0.05, **P < 0.01, and ***P < 0.001. Source data are available online for this figure.

To investigate the role of CD38 in DMD, we generated *mdx/CD38* ^−/−^ mice. CD38 being an NAD^+^ consumer and since it has been reported that CD38 expression increases with age in WT mice (Camacho‐Pereira *et al*, 2016), we first evaluated the impact of CD38 deletion on cardiac and skeletal muscles NAD^+^ levels in aged (20 months old) *mdx* mice. In *mdx* mice, as previously published (Chalkiadaki *et al*, 2014; Ryu *et al*, 2016; Zhang *et al*, 2016), heart (Fig 1A) and diaphragm (Fig 1C) showed a dramatic deficit (45 and 80% reduction, respectively) in NAD^+^ levels. Importantly, we found that the NAD^+^ levels were fully restored to normal values in *mdx/CD38* ^−/−^ mice due to the reduced consumption of NAD^+^ in the absence of CD38 both in the heart (Fig 1A) and in the diaphragm (Fig 1C), whereas nicotinamide (NAM), the product of NAD^+^ consumption by CD38, was reduced (Fig 1B and D). CD38 is a multifunctional enzyme catalyzing the synthesis of cyclic ADP‐ribose (cADPR) from NAD^+^ cyclization and mostly producing ADP‐ribose (ADPR) from NAD^+^ hydrolysis. In the absence of CD38 (*mdx/CD38* ^−/−^ mice), ADPR was downregulated, whereas cADPR was surprisingly upregulated in heart (Fig 1E and F) and diaphragm (Fig 1H and I). The increase in cADPR production could be explained by sterile alpha and Toll/interleukin‐1 receptor motif‐containing 1 (SARM1), an enzyme with ADP‐ribosyl cyclase activity, which is also expressed in the heart (Fig 1G) and diaphragm (Fig 1J) and known to be activated by nicotinamide mononucleotide (NMN) (Essuman *et al*, 2017; Zhao *et al*, 2019) that we found to be increased in *mdx/CD38* ^−/−^ mice (Appendix Fig S1A).

In addition, the NAD^+^ metabolized by CD38 was increased in heart and diaphragm of *mdx* compared with WT mice (Appendix Fig S1B). The subcellular distribution of NAD^+^ in the cytosol, nucleus, and mitochondria indicated that CD38 consumed mostly the cytosolic NAD^+^ (Appendix Fig S1C). Finally, we looked at the nicotinamide phosphoribosyltransferase (NAMPT) mRNA levels and found a reduced expression both in *mdx* and in *mdx/CD38* ^−/−^, suggesting that NAD^+^ synthesis could be impaired in those mice (Appendix Fig S1D). In contrast, we found that one of the main NAD^+^ consumer enzymes, the poly‐ADP‐ribose polymerase 1 (PARP 1) mRNA level was reduced in *mdx* mice (heart and diaphragm) and restored to normal value in *mdx/CD38* ^−/−^ heart (Appendix Fig S1D). In CD38^−/−^ mice, the sole effect we found was an increase in the NAD^+^ levels in the heart and diaphragm, with no effect on the main enzymes involved in NAD^+^ homeostasis (Appendix Fig S2).

In parallel, we found a twofold to threefold increase in CD38 expression in the heart (Fig 1K) and diaphragm (Fig 1L) of old *mdx* mice compared with WT mice, with no change detected in mRNA levels (Fig 1M and N). In contrast, no difference in CD38 protein expression was observed in young (3‐month‐old) *mdx* mice compared with WT mice (Appendix Fig S1E), whereas the level of CD38 mRNA was increased (Appendix Fig S1F). The discrepancies between protein and mRNA levels could be related to the CD38 protein turnover, which might be higher in young *mdx* mice than in the older ones.

### Restoration of the cardiac function and structure in *mdx/CD38^−/−^* mice

**Figure 2:**
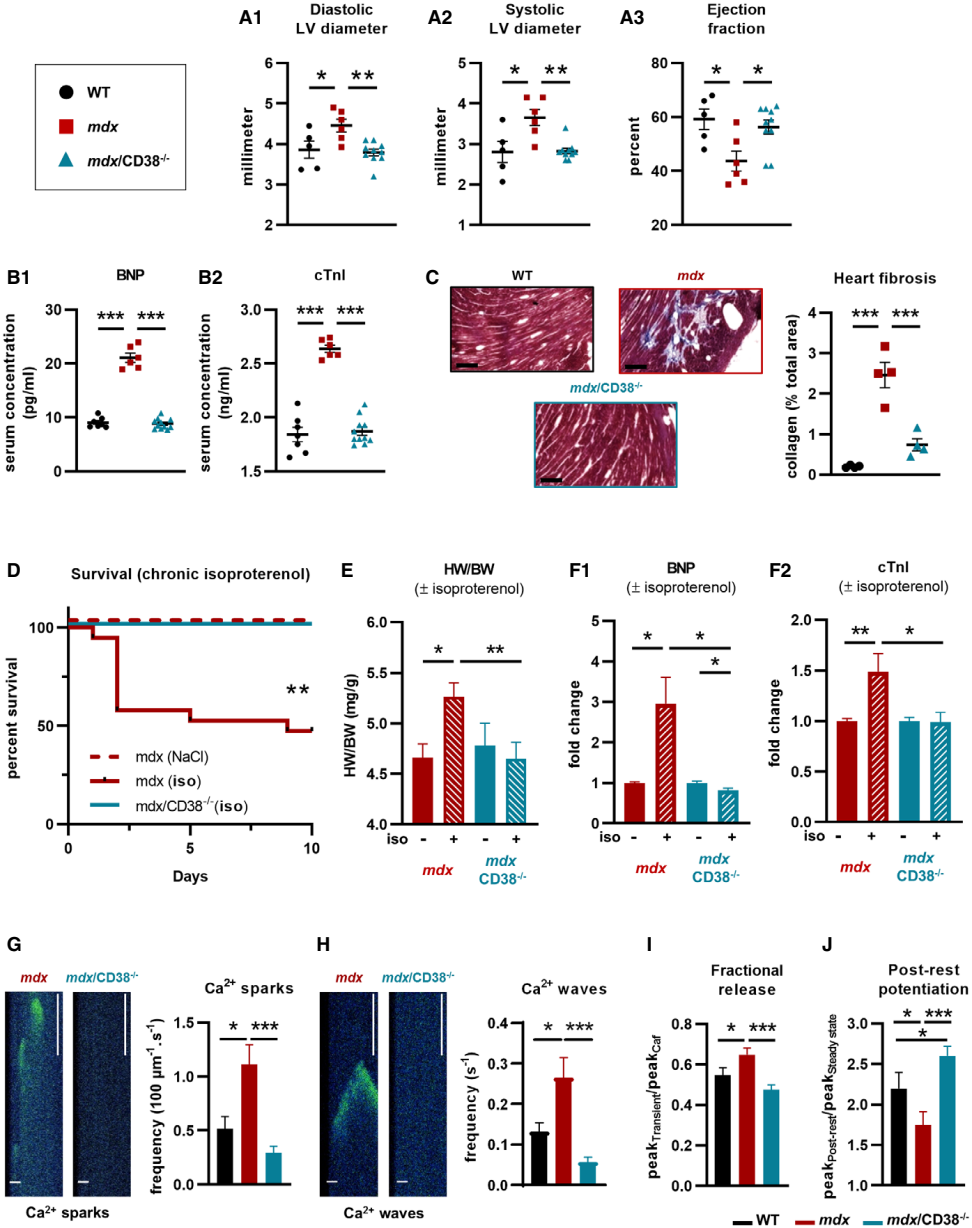
Deletion of CD38 prevented heart dysfunction and reduces spontaneous Ca^2+^ activity in isolated cardiomyocytes of *mdx/CD38* ^−/−^ mice ACardiac function evaluated by echocardiography in *mdx*/*CD38* ^−/−^ mice: The dot plots show the left ventricular (LV) diastolic (A_1_) and systolic (A_2_) inner diameters and LV ejection (A_3_) fraction in 7‐month‐old WT (*n* = 5), *mdx* (*n* = 6), and *mdx/*CD38^−/−^ (*n* = 10) mice.BPlasma levels of cardiac stress biomarkers: brain natriuretic peptide (BNP) (B_1_) and cardiac troponin I (cTnI) (B_2_) in 7‐month‐old WT (*n* = 7), *mdx* (*n* = 6), and *mdx/CD38* ^−/−^ (*n* = 11) mice.CCropped images revealed the collagen (blue) stained by Masson’s trichrome staining in the heart of WT, *mdx*, and *mdx/CD38* ^−/−^ mice. Dot plot showing the quantification of heart collagen staining area (% total area) in 7‐month‐old WT, *mdx*, and *mdx/CD38* ^−/−^ mice (*n* = 4 per group). Scale bars: 200 µm.DIsoproterenol‐induced heart failure in 3‐month‐old mice. The Kaplan–Meier curve shows the survival rate of *mdx* mice (NaCl, *n* = 5), and *mdx* (*n* = 19) and *mdx*/*CD38* ^−/−^ (*n* = 9) mice following isoproterenol (iso, subcutaneous injection) at 2.5 mg/kg/d for 10 days.EHistogram showing isoproterenol‐induced heart hypertrophy in *mdx* NaCl (*n* = 5) mice, and surviving *mdx* (isoproterenol, *n* = 9), *mdx/CD38* ^−/−^ NaCl (*n* = 9), and *mdx/CD38* ^−/−^ (isoproterenol, *n* = 9) mice, expressed as heart weight/body weight ratio (HW/BW).FPlasma levels of cardiac stress biomarkers: brain natriuretic peptide (BNP) (F_1_) and cardiac troponin I (cTnI) (F_2_) following isoproterenol treatment in *mdx* NaCl (*n* = 5), and surviving *mdx* (isoproterenol, *n* = 8), *mdx/CD38* ^−/−^ NaCl (*n* = 5), and *mdx/CD38* ^−/−^ (isoproterenol, *n* = 8) mice.G, HTime‐lapse images recorded by confocal microscopy in “line‐scanning mode”, showing Ca^2+^ sparks and waves in cardiomyocytes at rest, extracted from *mdx* and *mdx/CD38* ^−/−^ mice; scale bars: 10 µM (horizontal), 500 ms (vertical). Bar graphs showing the averaged Ca^2+^ spark (G) and wave (H) frequencies in cardiomyocytes isolated from WT (*n* = 21 cells), *mdx* (*n* = 28 cells), and *mdx/CD38* ^−/−^ (*n* = 32 cells) mice.IBar graph showing the fractional release following caffeine application in cardiomyocytes from WT (*n* = 24 cells), *mdx* (*n* = 33 cells), and *mdx/CD38* ^−/−^ (*n* = 32 cells) mice.JBar graph showing the post‐rest potentiation in cardiomyocytes from WT (*n* = 24 cells), *mdx* (*n* = 28 cells), and *mdx/CD38* ^−/−^ (*n* = 32 cells) mice. Data information: A,B,C: Each dot of the graphs represents a mouse. A,F in duplicate; C in triplicate; and D,E one value/mouse. G,H,I,J: experiments performed in three mice of each group. After normality and variance comparison tests, significance was assessed using: A_1_,B_1_,B_2_,C,I: ANOVA followed by Fisher's LSD test; A_2_,A_3_,G,H,J: the Kruskal–Wallis test followed by Dunn’s test; D: the log‐rank Mantel–Cox test and the log‐rank test for trend and the Gehan–Breslow–Wilcoxon test; E,F_2_: the Kruskal–Wallis test followed by the Mann–Whitney tests; and F_1_: ANOVA followed by Student’s/ Welch’s *t*‐tests. Values are expressed as means ± SEM. Significance: *P < 0.05, **P < 0.01, and ***P < 0.001. Source data are available online for this figure.

DMD patients older than 18 years suffer from cardiac dysfunctions. Similarly, *mdx* mice show a progressive development of cardiac defects from 6 months of age (Quinlan *et al*, 2004; Spurney *et al*, 2008; Au *et al*, 2011). Since cardiomyopathy is a typical feature of DMD, we next focused on the effect of CD38 deletion in *mdx* heart dysfunction, knowing that CD38 deletion in WT mice had no impact on cardiac function and structure (Appendix Fig S3A–C). Echocardiography in 7‐month‐old *mdx* mice (Fig 2A) revealed a significant increase in the diastolic (15%) and systolic (30%) left ventricular inner diameters (respectively LVDD and LVSD), and a 27% reduction in the left ventricular ejection fraction (LVEF) compared with WT mice. We then looked at *mdx/CD38* ^−/−^ mice and found a full protection of the cardiac function (Fig 2A), indicating that CD38 activity could contribute to the *mdx* cardiomyopathy development.

Associated with the full protection of the cardiac function, brain natriuretic peptide (BNP) and cardiac troponin I (cTnI) serum levels, two well‐known biomarkers of cardiac stress, were both increased in *mdx* mice (Fig 2B) and restored to normal values in *mdx/CD38* ^−/−^ mice (Fig 2B). DMD heart in *mdx* mice is also characterized by progressive collagen infiltration due to heart necrosis. Accordingly, we found that *mdx* hearts (8–11 months old) showed an increased (2.46%) collagen infiltration (Fig 2C), evaluated by Masson's trichrome staining, which was reduced to 0.74% in the *mdx/CD38* ^−/−^ hearts (Fig 2C).

To further investigate the role of CD38 in the development of the cardiomyopathy in *mdx* mice, we administrated isoproterenol (2.5 mg/kg/d subcutaneously for 10 days) to young *mdx* mice. Even in the absence of an established cardiac dysfunction, 3‐month‐old *mdx* mice appear particularly sensitive to chronic isoproterenol infusion (Fig 2D): 10 of 19 died within 10 days, and the surviving *mdx* mice displayed cardiac hypertrophy as shown by the 21% increase in cardiac mass index (Fig 2E). The deletion of CD38 fully prevented isoproterenol‐induced death (100% *mdx/CD38* ^−/−^ mice survived; Fig 2D) and cardiac hypertrophy (Fig 2E). Isoproterenol‐treated *mdx* mice also showed an important increase in BNP and cTnI plasma levels, which was also prevented in *mdx/CD38* ^−/−^ mice (Fig 2F). Altogether, we found that CD38 deletion in *mdx* mice protected the heart from isoproterenol‐induced stress, a protection equally observed in WT mice with deletion of CD38 (Appendix Fig S3D–F).

### Pathological Ca^2+^ activity in cardiomyocytes is dramatically reduced in *mdx/CD38^−/−^* mice

DMD heart dysfunction has been attributed to early impairments of cardiomyocyte Ca^2+^ homeostasis. Indeed, a feature of *mdx* cardiomyocytes is the pathological increase in the diastolic spontaneous Ca^2+^ activity (Fauconnier *et al*, 2010; Sarma *et al*, 2010; Ather *et al*, 2013; Lorin *et al*, 2013) resulting from the opening of a cluster of RyRs leading to Ca^2+^ sparks (Fig 2G), which may propagate to neighboring clusters leading to arrhythmogenic Ca^2+^ waves (Cheng *et al*, 1993, 1996) (Fig 2H). The frequencies of Ca^2+^ sparks and Ca^2+^ waves were twofold higher in 3‐month‐old *mdx* cardiomyocytes and fully restored to WT values in *mdx/CD38* ^−/−^ cardiomyocytes (Fig 2G and H). To further investigate the RyR function, we electrically stimulated the cardiac myocytes at 2 Hz and then emptied the SR by rapid caffeine application. With this maneuver, we calculated an index of fractional release, which estimates the amount of Ca^2+^ released through RYR during a twitch by a given amount of Ca^2+^ stored in the SR. Figure 2I shows that the fractional release was increased (+20%) in *mdx* cardiomyocytes compared with WT and fully restored to normal levels in *mdx/CD38* ^−/−^ cardiomyocytes (Fig 2I). During rest, the SR accumulates Ca^2+^ so that the first electrical stimulation after a rest period is higher than that at steady state during regular electrical stimulation. The proportion of increase in the amplitude of the first [Ca^2+^]_i_ transient compared with the amplitude of the [Ca^2+^]_i_ transient at steady state is named post‐rest potentiation. *Mdx* cardiomyocytes also showed a lower (−20%) post‐rest potentiation value (Fig 2J), indicating a reduced capacity of the *mdx* cardiomyocytes to increase the amount of Ca^2+^ stored in the SR during rest (Sabourin *et al*, 2018), which was restored in *mdx/CD38* ^−/−^ cardiomyocytes (Fig 2I and J), supporting the higher SR Ca^2+^ leak of the RyR2 in *mdx* mice, as evaluated by the higher Ca^2+^ spark and wave occurrence. These data clearly demonstrate that CD38, known as a producer of modulators that could act directly or indirectly on RyR (Mojzisová *et al*, 2001; Patel *et al*, 2001; Hohenegger *et al*, 2002; Cancela *et al*, 2003; Lee, 2006; Zhang *et al*, 2009; Cosker *et al*, 2010; Fliegert *et al*, 2018), not only is a key contributor to the excessive Ca^2+^ activity but also contributes to the oversensitization of RyRs in *mdx* cardiomyocytes (Fig 2, Appendix Fig S3G–I).

### Improvement in respiratory function and diaphragm structure in *mdx/CD38^−/−^* mice

**Figure 3:**
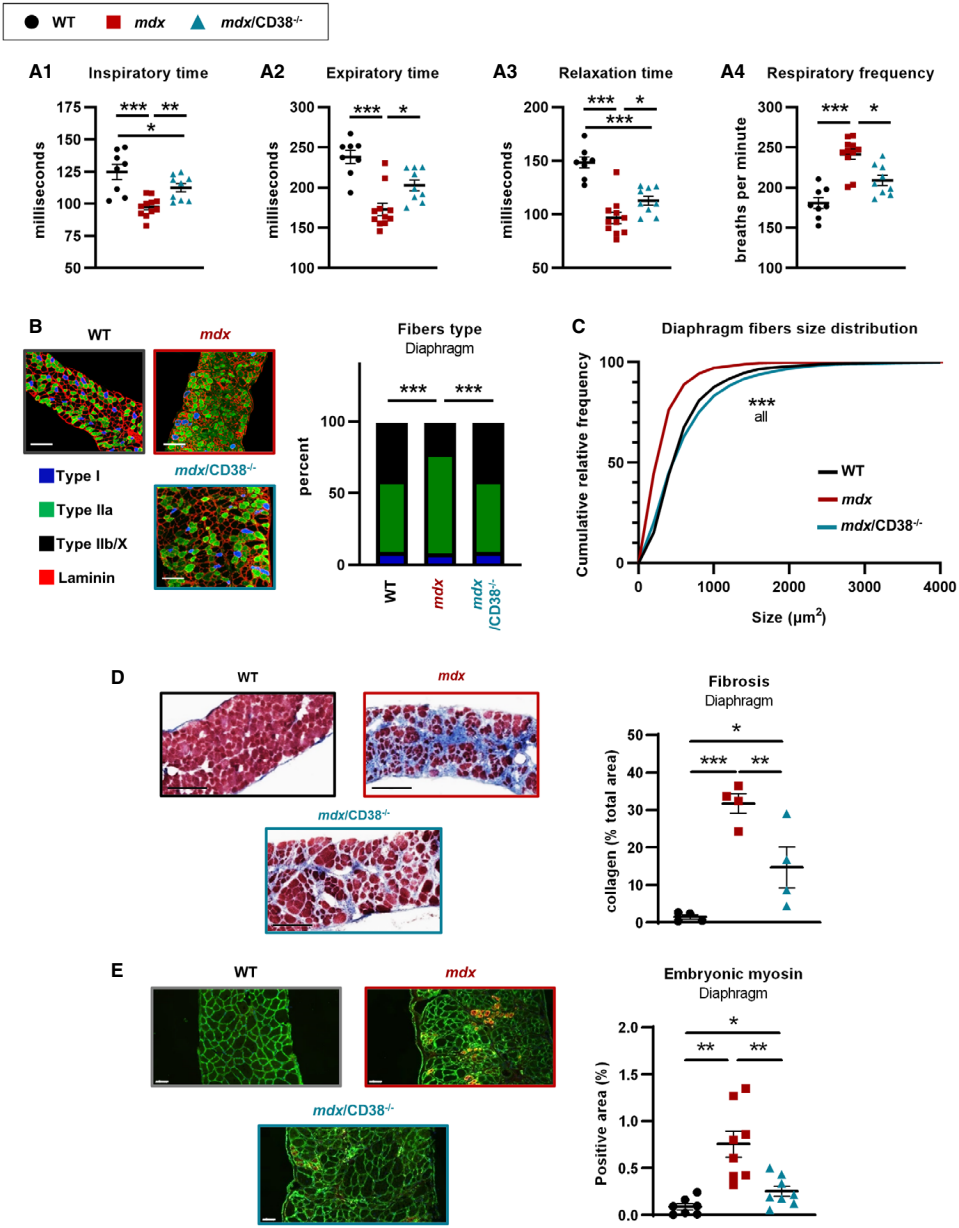
Deletion of CD38 improved diaphragm structure and function in *mdx/CD38* ^−/−^ mice Measurement of the ventilatory mechanic by barometric plethysmography: dot plots showing inspiratory (A_1_) and expiratory times (A_2_), the relaxation time (A_3_), and the respiratory frequency (A_4_) in WT (*n* = 8), *mdx* (*n* = 11), and *mdx/CD38* ^−/−^ (*n* = 9) mice.Images showing muscle fiber typology revealed by immunostaining. Localization of the slow MyHC (Type I) fiber and fast MyHC (type IIa and IIb/X) fibers, along with laminin (red) on transverse cross sections from diaphragm of WT, *mdx*, and *mdx/CD38* ^−/−^ mice. Scale bars: 100 µm. Histogram showing the percentage of I, IIa, and IIb/X fiber‐type distribution in diaphragm of WT (*n* = 7), *mdx* (*n* = 6), and *mdx/CD38* ^−/−^ (*n* = 7) mice.Fiber size distribution in the diaphragm of WT (*n* = 7), *mdx* (*n* = 6), and *mdx/CD38* ^−/−^ (*n* = 7) mice.Images revealing the collagen (blue) by Masson’s trichrome staining in the diaphragm of WT, *mdx*, and *mdx/CD38* ^−/−^ mice. Quantification of collagen staining area (% total area) in the diaphragm of WT, *mdx*, and *mdx/CD38* ^−/−^ mice (*n* = 4 per group). Scale bars: 200 µm.Embryonic myosin expression: immunostaining showing its localization along with laminin (green) on transverse cross sections from diaphragm of WT, *mdx*, and *mdx/CD38* ^−/−^ mice. Scale bars: 50 µm. Histogram showing the relative proportion of embryonic myosin area (% total area) in the diaphragm of WT (*n* = 7), *mdx* (*n* = 8), and *mdx/CD38* ^−/−^ (*n* = 8) mice. Data information: Each dot of the graphs (A,D,E) represents a mouse. A,B,C one value/mouse; D in duplicate; and E one value/mouse. After normality and variance comparison tests, significance was assessed using: A_1_,A_3_,D: ANOVA followed by Fisher's LSD test; A_2_, A_4_: the Kruskal–Wallis test followed by Dunn’s test; B: the chi‐square test; C: the Kolmogorov–Smirnov test; and E: Welch’s ANOVA followed by Welch’s *t*‐tests. Values are expressed as means ± SEM. Significance: *P < 0.05, **P < 0.01, and ***P < 0.001. WT images in 3B and 3E have been redisplayed in S4B and S4E, respectively. Source data are available online for this figure.

Respiratory function, a major parameter affected in patients with DMD, was evaluated in *mdx/CD38* ^−/−^ mice. In 20‐month‐old mice, inspiratory, expiratory, and relaxation times were reduced, and *in fine* respiratory frequency was increased in *mdx* mice compared with WT mice and partially restored in *mdx/CD38* ^−/−^ mice (Fig 3A). This last result prompted us to look at the diaphragm fiber type and size distribution. Mature mammals’ myofibers are classified, according to the myosin heavy chain (MyHC) they express, as slow‐contracting aerobic (type I) and fast‐contracting anaerobic (type IIa, IIb, and IIx) fibers. Muscle fiber‐type expression remains plastic under specific conditions such as in respiratory diseases (Polla *et al*, 2004). To identify the effects of the deletion of CD38 on muscle fibers patterning phenotype, we quantified the number of myofibers of each type, in sections of diaphragms from WT, *CD38* ^−/−^ (Appendix Fig S4B), *mdx*, and *mdx/CD38* ^−/−^ adult mice (Fig 3B). Compared with WT, *mdx* diaphragm has more type IIa fibers and a reduced number of type I fibers, which was fully reverted in *mdx*/CD38^−/−^ diaphragm to the WT mouse profile (Fig 3B). Then, fiber size distribution (cross‐sectional area) was quantified since it has been reported that, due to the repeated cycles of necrosis and regeneration, *mdx* mice display a smaller fiber size distribution (Briguet *et al*, 2004; Dumonceaux *et al*, 2010; Pertl *et al*, 2013). Indeed, we found that the mean fiber size distribution was different between WT and *mdx* mice, with a higher proportion of small diameter fibers in *mdx* mice, which was restored to a WT profile in *mdx/CD38* ^−/−^ mice (Fig 3C). Collectively, these data indicate that CD38 deletion prevented the alterations in muscle fiber type and size distribution observed in *mdx* mouse diaphragm.

Diaphragm collagen deposition, a hallmark of tissue damage highly displayed in *mdx* diaphragm, was visualized using Masson’s trichrome staining. As shown in Fig 3D, *mdx* mouse diaphragm displayed a high level of interstitial collagen deposition (stained blue), which was clearly reduced in *mdx/CD38* ^−/−^ mice (Fig 3D). Finally, we performed an immunostaining of positive embryonic myosin fibers as index of the activation of satellite cells due to the cycles of degeneration/regeneration of *mdx* muscles. Totally absent in WT mice, the regeneration process observed in *mdx* mice was strongly reduced in the diaphragm of *mdx/CD38* ^−/−^ mice (Fig 3E). This also reflects the protective effect of CD38 deletion in *mdx* mice.

### Inflammation and senescence markers are reduced in *mdx/CD38^−/−^* diaphragm

**Figure 4:**
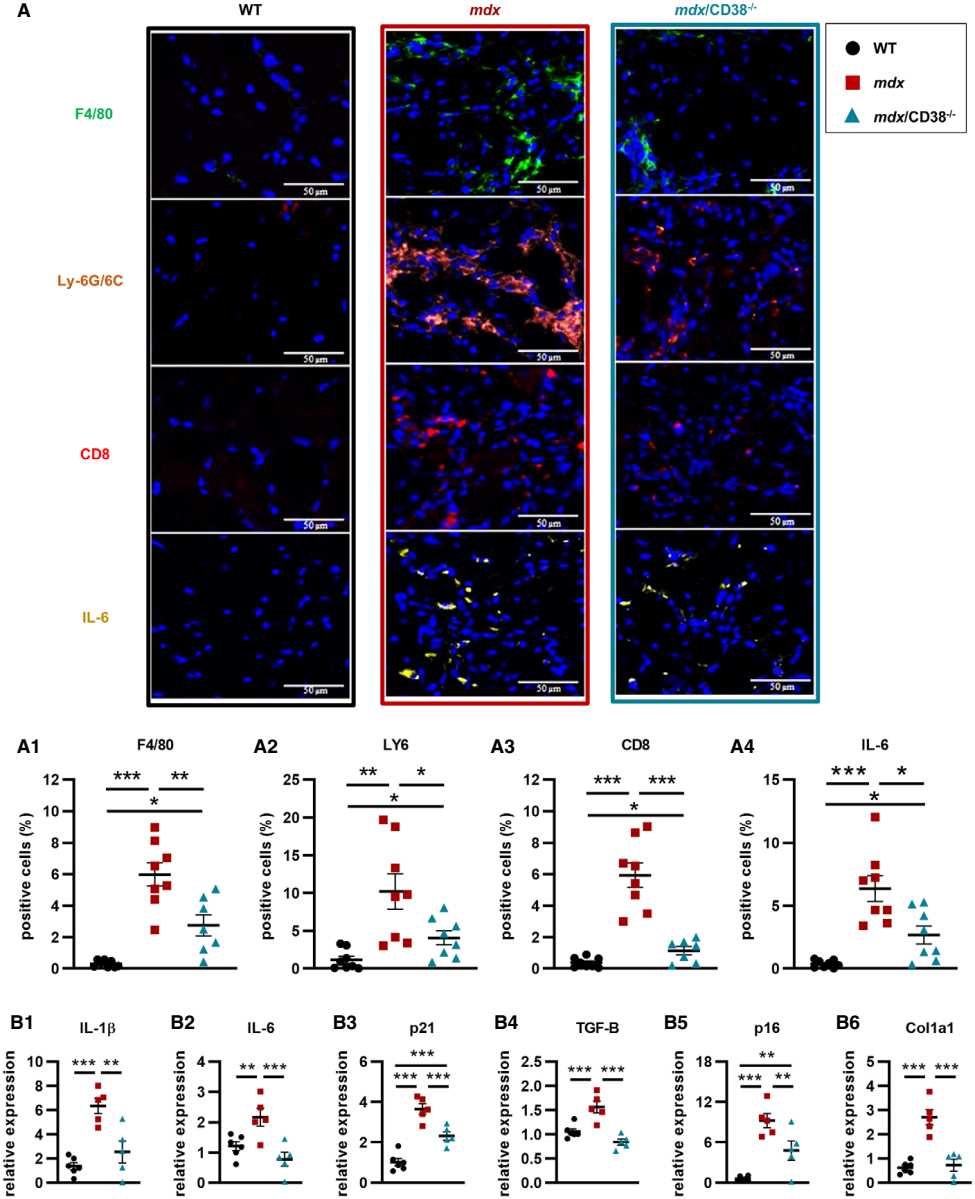
Deletion of CD38 reduced cell infiltrations and inflammation markers in diaphragm in *mdx/CD38* ^−/−^ mice Images showing immunostaining of myeloid cells showing F4/80 (macrophage marker), Ly‐6G/6C (monocyte, granulocyte, and neutrophil marker), CD8 (cytotoxic T‐lymphocyte marker), and IL‐6‐positive cells in the diaphragm of 7‐month‐old WT (*n* = 8), *mdx* (*n* = 8), and *mdx/CD38* ^−/−^ (*n* = 7 or 8) mice. Lower panel: histograms quantifying the percentage of F4/80 (A_1_) *mdx/CD38* ^−/−^ (*n* = 7), LY6 (A_2_) *mdx/CD38* ^−/−^ (*n* = 8), CD8 (A_3_) *mdx/CD38* ^−/−^ (*n* = 7), and IL‐6 (A_4_) *mdx/CD38* ^−/−^ (*n* = 8)‐positive cells. Scale bars: 50 µm.qPCR analysis of mRNA levels of interleukin‐1β (IL‐1ß) (B_1_) and interleukin‐6 (IL‐6) (B_2_), cyclin‐dependent kinase inhibitor 1 (p21) (B_3_), transforming growth factor‐β (TGF‐β) (B_4_), senescence markers (cell‐cycle inhibitor p16, INK4a) (B_5_), and collagen type I alpha 1 chain (Col1a1) (B_6_) in diaphragm of 20‐month‐old WT (*n* = 6), *mdx* (*n* = 5), and *mdx/CD38* ^−/−^ (*n* = 5) mice. Data information: A,B: Each dot of the graphs represents a mouse. A one value/mouse; and B in duplicate. After normality and variance comparison tests, significance was assessed using: A_1–4_: Welch’s ANOVA followed by Welch’s *t*‐tests and B_1–6_: ANOVA followed by Fisher's LSD test. Values are expressed as means ± SEM. Significance: *P < 0.05, **P < 0.01, and ***P < 0.001. Source data are available online for this figure.

Since in *mdx* mice the most injured muscle is diaphragm, which also presents the most active cycles of degeneration/regeneration associated with inflammatory processes (Stedman *et al*, 1991), we decided to evaluate the impact of CD38 deletion on diaphragm inflammation and cellular senescence. To quantify immune cell infiltration, we used a standardized panel of immunostaining markers on sections of diaphragm of 7‐month‐old WT, *mdx*, and *mdx/CD38* ^−/−^ mice: F4/80 (macrophage), Ly‐6G/6C (monocytes, granulocytes, and neutrophils), CD8 (cytotoxic T lymphocytes), and IL‐6‐positive cells (Fig 4A, pictures). The inflammatory markers, almost absent in diaphragm from WT and CD38^−/−^ mice (Appendix Fig S5A), were strongly increased in *mdx* diaphragm and clearly reduced in *mdx/CD38* ^−/−^ mice (Fig 4A, histograms). This indicates that CD38 deletion prevented the chronic inflammatory infiltration observed in *mdx* mouse diaphragm.

We also determined whether this protective effect persisted in older mice (20 months old). We quantified the relative mRNA expression levels of cytokines such as interleukin‐1β and interleukin‐6 (IL‐1ß; IL‐6), cyclin‐dependent kinase inhibitor 1 (p21), transforming growth factor‐β (TGF‐β), and some senescence markers such as cell‐cycle inhibitor p16 (INK4a) and finally Col1A1 (collagen type I alpha 1 chain) for the established chronic fibrosis. All these analyses were performed in WT, *mdx*, and *mdx/CD38* ^−/−^ mice (Fig 4B), and also in *CD38* ^−/−^ mice (Appendix Fig S5B). In the WT strains and CD38^−/−^ mice, all of those markers were not intensively expressed. Interestingly, our data revealed a clear reduction in all these markers in *mdx/CD38* ^−/−^ mice compared with *mdx* mice, confirming that the deletion of CD38 was beneficial for reducing the chronic inflammatory process, even in old *mdx/CD38* ^−/−^ mice (Fig 4B).

### Improvement in function and structure of limb muscles in *mdx/CD38^−/−^* mice

**Figure 5:**
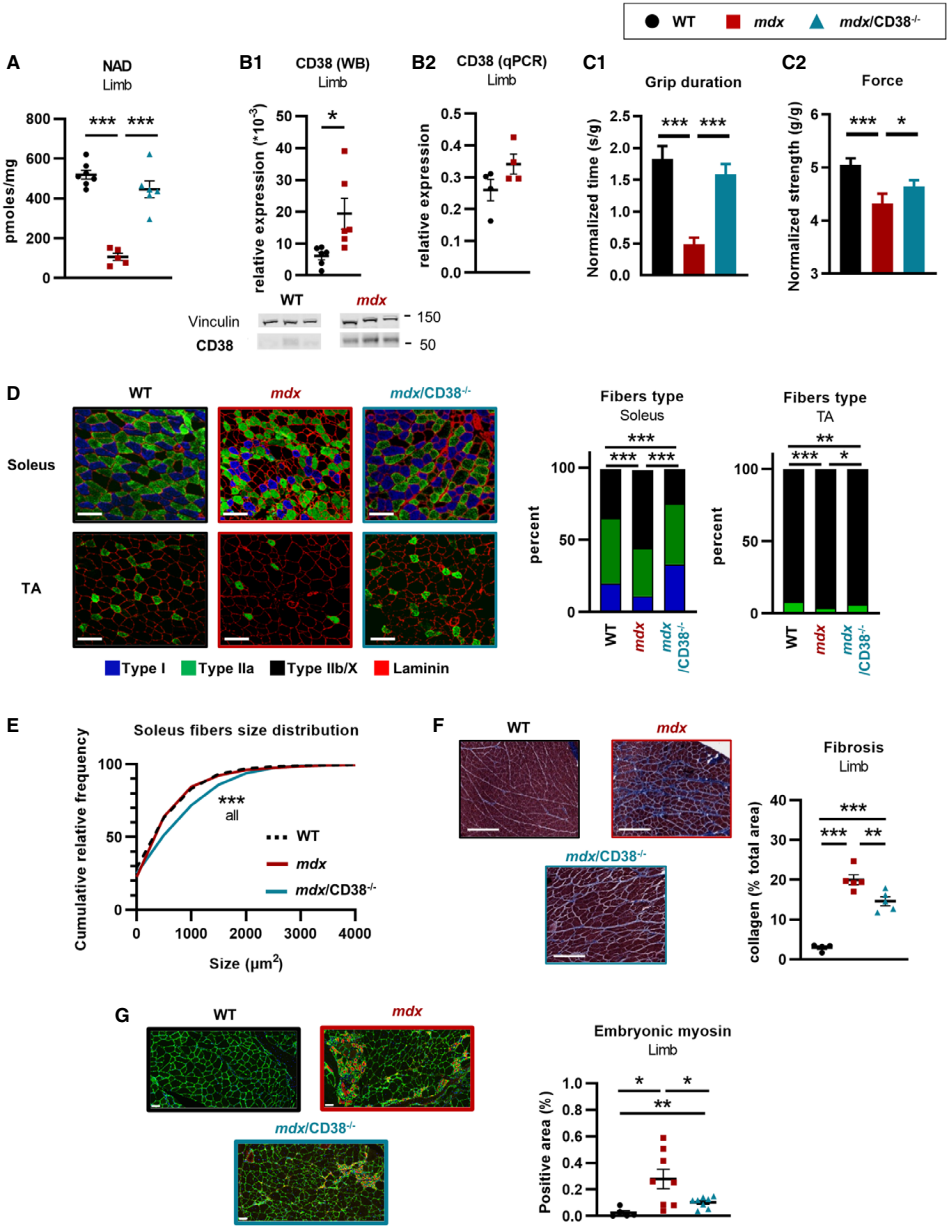
Deletion of CD38 improved skeletal muscle structure and function in *mdx/CD38* ^−/−^ mice NAD^+^ levels in limb muscles of 20‐month‐old WT (*n* = 7), *mdx* (*n* = 5), and *mdx*/*CD38* ^−/−^ (*n* = 6) mice.CD38 expression: Western blot analysis of CD38 (B_1_) protein in limb of WT and *mdx* mice (*n* = 6 per group). Vinculin is used as housekeeping protein control, and the dot plots show the ratio of CD38 to vinculin. qPCR analysis of CD38 mRNA (B_2_) in limb of 20‐month‐old WT and *mdx* mice (*n* = 4 per group).Histograms showing the grip duration (latency to fall) (C_1_) and the limb force (C_2_) measured by a grip test in *WT* (*n* = 89), *mdx* (*n* = 52 and 53, respectively), and *mdx/CD38* ^−/−^ (*n* = 58) mice (age: 9–26 months).Images showing muscle fiber typology revealed by immunostaining. Localization of the slow MyHC (type I) fiber and fast MyHCs (type IIa and IIb/X) fibers, along with laminin (red) on transverse cross sections from soleus and tibialis (TA) of WT, *mdx*, and *mdx/CD38* ^−/−^ mice. Scale bars: 100 µm. Histogram showing the percentage of I, IIa and IIb/X fiber‐type distribution in *soleus* and TA of WT, *mdx*, and *mdx/CD38* ^−/−^ mice. Experiments were performed in WT (*n* = 3), *mdx* (*n* = 5), and *mdx/CD38* ^−/−^ (*n* = 7) mice for soleus; and WT (*n* = 5), *mdx* (*n* = 6), and *mdx/CD38* ^−/−^ (*n* = 6) mice for TA.Fiber size distribution in soleus from WT, *mdx*, and *mdx/CD38* ^−/−^ mice.Images displaying the collagen (blue) revealed by Masson’s trichrome staining in the limb of WT, *mdx*, and *mdx/CD38* ^−/−^ mice. The dot plot shows the quantification of collagen staining area (% total area) in the limb of WT (*n* = 4), *mdx* (*n* = 5), and *mdx/CD38* ^−/−^ (*n* = 5) mice. Scale bars: 200 µm.Embryonic myosin expression revealed by immunostaining along with laminin (green) on transverse cross sections from the limb of WT (*n* = 5), *mdx* (*n* = 8), and *mdx/CD38* ^−/−^ (*n* = 8) mice. Scale bars: 50 µm. Dot plot showing the relative proportion of embryonic myosin area in the limb of WT, *mdx*, and *mdx/CD38* ^−/−^ mice. Data information: Each dot of the graphs represents a mouse. B,C_1_,D,E,G one value/mouse; A in duplicate; and F,C_2_ in triplicate. After normality and variance comparison tests, significance was assessed using: A,F: ANOVA followed by Fisher's LSD test; B_1_: unpaired Welch’s *t*‐test; B_2_ : unpaired Student’s *t*‐test; C_1–2_ : the Kruskal–Wallis test followed by Dunn’s test; D: the chi‐square test; E: the Kolmogorov–Smirnov test; and G: Welch’s ANOVA followed by Welch’s *t*‐tests. Values are expressed as means ± SEM. Significance: *P < 0.05, **P < 0.01, and ***P < 0.001. WT images in 5D and 5G have been redisplayed in S6B and S6E, respectively. Source data are available online for this figure.

Biochemical and structural analyses, as performed in heart and diaphragm, were also conducted on limb muscles in WT, *CD38* ^−/−^ (Appendix Fig S6), *mdx*, and *mdx/CD38* ^−/−^ mice. Similar to the heart and diaphragm, limb from 20‐month‐old *mdx* mice showed a dramatic deficit (around 80% reduction) in NAD^+^ levels, which was fully restored to normal values in *mdx/CD38* ^−/−^ mice (Fig 5A). This was associated with a twofold to threefold increase in CD38 protein expression compared with WT mice, without changes in the levels of CD38 mRNA (Fig 5B). We then explored the effects of the deletion of CD38 on muscle function in 9‐ to 26‐month‐old *mdx* mice. Compared with WT, *mdx* mice displayed a 73% reduction in the grip duration (resistance to fatigue) and a close to 15% reduction in the limb maximum force (Fig 5C). These deficiencies were significantly improved in *mdx/CD38* ^−/−^ mice (Fig 5C). We also investigated the relative proportion of each fiber type (I, IIa, and IIb/x) in sections of soleus and tibialis anterior (TA). The relative proportion of type IIa and IIb/x fibers was mostly reversed from *mdx* profile to WT profile in *mdx/CD38* ^−/−^ mice (Fig 5D), with, in addition, an increase in the proportion of slow aerobic type I fibers in the soleus, known to be more resistant to degeneration (Webster *et al*, 1988; Consolino & Brooks, 2004; Selsby *et al*, 2012; Chalkiadaki *et al*, 2014) (Fig 5D). In soleus, the mean fiber size distribution was not different between WT and *mdx* mice, but larger size fibers were observed in *mdx/CD38* ^−/−^ mice (Fig 5E), which could explain a better muscle function. For TA, no difference was observed concerning the fiber size of the three groups (not illustrated).

Infiltration of limb by collagen, the marker of fibrosis, was visualized using Masson’s trichrome staining. As shown in Fig 5F, *mdx* mice limb displayed collagen infiltration (blue staining), which was clearly reduced in *mdx/CD38* ^−/−^ mice. Similar to diaphragm, muscular degeneration triggers myogenesis in the limb to compensate for the muscle loss. We therefore evaluated myogenesis through embryonic myosin immunostaining: It was totally absent in sections from WT mice limb, clearly present in *mdx* mice, and strongly reduced in *mdx/CD38* ^−/−^ mice (Fig 5G), which supports again the protective effect of CD38 deletion in *mdx* mice.

Therapeutic potential of drugs inhibiting CD38 in two mouse models, the *mdx* and *mdx/utr* ^−/−^ mice, and in human DMD myotubes.

### Enhancement of skeletal muscle performance following treatment with CD38 inhibitors in *mdx* and *mdx/utr^−/−^* mice

**Figure 6:**
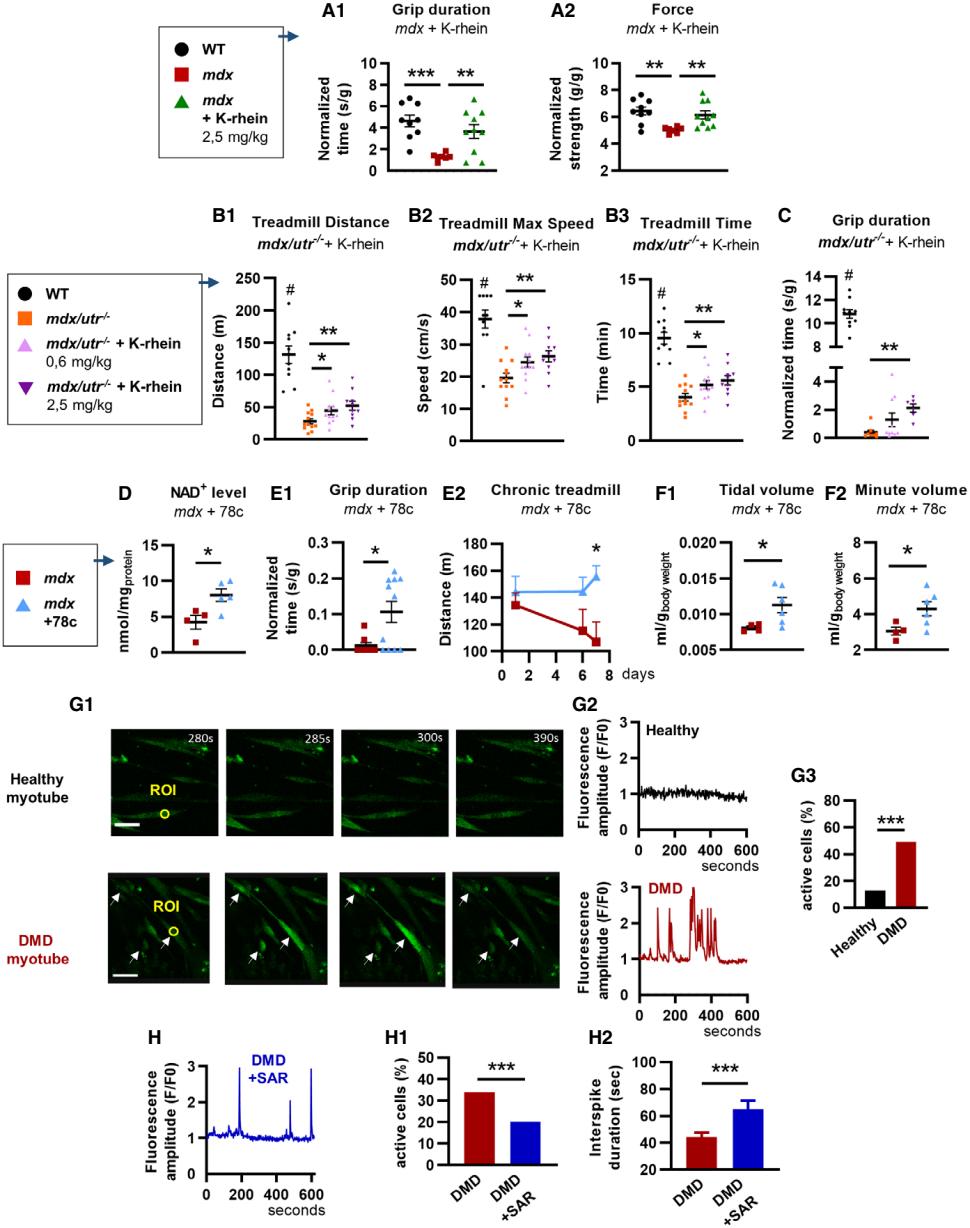
Beneficial effects of the pharmacological inhibition of CD38 in *mdx* mice and human DMD myotubes AYoung *mdx* mice and treatment with a CD38 inhibitor. 6‐week‐old *mdx* mice were treated for 5 weeks with K‐rhein at 2.5 mg/kg/d by intraperitoneal injection. Dot plots showing measurement of the grip duration (A_1_) and the force (A_2_) of WT (*n* = 9), *mdx* (*n* = 7), and K‐rhein*‐*treated *mdx* (*n* = 10) mice.B, CNewborn double‐knockout utrophin–dystrophin (*mdx/utr* ^−/−^) mice and treatment with an CD38 inhibitor. *Mdx/utr* ^−/−^ mice were subcutaneously injected with K‐rhein (0.6 and 2.5 mg/kg/d) for 4 weeks. B: Dot plots showing the treadmill performances of K‐rhein*‐*treated *mdx/utr* ^−/−^ mice: distance traveled (B_1_), maximum speed reached (B_2_), and maximum running time (B_3_) (WT (NaCl) and *mdx/utr* ^−/−^ + K‐rhein 2.5 mg/kg/d, *n* = 10 mice per group; *mdx/utr* ^−/−^(NaCl) and *mdx/utr* ^−/−^ + K‐rhein 0.6 mg/kg/d, *n* = 12 mice per group). C: Measurement of the grip duration (grid test) in WT (*n* = 10), *mdx/utr* ^−/−^ (*n* = 8), and K‐rhein*‐*treated *mdx/utr* ^−/−^ mice (*n* = 10 and 6 mice, respectively, for the 0.6 mg/kg/d and 2.5 mg/kg/d doses).D–FAdult *mdx* mice and long‐term treatment with an CD38 inhibitor. *Mdx* mice were evaluated after 6 months of intraperitoneal injection with the CD38 inhibitor 78c (10 mg/kg/d). D: Histogram showing NAD^+^ levels in the limb of *mdx* (*n* = 4) and 78c‐treated *mdx* (*n* = 5) mice. E: Histogram showing grip duration (E_1_) (*n* = 8 *mdx* and *n* = 11) in the inverted grid test and performances in chronic treadmill test (E_2_) at days 1, 6, and 7 after treatment of *mdx* (*n* = 5 except for D7, *n* = 4) and 78c*‐*treated *mdx* (*n* = 7) mice. F: Dot plots showing barometric plethysmography measures of the tidal (F_1_) and minute (F_2_) volumes of *mdx* (*n* = 4) and 78c*‐*treated *mdx* (*n* = 6) mice.GTime‐lapse confocal imaging of calcium dynamics in human healthy and DMD myotubes loaded with the Ca^2+^‐sensitive dye Fluo‐4 (white arrows show the active cells) (G_1_). Traces illustrating recordings from region of interest (ROI) in an inactive healthy myotube (black line) and in a DMD myotube displaying Ca^2+^ spiking activity (red line) (G_2_). Histogram showing the percentage of myotubes displaying spontaneous Ca^2+^ waves (G_3_): healthy myotubes (*n* = 91 cells) and DMD myotubes (*n* = 186 cells). Scale bars: 200 µm.HHuman DMD myotubes treated by SAR650984 (SAR, isatuximab), a humanized anti‐CD38 antibody. Fluorescence trace illustrating a recording of DMD myotubes treated with 10 µg/ml of SAR (blue). Histogram showing the percentage of spontaneous Ca^2+^ waves in DMD myotubes untreated (*n* = 740 cells) or treated with 10 µg/ml of SAR (*n* = 279 cells) (H_1_). Histogram showing the Ca^2+^ wave interspike duration (interval between spikes) in myotubes treated with 10 µg/ml of SAR (*n* = 43 cells vs 91 for the untreated DMD myotubes) (H_2_). Data information: A–F: Each dot of the graphs represents a mouse. A_1_,B one value/mouse; D,E,F in duplicate; and A_2,_C in triplicate. After normality and variance comparison tests, significance was assessed using: A_1_: Welch’s ANOVA followed by Welch’s *t*‐tests; A_2_, B_1–3_: ANOVA followed by Fisher's LSD test; C: the Kruskal–Wallis followed by Dunn's test; D: unpaired Student’s *t*‐test; F_2_,E_1_: an unpaired Mann–Whitney test; E2: two‐way ANOVA; F_1_,H_2_: unpaired Welch’s *t*‐test; and G_3_,H_1_: the chi‐square test Values are expressed as means ± SEM. Significance: *P < 0.05, **P < 0.01, ***P < 0.001, and ^#^ *P* < 0.001 vs *mdx/utr* ^−/−^. Source data are available online for this figure.

The dramatic reduction in the dystrophic phenotype in *mdx/CD38* ^−/−^ mice prompted us to explore *in vivo* the therapeutic potential of drugs inhibiting CD38 in two murine models: the *mdx* and the *mdx/utr* ^−/−^ mice. We first treated 2‐month‐old *mdx* mice for 5 weeks with K‐rhein (2.5 mg/kg/d), a CD38 inhibitor derived from flavonoids (Blacher *et al*, 2015). Compared with WT mice, the *mdx* control mice (NaCl 0.9%) displayed reduced performance in grip duration and force, whereas K‐rhein‐treated *mdx* mice displayed restored grip duration and force (Fig 6A). Additionally, we observed no effect of K‐rhein on the muscular performances of WT mice (Appendix Fig S7A). Finally, we evaluated the potential toxicological effects of K‐rhein. Key histological features in liver and kidney from control (NaCl) and 5‐week K‐rhein‐treated *mdx* mice (9–10 mice per group) were analyzed. No sign of toxicity was observed in the K‐rhein‐treated *mdx* mice compared with untreated *mdx* mice (Appendix Fig S8).

Furthermore, we investigated the therapeutic benefit of K‐rhein in the double‐knockout utrophin–dystrophin (*mdx/utr* ^−/−^) mouse, a model that develops a more severe muscular dystrophic phenotype than the *mdx* model, with a reduced life expectancy and growth, associated with severe motor activity impairments (Deconinck *et al*, 1997; Grady *et al*, 1997; Goyenvalle *et al*, 2015). Newborn four/five‐day‐old *mdx/utr* ^−/−^ mice were treated 3 days a week with subcutaneous injections of K‐rhein (0.6 or 2.5 mg/kg/d or NaCl 0.9%) and were evaluated after one month of treatment for their performance on a treadmill and grip tests. In the exhaustion treadmill test, we found that *mdx/utr* ^−/−^ mice performance was improved by the K‐rhein treatment, with a dose‐dependent effect on the distance traveled, the maximum speed reached, and the maximum running time (Fig 6B). Performances in the grip duration were also improved in *mdx/utr* ^−/−^ mice (Fig 6C).

We then evaluated the functional benefit of a long‐term treatment (6 months) with the CD38 inhibitor 78c derived from 4‐aminoquinolines (Hogan *et al*, 2019) (10 mg/kg/dose, twice daily) in adult *mdx* mice. At the time of the evaluation, the mice were around 15 months old. The 78c‐treated *mdx* mice showed higher NAD^+^ levels in the gastrocnemius than in *mdx*‐untreated mice (Fig 6D). Consistent with the data obtained in K‐rhein*‐*treated *mdx* mice, the 78c‐treated *mdx* mice showed better performance in the grip test than in the *mdx*‐untreated mice (Fig 6E). Additionally, the 78c treatment provided protection against the deleterious effects of a chronic treadmill exercise (known to worsen the *mdx* phenotype (Hyzewicz *et al*, 2015) performed at days 1, 6 and 7 after treatment, since 78c‐treated *mdx* mice showed an improved performance at day 7, compared with *mdx*‐untreated mice, which showed a progressive altered performance (Fig 6E). Finally, the 78c treatment also provided a significant improvement in the respiratory function with an increase in the tidal volume and the minute volume, evaluated by plethysmography (Fig 6F).

### Pharmacological inhibition of spontaneous Ca^2+^ activity in human DMD myotubes treated with a humanized antibody against CD38

To test whether CD38 inhibition could also be beneficial in human myotubes, we used SAR650984 (isatuximab), an anti‐CD38 monoclonal antibody, on human DMD myotubes, also known to display pathological spontaneous Ca^2+^ activity (Vianello *et al*, 2014). The spontaneous Ca^2+^ waves were recorded for 10 min, and 50% of the DMD myotubes displayed frequent spontaneous Ca^2+^ waves compared with only 13% of the healthy myotubes (Fig 6G). DMD myotubes treated 48 h with SAR650984 (10 µg/ml) showed a reduced number of myotubes displaying spontaneous Ca^2+^ waves (Fig 6H). In treated DMD myotubes still presenting spontaneous Ca^2+^ waves, SAR650984 increased by 47% the interspike duration of these Ca^2+^ waves (Fig 6H), thus reducing their frequency, but without changing their amplitude. We found also that DMD myotubes have lower NAD^+^ levels than healthy myotubes (Appendix Fig S7B). However, SAR650984‐treated myotubes (50 µg/ml) (Appendix Fig S7B) failed to change the endogenous NAD^+^ levels both in healthy and in DMD myotubes. This could be explained by the mechanism of action of isatuximab. Indeed, it has been recently reported that CD38 antibody‐dependent cell‐mediated cytotoxicity (ADCC), or complement‐dependent cytotoxicity (CDC), may act by targeting the cADPR production rather than the NAD^+^ glycohydrolase activity, which represents the main enzyme reaction catalyzed by CD38 (Berthelier *et al*, 1998; Baum *et al*, 2020).

## Discussion

In summary, our data based on the genetic deletion of CD38 and its pharmacological inhibition in different DMD models (i) clearly indicate that CD38 is an important contributor to the NAD^+^ deficit and to the Ca^2+^ dysregulation, two major features in this disease, and (ii) strongly support our innovative strategy to treat DMD by a single common pharmacological approach reducing simultaneously the Ca^2+^ overload and the NAD^+^ deficit, leading to an important improvement in the DMD phenotype.

By genetic deletion of CD38 in *mdx* mice, we obtained a full restoration of a normal cardiac function, with normal values of the two parameters altered in *mdx* mice, namely the LVEF and inner left ventricular diameters (LVSD and LVDD), evaluated by echocardiography. We also found reduction in plasma levels of cardiac stress markers and reduced heart fibrosis in *mdx/CD38* ^−/−^ mice. In addition, hearts from *mdx/CD38* ^−/−^ mice were clearly resistant to ß‐adrenergic‐induced stress, since all the mice survived in this group, while 50% of the *mdx* mice tested died. Not only the *mdx/CD38* ^−/−^ mice survived but they also showed neither sign of cardiac hypertrophy nor increase in plasma levels of cardiac stress markers.

Importantly, besides the crucial protection of the cardiac function, deletion of CD38 in *mdx*/*CD38* ^−/−^ mice also provided an overall improvement in skeletal muscle structure and function in the diaphragm and the limb. Looking at the respiratory function, *mdx/CD38* ^−/−^ mice showed a clear improvement in various parameters such as inspiratory, expiratory, and relaxation times, and this was accompanied by a strong reduction in markers of inflammation and cellular senescence. In the limb, the well‐known deficiencies of *mdx* mice in the grip duration (resistance to fatigue) and in the limb maximum force were significantly improved in *mdx/CD38* ^−/−^ mice. This was associated with numerous histological therapeutic effects of CD38 deletion, found in both diaphragm and limb muscles: a fiber‐type distribution close to a WT mouse profile, with an increased proportion of more resistant slow oxidative fibers in the limb, accompanied by reduced fibrosis and muscle regeneration (evaluated by embryonic myosin expression), suggesting an overall protective and beneficial role of CD38 deletion in the skeletal muscles of the *mdx* mouse.

We then used a pharmacological approach to demonstrate the therapeutic potential of drugs inhibiting CD38 in *mdx* mice, *mdx/utr* ^−/−^ mice, and myotubes from DMD patients. In our study, young *mdx* mice treated for 5 weeks with K‐rhein displayed restored limb muscle performance (grip duration and force), which were comparable to WT mice. Similarly, juvenile *mdx/utr* ^−/−^ mice treated 4 weeks with K‐rhein displayed an improvement in their muscular performance in the grip duration and in a dose‐dependent manner in a treadmill exercise. The extent of the improvement was partial, compared with WT mice, likely due to the very severe phenotype displayed by the *mdx/utr* ^−/−^ mice. Finally, we also show in our study that long‐term treatment over a period of 6 months with 78c, a CD38 inhibitor with a different chemical structure from K‐rhein, improved the performances of *mdx* mice in the grip test, in the chronic exercise on a treadmill, and also their respiratory function.

CD38 is a ubiquitous enzyme with NAD^+ ‐^glycohydrolase activity, and its distribution has been extensively studied. CD38 is reported to be expressed not only in myocytes (Guedes *et al*, 2015; Lee *et al*, 2015; Lin *et al*, 2017; Park *et al*, 2018) but also in endothelial cells, fibroblasts, and immune cells (Partida‐Sánchez *et al*, 2001; Partida‐Sanchez *et al*, 2007; Boslett *et al*, 2018; Tarragó *et al*, 2018). Although we cannot exclude a role for CD38 in non‐muscle cells, our results on improved NAD^+^ levels were measured in subcellular compartments of isolated cardiomyocytes from *mdx/CD38* ^−/−^ mice, and on Ca^2+^ signaling, in isolated cardiomyocytes from *mdx/CD38* ^−/−^ mice; human DMD myotubes point to an important role of CD38 in muscle functioning. In our study, CD38 expression in young *mdx* mice is not upregulated but its deletion is clearly beneficial. However, we observed in aged *mdx* mice higher expression of CD38 in heart, diaphragm, and limb. Regarding the mechanisms involved in this high CD38 expression that may occur in aged *mdx* mice, many studies have shown that reactive oxygen species (Kumasaka *et al*, 1999; Okabe *et al*, 2000; Wilson *et al*, 2001; Zhang *et al*, 2004) and inflammatory molecules can regulate CD38 expression and/or activity (Bauvois *et al*, 1999; Deshpande *et al*, 2003; Kang *et al*, 2006; Karakasheva *et al*, 2015). Interestingly, it has been shown that during aging, senescent cells can secrete inflammatory molecules leading to an increase in CD38 expression, which contributes to the NAD^+^ decline linked to aging (Aksoy *et al*, 2006; Camacho‐Pereira *et al*, 2016; Chini *et al*, 2019, 2020). Since DMD displays an important oxidative stress and an important inflammatory response (Lawler, 2011; Terrill *et al*, 2013; Rosenberg *et al*, 2015), it is therefore likely that in this disease, these molecules play a major role in the upregulation of CD38 expression that might occur in muscle, endothelial cells, or immune cells during aging. We found in heart tissue that the majority of CD38 surface expression measured using flow cytometry is in endothelial cells. However, for the endothelial cells, the number of CD38^+^ cells has a trend to be lower in *mdx* mice, with no or little change observed in hematopoietic cells, or in other cells expressing CD38 (Appendix Fig S7C). Thus, it is possible that in DMD, the inflammatory and fibrotic stages may have an influence on the type of cells that express CD38 in the tissue. However, regardless of the specific cells that express CD38, it remains clear that its deletion has brought, directly or indirectly, numerous beneficial effects in different types of muscular tissues or type of isolated myocytes in three different DMD models, namely the *mdx* and the *mdx/utr* ^−/−^ mice, and the myotubes from DMD patients.

DMD is also characterized by an important Ca^2+^ homeostasis dysregulation with an excessive Ca^2+^ influx through activation of plasma membrane channels, such as TRP channels, associated with a high RyR activity (Wang *et al*, 2005; Williams & Allen, 2007; Bellinger *et al*, 2009; Fauconnier *et al*, 2010; Allen *et al*, 2016; Espinosa *et al*, 2016), importantly also linked to inflammation (Bellinger *et al*, 2009; Tidball & Villalta, 2009; Altamirano *et al*, 2012). Importantly, RyR dysregulation has been shown to promote cardiomyopathy and skeletal muscle degeneration in the *mdx* mouse, and for instance, various strategies aiming to reduce the Ca^2+^ leaks from RyRs showed a significant improvement in its phenotype (Bellinger *et al*, 2009; Fauconnier *et al*, 2010; Sarma *et al*, 2010; Ather *et al*, 2013). Our results obtained on cardiomyocytes from *mdx/CD38* ^−/−^ mice show that CD38 is also a key contributor to the oversensitization of RyRs in DMD. Indeed, we found that the spontaneous Ca^2+^ spark and wave activity was restored to WT values associated with restored post‐rest potentiation and fractional release values in *mdx/CD38* ^−/−^, indicating an important normalization of the RyR sensitivity. From our data, it appears that the basal level of cADPR, a RyR modulator produced by CD38, was increased in *mdx/CD38* ^−/−^ muscle tissues, which could be due to a higher SARM1 activity. On the contrary, the ADP‐ribose level, the most abundant product of CD38, was dramatically reduced. This last result suggests that the beneficial effect of CD38 deletion could be linked to the ADP‐ribose action, possibly through the TRPM2 channel. Indeed, in various cell types including cardiomyocytes, ADP‐ribose is known to trigger Ca^2+^ influx through TRPM2 located at the plasma membrane, which could lead to RyR sensitization through Ca^2+^‐induced Ca^2+^ release (Miller *et al*, 2013; Fliegert *et al*, 2017b; Lee *et al*, 2018).

Regarding NAD^+^ metabolism, CD38 deletion provided a full restoration of the NAD^+^ levels in all muscle types examined in *mdx* mice. Our study shows, in a specific pathological context, the importance of CD38 in the excessive cellular NAD^+^ consumption. The major improvement in the NAD^+^ levels and the reduced NAM levels observed by inhibiting CD38 are expected to stimulate several beneficial pathways. These pathways could contribute to the positive effects found in the *mdx/CD38* ^−/−^ mice, notably through the activation of sirtuins and its downstream target, the peroxisome proliferator‐activated receptor‐gamma coactivator 1α (PGC‐1α) (Aksoy *et al*, 2006; Barbosa *et al*, 2007; Guan *et al*, 2016; Tarragó *et al*, 2018; Wang *et al*, 2018), both known to improve some aspects of the *mdx* mouse phenotype, including reduced fibrosis and improved treadmill performances (Handschin *et al*, 2007; Chalkiadaki *et al*, 2014; Capogrosso *et al*, 2016; Ryu *et al*, 2016; Kuno *et al*, 2018; Sebori *et al*, 2018), as observed in the present study. Moreover, we also observed that CD38 deletion led to a reversal of the altered muscular fiber phenotype seen in the *mdx* mouse close to the WT mouse phenotype, notably with an increase in the proportion of slow oxidative fibers in the soleus. This unexpected result might be linked to PGC‐1α activity. Indeed, PGC‐1α has been proposed to induce, in skeletal muscle cells of the *mdx* mouse, a switch from glycolytic (fast anaerobic fibers) to oxidative metabolism (slow aerobic fibers), which may contribute to alleviate the dystrophic phenotype of the *mdx* mouse (Hohenegger *et al*, 2002; Lin *et al*, 2002; Selsby *et al*, 2012; Ljubicic *et al*, 2014; Capogrosso *et al*, 2016).

It is important to note that studies aiming at tissue NAD^+^ level repletion by administration of NAD^+^ precursors, such as nicotinamide riboside (NR) or NMN, observed limited increases in NAD^+^ levels, possibly due to the endogenous CD38 activity, which is known to degrade both NAD^+^ and its precursor NMN (Tarragó *et al*, 2018; Chini *et al*, 2020). Therefore, despite considerable efforts, none of the treatments targeting either NAD^+^ deficit or RyR activity have been so far successfully used clinically for DMD. One reason may lie not only on the lack of knowledge on the precise mechanisms involved in these two key parameters but also on the fact that none of these studies treated these two aspects simultaneously. In our study, CD38 deletion in the *mdx* mouse, simultaneously restored NAD^+^ levels, reduced RyR activity and was very beneficial to *in vivo* muscle function, notably for the cardiac function that was totally preserved. Moreover, the beneficial results obtained for the skeletal muscle function with K‐rhein and 78c, two CD38 inhibitors (Hogan *et al*, 2019) on two different models of DMD, namely the *mdx* and the *mdx/utr* ^−/−^ mice, and the effect of isatuximab on human DMD myotubes clearly reassert our results on CD38 deletion in *mdx* mice. Overall, CD38 appears to be a major contributor to the onset of cardiac and skeletal muscle dysfunction in DMD.

Since anti‐CD38 therapy is already available, such as isatuximab (SARCLISA^®^), which is approved for the treatment of adults with relapsed/refractory multiple myeloma, this study proposes that CD38 could be a possible therapeutic target in DMD, especially for one of its main features, namely the dilated cardiomyopathy. Importantly, all anti‐CD38 antibodies currently used in the clinic for multiple myeloma do not target the catalytic site, but rather an allosteric site, and are potentially cytotoxic through mechanisms such as antibody‐dependent cell‐mediated cytotoxicity (ADCC) or complement‐dependent cytotoxicity (CDC). Therefore, the development of antibodies directly targeting the CD38 catalytic site with reduced cytotoxic effect should now be a priority (Chini *et al*, 2018) for further development of such a therapy for DMD. Finally, this study also potentially concerns other pathologies, such as other dilated cardiomyopathies and heart failure, which combine Ca^2+^ dysregulation and NAD^+^ deficit, for which an anti‐CD38 therapy could be highly relevant.

## Materials and Methods

### Animals: *mdx/CD38* ^−/−^ mice generation

The colony was set up by crossing *mdx* mice with *CD38* ^−/−^ mice to generate *mdx/CD38* ^−/−^ mice. *Mdx* mice (C57BL/10ScSn‐*Dmdmdx*/J) were purchased at the Jackson laboratory. *CD38* ^−/−^ mice with a deletion of exons 2 and 3 in the CD38 gene were obtained from the Lund and Randall Laboratory [University of Alabama–Birmingham (UAB), AL, USA]. The *CD38* ^−/−^ mice have been backcrossed 10 times onto the C57BL/6 inbred strain background and were shown to exhibit no residual enzymatic activity *in vitro* (Partida‐Sánchez *et al*, 2001). For genotyping of WT/*CD38* ^−/−^ and *mdx/CD38* ^−/−^ mice, genomic tail DNA extraction was performed as described: Briefly, in 1.5‐ml Eppendorf tube 150 µl NaOH (50 mM) was added to mice tails and incubated for 30 min at 100°C, and then, 150 µl of 1 mol/L Tris–HCl (pH=8.2) was added into the tube and mixed well. The supernatant was used for PCR analysis. For *mdx* genotyping, we used the protocol described by Shin *et al* (2011) (134 bp wild type, 117 bp mdx) using three primers: common, forward—5’‐GCGCGAAACTCATCAAATATGCGTGTTAGTGT‐3’; wild type, reverse—5’‐GATACGCTGCTTTAATGCCTTTAGTCACTCAGATAGTTGAAG‐3’; and mutant, reverse—5’‐CGGCCTGTCACTCAGATAGTTGAAGCCATTTTA‐3’. For CD38, we used *forward CD38 primer 5’‐CTCTCTTTTGGAGCAAATCAA‐3’* and *reverse CD38 primer* 5’‐GTACTAGGGTCTCCACACCAC‐3’; ad for GAPDH, we use forward GAPDH primer 5’‐TGACGTGCCGCCTGGAGAAA‐3‘ and reverse GAPDH primer 5’‐AGTGTAGCCCAAGATGCCCTTCAG‐3’. The PCR products were analyzed in a 2% agarose gel. C57BL/6 mice and *CD38* ^−/−^ mice were bred in our animal facility at the Neuroscience Paris‐Saclay Institute. *Mdx/utr* ^−/−^ mice were generated by crossing (Utr^+/‐^; Dys^−/−^) mice (JB10ScSn. Cg‐Utrntm1KedDmdmdx/J; Jackson Laboratory). For genotyping, genomic DNA was extracted from mouse tails using NaOH (50 mM) for 30 min at 100°C. The utrophin gene was amplified by PCR, and products were analyzed in agarose gel electrophoresis as described in Forand *et al* (2020). All the experiments with *mdx/utr* ^−/−^ mice were performed in blind.

Animal groups were WT (C57BL/10), *mdx*, WT/*CD38* ^−/−^ (name of the littermate *CD38* ^−/−^), *mdx/CD38* ^−/−^ (male), and *mdx/utr* ^−/−^ (male and female) mice. The mice contained in a home cage were randomly assigned to an experimental group; the animal groups were matched by age. After performing the different functional analyses, the animals were killed by cervical dislocation. The heart, limb, and diaphragm were dissected and frozen for structural analyses. The animals were weighed before each experiment. Animal care and experimental procedures complied with the European Communities Council Directive (CEE 86/609/EEC), EU Directive 2010/63/EU, and local ethics committee (Paris Centre et Sud, N°59). The protocols were approved by the French Departmental Direction of Animal Protection (2015‐11 #1027). For experiments with 78c, the protocol was IACUC (institutional animal care and use committee)‐approved. Some animals have been removed from the experiments due to animal death or removal in accordance with the animal welfare guidelines for reducing the suffering of the animals during the experimental procedure.

### Combined forelimb and hindlimb grip strength measurement

Grip strength was measured using a grip strength meter (Bioseb, Vitrolles, France). The apparatus consisted of a grid connected to a digital dynamometer. The animals were gently lowered over the top of the grid so that both sets of front paws and hind paws could grip the grid. While the torso of the animal was kept parallel to the grid, the mouse was gently pulled back by the tail until it released its grip. This procedure was repeated three times, and the maximum and the mean values have been normalized to mouse body weight.

### Inverted grid and Treadmill tests

#### Inverted grid

To measure muscle resistance to fatigue, mice were placed individually on a cage wire grid, 35 cm above a table. After slowly turning the grid through 180°, the time the mice continued to grip the grid was monitored (the grip duration), a maximum score of 180 s being given if the animal did not fall. This procedure was repeated three times. The values were averaged and normalized to mouse body weight.

#### Treadmill

For *mdx* mice, chronic treadmill tests were performed as previously described (Tarragó *et al*, 2018). For *mdx/utr* ^−/−^ mice, acclimatization was performed with one session on a motorized treadmill at 5 m/min for 10 min. On the day of the experiment, the mice ran for 5 min at 5 m/min before starting recording, and then, the speed of the treadmill was incremented by 2 m/s every 30 s, until the mouse showed obvious signs of fatigue and could not continue the test. The distance traveled, the speed reached, and the duration of the run were recorded.

### Barometric plethysmography

The respiratory function of mice was evaluated by whole‐body plethysmography using an EMKA Technologies plethysmograph, essentially as described by TREAT‐NMD. Briefly, unrestrained conscious mice were placed in calibrated animal chambers and the pressure difference between the reference and animal chambers was measured using a pressure transducer. Mice were allowed to acclimate in the chambers for 45 min at stable temperature and humidity. Data were then collected every 5 s using the iox2 software (version 2.8.0.19; EMKA Technologies). The inspiration time (TI) was defined as the time from the start of inspiration to the end of inspiration, and the expiration time (TE) was defined as the time from the start of expiration to the end of expiration. The relaxation time (RT) was defined as the time from the start of expiration to the time when 65% of the total expiratory pressure occurred. Pause and enhanced pause (penh) were defined and calculated by the following formulas: pause = (TE – RT)/RT and penh = (PEP/PIP) × pause, where PEP is peak expiratory pressure, and PIP is peak inspiratory pressure. The value of each parameter was calculated from an average of 60 recordings of 5 s representing a total of 5 min. Inclusion criteria for each recording were >8 respiration events by 5 s and > 80% of success rate as measured. In the case of experiments of mdx mice treated with 78c, 78c was provided in the food (600 ppm) for about 6 months. At the time of analysis, mice were 17–21 months old.

### Echocardiography procedure

The procedure was done under isoflurane anesthesia. Anesthesia doses were kept to the lowest possible levels, usually 5% isoflurane for induction and 1–1.5% isoflurane during measurements. Animals were placed on a heating pad to maintain a constant body temperature (37°C), and their rectal temperature was monitored throughout the experiment. Echocardiography was performed using a high‐resolution ultrasound system (Logiq 9, GE, France) with a 36‐MHz scan head. Each animal was shaven from the left sternal border to the left axillary line with depilatory cream before the examination. Each set of measurements was obtained from the same cardiac cycle. At least three sets of measurements were obtained from three different cardiac cycles. The left ventricular end‐diastolic diameter (LVEDD), posterior wall thickness, and interventricular septal wall thickness were measured using the leading‐edge convention of the American Society of Echocardiography from M mode. The LVEDD was measured, from an M‐mode short‐axis view of the left ventricle at the papillary muscle level. Shortening fraction and left ventricular ejection fraction (LVEF) were calculated from the M mode. Aortic velocity integral (VTI) was recorded during the procedure from Doppler echocardiography. Mitral inflow Doppler pattern was recorded (peak E, peak A, and deceleration time) from a 4‐chamber apical view. The left ventricular systolic intervals of the isovolumic contraction time (IVCT), the ventricular ejection time (ET), and the diastolic interval of the isovolumic relaxation time (IVRT) were measured for the Tei index calculation. Measurements were made for aortic and mitral blood flows from an apical 4‐chamber modified view. The Tei index was calculated as the ratio of (IVCT + IVRT) to systolic ejection time (ET). Cardiac output (CO) was defined as stroke volume x heart rate. The shortening fraction (%) was calculated by the formula: (LVEDD‐LVESD)/LVEDD × 100. LV myocardial volume (LVV), and LV end‐diastolic (EDV) and end‐systolic (ESV) volumes were calculated using a half ellipsoid model of the LV. From these volumes, LV ejection fraction (%) was calculated by the formula: (EDV‐ESV)/EDV × 100. These experiments were performed in blind.

### β‐adrenergic stress test

β‐adrenergic stimulation was sustained by subcutaneous injection of isoproterenol under light isoflurane anesthesia (induction at 5%). Mice were randomly assigned to receive a daily subcutaneous injection of isoproterenol (2.5 mg/kg/d) or vehicle (0.9% NaCl) for 10 days. At the end of the infusion period, surviving mice were killed, body and heart mass were plotted, and histological analyses (Masson’s trichrome stain) were performed on hearts to evaluate the induced necrosis.

### Western blot

Protein extracts were obtained from pooled muscle sections treated with RIPA lysis and extraction buffer (Thermo Fisher Scientific, USA) complemented with SDS powder (5% final) (Bio‐Rad, France), and the total protein concentration was determined with the BCA Protein Assay Kit (Thermo Fisher Scientific, USA). Samples were denatured at 100°C for 3 min, and 7.5 μg of protein was loaded onto NuPAGE 4–12% Bis‐Tris protein gels (Invitrogen) following the manufacturer’s instructions. CD38 protein was detected by probing the membrane with an anti‐CD38 primary polyclonal rabbit antibody (1/1000; ab216343; Abcam, Cambridge, UK), and vinculin was detected as internal control with the hVin‐1 primary antibody (1/20000: Sigma), followed by incubation with a goat anti‐rabbit secondary antibody (1/5000; IRDye 800CW Goat anti‐Rabbit IgG, Li‐COR, Germany). Bands were visualized using the Odyssey CLx System (Li‐COR, Germany). Quantifications were achieved with corresponding WT tissues and normalized to internal control (vinculin) (Empiria Studio, Li‐COR, Germany).

### Quantification of mRNA by qPCR

RNA was isolated from cells and mouse tissues using Qiagen RNeasy kits. cDNA was synthesized using the Applied Biosystems High Capacity cDNA Reverse Transcription Kit. Real‐time qPCR was performed using commercially available TaqMan gene expression probes (Applied Biosystems, USA), according to the manufacturer’s instructions on a Bio‐Rad CFX384 thermal cycler. The relative mRNA abundance of target genes was calculated by the 2(−ΔΔCq) method. Probes used were as follows: Cd38 (Mm01220904_m1), Cdkn1a (p21) (Mm04205640_g1), Cdkn2a (p16) (Mm00494449_m1), Col1a1 (Mm00801666_g1), Il1b (Mm00434228_m1), IL‐6 (Mm00446190_m1), Nampt (Mm00451938_m1), Parp1 (Mm01321084_m1), Sarm1 (Mm00555617_m1), Tbp (Mm00446971_m1), and Tgfb1 (Mm01178820_m1). TATA‐binding protein (TBP) is used as housekeeping gene.

### Flow cytometry analysis

Mice were euthanized using carbon dioxide, and the heart was excised following perfusion with ice‐cold HBSS (Gibco). Heart tissue was then cut into 6–8 smaller pieces and transferred to MACS C‐tube (Miltenyi Biotec, USA) containing 5 ml collagenase solution containing 500 U/mL collagenase type II (Worthington Biochemical Corporation) in HBSS. The C‐tube was then transferred to gentleMACS Dissociator (Miltenyi Biotec, USA), and program 37_multiE_01 was used to prepare the single‐cell suspension. After adding 10 ml HBSS, the homogenate was passed through 70‐µm cell strainer and centrifuged at 350 *g* for 10 min at 4°C. The debris were removed using debris removal solution following the manufacturer’s instructions (Miltenyi Biotec, USA). Following debris removal, the RBCs were eliminated using ACK lysis buffer (Gibco). The cells were then stained with live/dead stain as instructed in the kit (Molecular Probes). Before staining cells with specific antibodies, they were incubated with Fc receptor (FcR) blocking reagent (Miltenyi Biotec, USA) for 10 min at 4°C. Finally, the cells were stained with C38‐PE (Miltenyi Biotec, 1/66), CD31‐VioBright 515 (Miltenyi Biotec, 1/50), and CD45‐BV785 (Biolegend, 1/66) for 1 h at 4°C. The cells were then centrifuged at 350 *g* for 10 min at 4°C. The cells were fixed using 2% paraformaldehyde for 15 min at room temperature. After centrifugation, the cells were resuspended in cell staining buffer (Biolegend, USA) and flow cytometry was run on LSRII (BD Biosciences). The analysis was performed using FlowJo software (FlowJo LLC). These experiments were performed in blind.

### Troponin I and BNP measurements

Plasma levels of cardiac troponin I and BNP were measured by sandwich immunoassay methods using commercially available electrochemiluminescent detection system, plates, and reagents (V‐PLEX kits, refs K15186C and K 153KFD, Meso Scale Discovery, Gaithersburg, USA) as per the manufacturer’s instructions. Briefly, 20μL of plasma was loaded per well in the Meso Scale Discovery Plate. Plates were analyzed using the SECTOR Imager 2400. These experiments were performed in blind.

### Metabolite measurements

To determine intracellular NAD^+^ levels, approximately 15 mg of tissue was extracted by homogenizing tissue samples in a buffered ethanol solution (75% ethanol/25% HEPES 10 mM, pH 7.1, and 10 µl/mg of tissue) with a tissue homogenizer (Precellys R, Bertin Instruments). Extracts were heated at 80°C for 5 min, chilled on ice, and centrifuged for at 15,000 *g* for 15 min at 4°C. NAD^+^ levels were quantified using an MTT‐formazan recycling assay. Sample extracts were diluted in water to a final volume of 25 µl. After adding 100 µl of reaction buffer (600 mM ethanol, 0.5 mM 3‐(4.5‐dimethylthiazol‐2‐yl)‐2.5‐diphenyltetrazolium bromide (MTT), 2 mM phenazine ethosulfate (PES), 120 mM Bicine (pH 7.8), and yeast alcohol dehydrogenase 0.05 mg/ml (Sigma)), the kinetics of the reaction was assessed by measuring OD at 550 nm every 30 s for 40 min using a TECAN Infinite F500 microplate reader. Sample NAD^+^ concentrations were determined by comparing the slope of the reaction (OD/s) to a range of standard NAD^+^ concentrations, except for Fig 6D and Appendix Fig S2A for which the determinations were made with the method described in Chini *et al* (2020).

Other nucleotides (NAM, NMN, ADPR, and cADPR) were measured by ultra‐performance liquid chromatography (UPLC)–mass spectroscopy (MS) assay. The HPLC was at a flow rate of 0.25 ml/min with 99% buffer A from 0 to 3 min, a linear gradient to 99% buffer A/1% buffer B (100% methanol) from 3 to 20 min, 80% buffer A/20% buffer B from 20 to 21 min, a linear gradient to 30% buffer A/70% buffer B from 21 to 28 min at 0.35 ml/min, 99% buffer A/1% buffer B from 28 to 31 min, and a linear gradient to 99% buffer A from 31 to 37 min at 0.25 ml/min. Concentrations were quantified based on the peak area compared with a standard curve and normalized to protein content in the tissue sample. These experiments were performed in blind.

### Nucleus and mitochondrion isolation (isolated cardiomyocytes and diaphragm)

Immediately after the mouse was euthanized, cardiomyocytes were isolated from the whole heart (see the cardiomyocyte isolation for details) and the diaphragm was retrieved. Intact cardiomyocytes were counted using a Malassez counting chamber, and the diaphragm was weighed, in order to normalize the results per million cells for the cardiomyocytes of per milligram of tissue for the diaphragm.

Diaphragm was minced on frozen ceramic with a razor blade. Then, the tissue (or cardiomyocytes) was gently homogenized on ice in about 4 µl/mg of isolation buffer solution 1 (225 mM mannitol, 75 mM sucrose, 0.5% BSA, 0.5 mM EGTA, and 30 mM Tris–HCl, pH 7.4) with precooled Teflon and potter. The homogenate was then centrifuged (4°C) at 750 *g* for 5 min, and the pellet (1) was discarded. The supernatant (1) was centrifuged (4°C) at 1,200 *g* for 5 min. The nucleus pellet (2) was preserved for NAD^+^ dosage (see the relevant section for details). The supernatant (2) was centrifuged (4°C) at 9,000 *g* for 10 min. The supernatant (3) was preserved for NAD^+^ dosage of the cytosolic fraction. The pellet (3) was gently resuspended on ice in 1 ml of isolation buffer solution 2 (225 mM mannitol, 75 mM sucrose, 0.5% BSA, and 30 mM Tris–HCl, pH 7.4). This was then centrifuged (4°C) at 10,000 *g* for 10 min. The supernatant (4) was discarded, and the pellet (4) was gently resuspended on ice in 1 ml of isolation buffer solution 3 (225 mM mannitol, 75 mM sucrose, and 30 mM Tris–HCl, pH 7.4). This was finally centrifuged (4°C) at 10,000 *g* for 10 min, and the pellet (5) (crude mitochondrial fraction) was preserved for NAD^+^ dosage.

### Cardiomyocyte preparation and Ca^2+^ imaging

Cardiomyocyte preparation was obtained as described in Bénitah *et al* (2001). The cells were isolated by enzymatic dissociation according to the method of Langendorff. Ca^2+^ handling was evaluated using a classic protocol of cell electrical stimulation at 2 Hz. Each electrical stimulation gives rise to a cytosolic Ca^2+^ elevation called Ca^2+^ transient, which is visualized by confocal microscopy (Leica SP5) (“line‐scanning mode”) in cardiomyocytes loaded with the calcium‐sensitive dye Fluo‐3 AM excited at 500 nm with a white light laser and emission collected at >510 nm as previously described (Bartoli *et al*, 2020). The cytosolic Ca^2+^ variation was normalized by dividing the peak fluorescence intensity (F) by the average resting fluorescence intensity (F0) after background subtraction. For SR Ca^2+^ load estimation, cardiomyocytes were rapidly perfused with 10 mM caffeine immediately after field stimulation. The amplitude of the caffeine‐evoked [Ca^2+^]i transients was used to assess the SR Ca^2+^ load. Fractional SR release was measured by normalizing the steady state of [Ca^2+^]i transients (peak F/F0) to that of caffeine‐evoked [Ca^2+^]i transients. Post‐rest potentiation was calculated by normalizing the first [Ca^2+^]i transients (peak F/F0) after a period of rest to reach the steady‐state [Ca^2+^]i transients. [Ca^2+^]_i_ transient properties and the amplitude (maximum F/F0) were calculated by using the IDL software with a homemade program.

### Human myotubes and Ca^2+^ imaging

Myotubes were prepared from a skeletal human cell line provided by AFM bank of tissue for research as previously described (Vianello *et al*, 2014). In detail, the cell lines (DMD 6311 and healthy CHQ) were provided by Drs. Vincent Mouly (Hôpital Pitié‐Salpêtrière, Paris, France) and Francesco Muntoni (Centre for Neuromuscular Disease, London, UK). Differentiated myotubes were treated for 24–48 h by 10 or 50 µg/ml of SAR 650984 (Sanofi, isatuximab is the international non‐proprietary name, not a registered trademark). The cultured cells were washed twice in a Krebs buffer solution (145 mM NaCl, 5 mM KCl, 1 mM CaCl_2_, 1 mM MgCl_2_, 5.6 mM glucose, and 10 mM HEPES, pH 7.4) and then loaded with Fluo‐4 AM (4 µM; Thermo Fisher Molecular Probes^TM^, USA) for 45 min at 37°C. The cells were maintained in 1 ml/dish of the same buffer for imaging experiments at room temperature. Changes in cytosolic Ca^2+^ concentration were recorded for 10 min. Calcium imaging was performed with a confocal microscope (SP2; Leica Microsystems, Nanterre, France) equipped with a 40x objective.

### Masson’s trichrome stain

Cryostat muscle sections (10 µm) were stained with Masson’s trichrome stain to visualize connective tissue and muscle fibers in pink and collagen in blue (Sigma kit HT15; Sigma‐Aldrich) and then observed using a scanner Leica Aperio^®^ AT2 (Leica Microsystems). Collagen (stained in blue) was quantified using the Aperio Image Analysis IHC, Leica.

### Fiber‐type immunofluorescence

10‐µm sections were blocked for 45 min in 10% goat serum diluted in PBS 1X solution and incubated overnight at 4°C with primary antibodies for the following myosin heavy‐chain (MyHC) isoforms: MyHC1 (BA‐F8, 1/100 dilution; Developmental Studies Hybridoma Bank, Iowa City, IA), MyHC2A (SC‐71, 1/100 dilution; Developmental Studies Hybridoma Bank), and laminin (Sigma L9393, 1/200 dilution; Sigma‐Aldrich, St. Louis, MO). Secondary antibodies were then incubated at a 1/2000 dilution, using Alexa Fluor Goat anti‐Mouse IgG2b 647 (for MyHC1), Goat anti‐Mouse IgG1 555 (for MyHC2A), and Goat anti‐Rabbit 594 (for laminin). CD 31 (PECAM‐1, 1/100; Thermo Fisher Scientific, Inc., MA) antibody was incubated with laminin antibody, and Alexa Fluor Goat anti‐Rat 488 and Goat anti‐Rabbit 594 were applied at 1/2000 dilution. All samples were visualized by fluorescence microscopy (Olympus scanR high‐content, Shinjuku, Tokyo, Japan).

Fields within stained cross sections were captured at 10× magnification. Images were captured in a 1024 × 1024 pixels array, with similar acquisition parameters across preparations. The composition of the different types of myofibers was measured in approximately 200 muscle fibers from each muscle in each animal and was analyzed using Fiji v1.52p (NIH Image from National Institutes of Health, USA). The same fibers selected for cross‐sectional area measurements were also used to determine the proportion of fiber types in each muscle. Based on the staining pattern, all skeletal muscles fibers were classified as type I, type IIa, and type IIx and/or IIb.

### Immune cell infiltration and inflammation immunofluorescence

Avidin–biotin complex (ABC) staining method was performed to detect separately F4/80, Ly‐6G/Ly‐6C, CD8a, and IL‐6. Serial sections were fixed in 4% paraformaldehyde. All endogenous biotin, biotin receptors, and avidin binding sites present in tissues were blocked according to the manufacturer's specification standard protocol (VECTASTAIN^®^ ABC Kits, Vector Labs, Burlingame, CA, USA). The sections were blocked for 45 min in 10% goat serum diluted in PBS 1X solution and incubated for 2 h at room temperature with primary antibodies for the following: F4/80 (BM8, 1:100; Thermo Fisher Scientific, Inc., Waltham, MA, USA); Ly‐6G/Ly‐6C Monoclonal Antibody (RB6‐8C5 1/100; Thermo Fisher Scientific, Inc., Waltham, MA, USA); CD8a Monoclonal Antibody (53‐6.7, 1/100; Thermo Fisher Scientific, Inc., Waltham, MA, USA); and IL‐6 Polyclonal Antibody (P620, 1/100; Thermo Fisher Scientific, Inc., Waltham, MA, USA). Biotinylated secondary antibodies were then incubated at a 1/500 dilution, using Goat anti‐Rat IgG (H + L) (for F4/80, Ly‐6G/Ly‐6C, and CD8a) and Goat anti‐Rat IgG (H + L) (for IL‐6) (Thermo Fisher Scientific, Inc., Waltham, MA, USA). To detect biotinylated secondary antibodies, streptavidin (Atto 565‐Streptavidin, 1/1000; Sigma‐Aldrich, St. Louis, MO) was used followed by a staining with DAPI. All samples were visualized by fluorescence microscopy (Olympus scanR High‐Content Screening Station, Shinjuku, Tokyo, Japan). Immune cell infiltration and inflammation immunofluorescence fields within stained cross sections were captured at 20× magnification. F4/80, Ly‐6G/Ly‐6C, CD8a, and IL‐6‐positive cells were counted in a region of interest (ROI, 750 × 750 µm). The number of positive cells was counted when in contact with DAPI using Fiji (NIH Image du National Institutes of Health, USA).

### Embryonic myosin immunofluorescence

Frozen transverse limb and diaphragm sections (10 μm thick) were fixed in methanol for 5 min, washed for 5 min in PBS, and incubated in BSA–saponin for 10 min. Sections were incubated for 2 h at room temperature with primary antibodies: rabbit anti‐Laminin (1/500, L9393, Sigma‐Aldrich) and mouse myosin heavy chain (embryonic) (1/2, F1.652, Developmental Studies Hybridoma Bank). After 2 × 5 min washes, sections were incubated with secondary antibodies for 1 h at room temperature: Goat anti‐Rabbit IgG (H + L) (1/200, A11008, Invitrogen) and Cy3‐conjugated AffiniPure F(ab)2 (1/4000, 415‐166‐166, Jackson ImmunoResearch). Nuclei were identified with DAPI. This work has benefited from the facilities and expertise of @BRIDGe (Université Paris‐Saclay, INRAE, AgroParisTech, GABI, 78350 Jouy‐en‐Josas, France) for multiacquisition module used to obtain pictures. Fiber‐type quantitation was carried out by calculating the percentage of positively expressing fibers related to the total area exanimated.

### Toxicity assessment

Samples of liver and kidney were fixed in 10% formalin and embedded in paraffin. Sections of 4 µm were cut and stained with hemalun–eosin–saffron, and a histopathological blind analysis was performed by a board‐certified veterinary pathologist.

### Statistics

All the statistics were performed with GraphPad Prism 8. An outlier analysis was performed using the ROUT method (Q = 1%) on each quantitative data set. For all groups, normality was tested with the Shapiro–Wilk or D’Agostino–Pearson tests. For the comparison between two groups, the variance difference was assessed with a F‐test. When all the groups had a normal distribution and equal variances, two‐tailed Student’s *t*‐test was performed. Otherwise, when the groups had a normal distribution with a difference in variance, two‐tailed Welch’s *t*‐test was performed. When at least one of the groups had a non‐normal distribution, or with *n* ≤ 3, a two‐tailed Mann‐Whitney test was performed. For a comparison between at least three groups, the variance difference was assessed with a Brown‐Forsythe test. When all the groups had a normal distribution and no variance difference, a one‐way ANOVA test was performed. If statistically significant, the one‐way ANOVA was followed by a Fisher LSD’s multiple comparisons test or by Student’s *t*‐test when appropriate. If all the groups had a normal distribution and a variance difference, a one‐way Welch's ANOVA test was used, and when significant, it was followed by a Welch’s *t*‐test for multiple comparisons. Otherwise, if at least one of the groups had a non‐normal distribution, a Kruskal–Wallis test was performed, and if significant, the test was followed by uncorrected Dunn’s test for multiple comparisons, or by the Mann–Whitney tests. For survival rate comparison, a log‐rank (Mantel–Cox), a log‐rank for trend, and the Gehan–Breslow–Wilcoxon tests were performed. For the inhibitor (78c) effect and repeated measures of a chronic treadmill exercise, a two‐way ANOVA test was performed. For contingency table comparison, a chi‐square test was performed. The cumulative distribution comparisons were analyzed with a Kolmogorov–Smirnov distribution test. Values are expressed as mean ± SEM, and the contingency tables are presented in percent.

Significance: *P < 0.05, **P < 0.01, and ***P < 0.001.

## Author contributions

**Antoine de Zélicourt:** Conceptualization; Data curation; Formal analysis; Investigation; Methodology; Writing—original draft; Writing—review & editing. **Abdallah Fayssoil:** Investigation. **Mbarka Dakouane‐Giudicelli:** Investigation. **Isley De Jesus:** Investigation. **Ahmed Karaoui:** Investigation. **Faouzi Zarrouki:** Investigation. **Florence Lefebvre:** Investigation. **Arnaud Mansart:** Investigation. **Jean‐Marie Launay:** Investigation. **Jerome Piquereau:** Formal analysis; Investigation; Writing—review & editing. **Mariana G Tarragó:** Formal analysis; Investigation; Writing—review & editing. **Marcel Bonay:** Resources; Formal analysis; Investigation; Writing—review & editing. **Anne Forand:** Conceptualization; Resources; Data curation; Formal analysis; Supervision; Funding acquisition; Validation; Investigation; Methodology; Writing—original draft; Project administration; Writing—review & editing. **Sophie Moog:** Conceptualization; Resources; Data curation; Formal analysis; Supervision; Funding acquisition; Validation; Investigation; Writing—original draft; Project administration; Writing—review & editing. **France Piétri‐Rouxel:** Resources. **Elise Brisebard:** Investigation. **Claudia C S Chini:** Resources; Formal analysis; Investigation; Writing—review & editing. **Sonu Kashyap:** Investigation. **Matthew J Fogarty:** Investigation. **Gary C Sieck:** Investigation. **Mathias Mericskay:** Investigation. **Eduardo N Chini:** Formal analysis; Investigation; Writing—review & editing. **Ana Maria Gomez:** Formal analysis; Investigation; Writing—review & editing. **José‐Manuel Cancela:** Conceptualization; Resources; Data curation; Formal analysis; Supervision; Funding acquisition; Validation; Investigation; Methodology; Writing—original draft; Project administration; Writing—review & editing. **Sabine de la Porte:** Conceptualization; Resources; Data curation; Formal analysis; Supervision; Funding acquisition; Validation; Investigation; Writing—original draft; Project administration; Writing—review & editing.

## Disclosure and competing interests statement

ENC, J‐MC, SP, and AZ have patents on the use of CD38 inhibitors.

## Supporting information



Appendix

Source Data for Figure 1

Source Data for Figure 2

Source Data for Figure 3

Source Data for Figure 4

Source Data for Figure 5

Source Data for Figure 6

## Data Availability

This study includes no data deposited in external repositories.
