# Supplementary material for: CD38‐NADase is a new major contributor to Duchenne muscular dystrophic phenotype
Source: EMBO Mol Med. 2022 Mar 17;14(5):e12860. doi: 10.15252/emmm.202012860 (PMC9081905; doi:10.15252/emmm.202012860)
Supplement: Supplementary file 1 — Appendix [file EMMM-14-e12860-s005.pdf]

Appendix Figure S1. CD38 expression, NAD^+ metabolites and related enzymes in *mdx/CD38$^{-/-}$* mice.

Appendix Figure S2. NAD^+ metabolism changes in *WT* and *WT/CD38$^{-/-}$* mice.

Appendix Figure S3. Cardiac function of WT and *WT/CD38$^{-/-}$* mice.

Appendix Figure S4. Respiratory function and diaphragm structure of WT and *WT/CD38$^{-/-}$* mice.

Appendix Figure S5. Deletion of CD38 has no effect on cell infiltrations and inflammation markers in diaphragm of WT/*CD3*8$^{-/-}$ mice.

Appendix Figure S6. Effect of CD38 deletion on skeletal muscle structure and function in *WT/CD38$^{-/-}$* mice.

Appendix Figure S7. Pharmacological effects on CD38 inhibitors on WT mice and human myotubes. Cellular distribution of CD38 in *mdx* mice.

Appendix Figure S8. Histological evaluation of systemic toxicity of K-rhein in *mdx* mice treated for 5 weeks (5mg/kg/d). Staining was performed with the saffron eosin hemalun method.

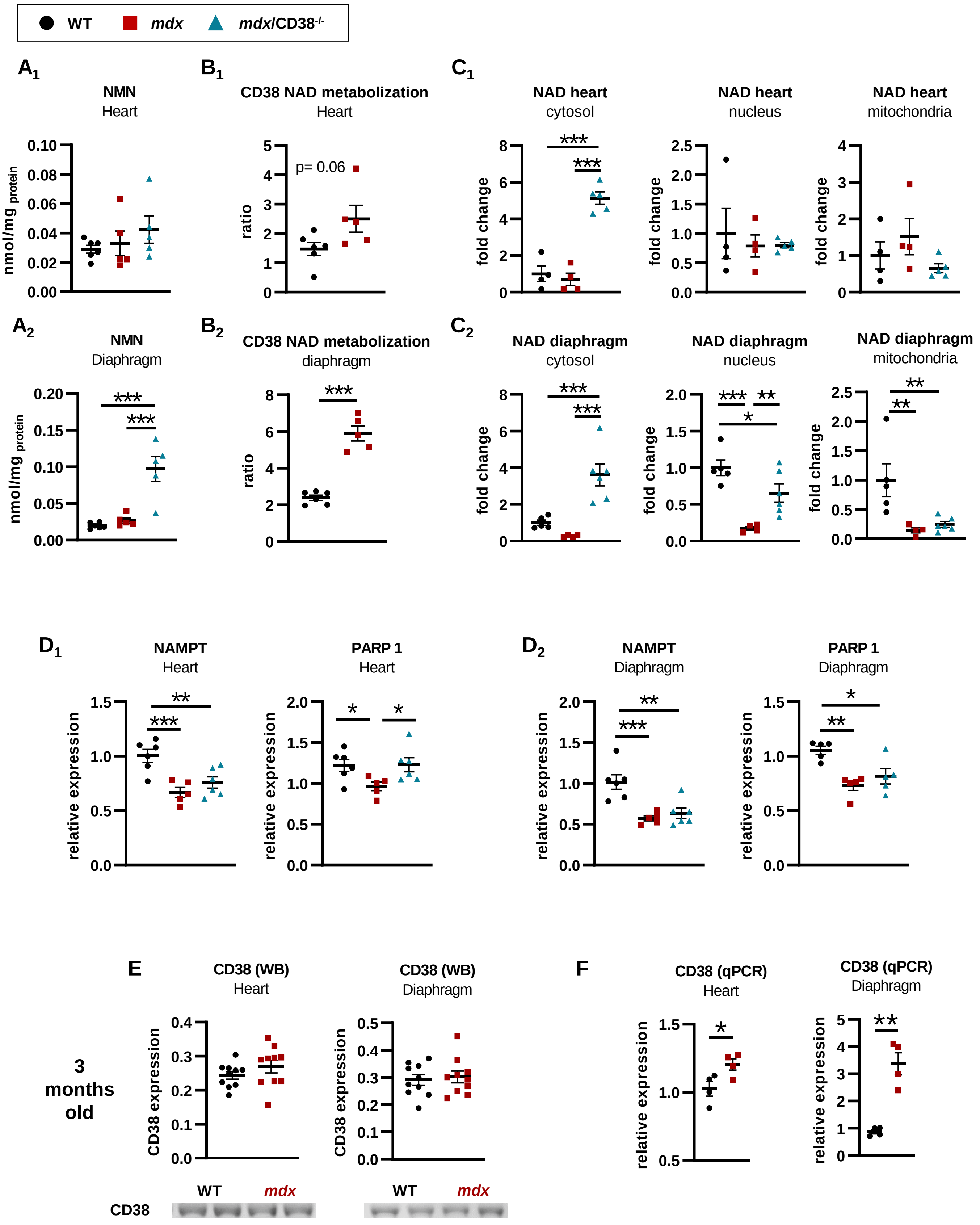

Appendix Figure S1

Appendix Figure S1. CD38 expression, NAD$^+$ metabolites and related enzymes in *mdx/CD38$^{-/-}$* mice.

A1, A2 Dot plots showing nicotinamide mononucleotide (NMN) levels in heart (**A1**) and diaphragm (**A2**) of WT (n=6), *mdx* (n=5) and *mdx/CD38$^{-/-}$* (n=5) mice.

B1, B2 Dot plots showing NAD$^+$ metabolization by CD38 in the heart (**B1**) and diaphragm (**B2**) of WT and *mdx* mice calculated by dividing the NAD$^+$ values obtained respectively in *WT/CD38$^{-/-}$* and in *mdx/CD38$^{-/-}$* mice by the NAD$^+$ values obtained respectively in WT (n=6) and *mdx* mice (n=5).

C1, C2 Subcellular distribution of NAD$^+$ levels in isolated cardiomyocytes (**C1**) and in the diaphragm (**C2**) of WT (n=4 and 5, respectively), *mdx* (n=4) and *mdx/CD38$^{-/-}$* (n=5 and 6, respectively) mice following cell fractionation procedure. The upper panel shows dot plots of cardiomyocyte NAD$^+$ levels in the cytosolic, nuclear and mitochondria fractions. The lower panel shows dot plots of diaphragm NAD$^+$ levels in the cytosolic, nuclear and mitochondria fractions.

D1, D2 Relative mRNA expression, evaluated by qPCR, of nicotinamide phosphoribosyltransferase (NAMPT) and poly-ADP-ribose polymerase 1 (PARP 1) in the heart (**D1**) and the diaphragm (**D2**) of WT (n=6, excepted for PARP 1 in the diaphragm n=5), *mdx* (n=5) and *mdx/CD38$^{-/-}$* (n=6, excepted PARP 1 in the diaphragm n=5) mice.

E Western blot (WB) analysis of CD38 protein expression in the heart and diaphragm of 3-month-old WT and *mdx* mice (n=10 per group). Total protein measurement was used for normalization.

F qPCR analysis of the CD38 mRNA levels in the heart and diaphragm of 3-month-old WT and *mdx* mice (n=4 per group).

Data information: A,B,C,D,F Each dot of the graph represents a mouse and measured in duplicate, graph **E** one value/mouse. After normality and variance comparison tests**,** significance was assessed using: **A$_1$, C$_1$**: ANOVA; **A$_2$**, **C$_1$ (cytosol), C$_2$**, **D$_1$**, **D$_2$** NAMPT: ANOVA followed by Fisher's LSD test; **B$_{1-2}$, E, F** Heart: unpaired Student's t-test; **D$_2$** PARP 1: Kruskal-Wallis followed by Dunn's tests; **F** Diaphragm: unpaired Student's t-test with Welch's correction. Values are expressed as means ± SEM. Significance: *p<0.05, **p<0.01, ***p<0.001.

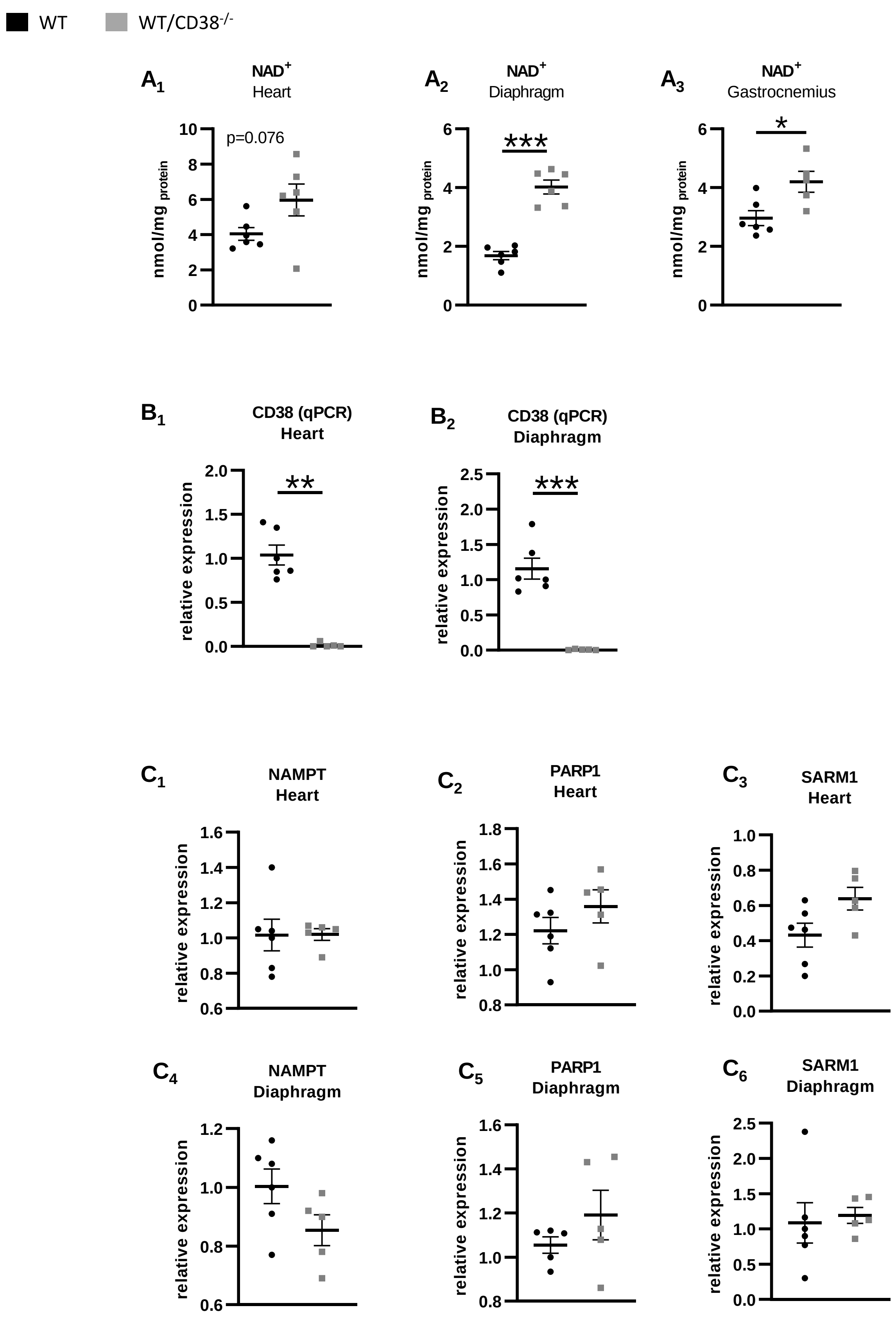

Appendix Figure S2

Appendix Figure S2. NAD$^+$ metabolism changes in *WT* and *WT/CD38$^{-/-}$* mice.

A Measurements of NAD$^+$ levels in WT and *WT/CD38$^{-/-}$* mice: heart and diaphragm (n=6 mice per group) and gastrocnemius (n=6 and n=5 mice, respectively).
B Checking of the absence of CD38 in *WT/CD38$^{-/-}$* mice (n=6 WT and n=5 *WT/CD38$^{-/-}$* mice).
C Relative expression, evaluated by qPCR, of nicotinamide phosphoribosyltransferase (NAMPT), poly-ADP-ribose polymerase 1 (PARP1) and sterile alpha and Toll/interleukin-1 receptor motif-containing 1 (SARM1) in the heart (**C_1, C_2, C_3**) and diaphragm (**C_4, C_5, C_6**) of WT and *WT/CD38$^{-/-}$* mice (n= 6 WT and n=5 *WT/CD38$^{-/-}$* mice per group excepted C_5 n= 5 WT).

Data information: Each dot of the graphs represents a mouse and measured in duplicate. After normality and variance comparison tests**,** significance was assessed using: A, **$C_{2\text{-}6}$**: unpaired Student's t-test, **C_1, B_1**: Mann-Whitney test; B_2: Unpaired t test with Welch's correction. Values are expressed as means ± SEM. Significance: *p<0.05, **p<0.01, ***p<0.001.

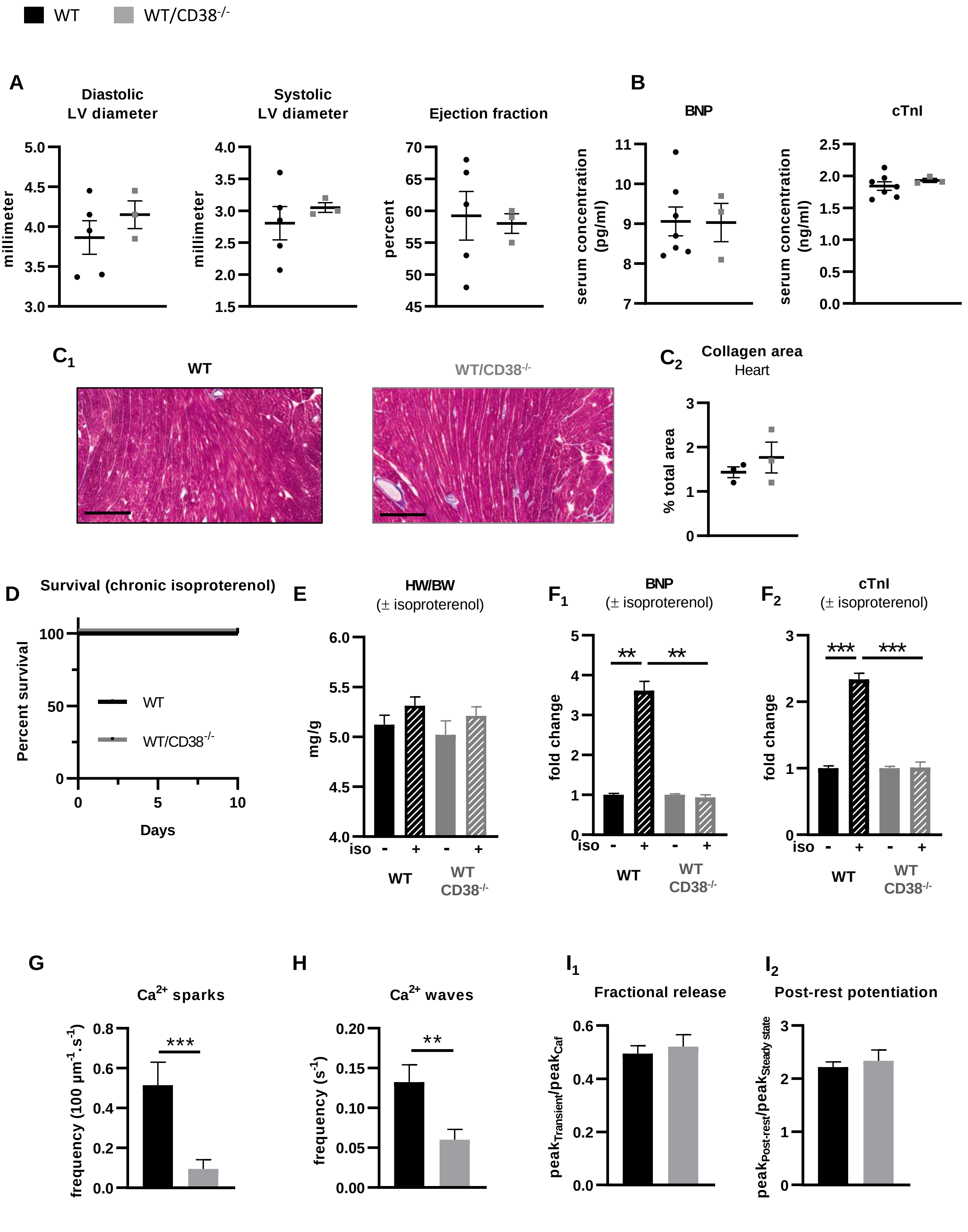

Appendix Figure S3

Appendix Figure S3. Cardiac function of WT and *WT/CD38$^{-/-}$* mice.

A Cardiac function evaluated by echocardiography in WT (n=5) and *WT/CD38$^{-/-}$* (n=3) mice**.** The dot plots show the main cardiac parameters: the left ventricular (LV) diastolic and systolic inner diameters and LV ejection fraction.

B Plasma levels of cardiac stress biomarkers: brain natriuretic peptide (BNP) and cardiac troponin I (cTnI) of WT (n=7) and *WT/CD38$^{-/-}$* (n=3) hearts. No sign of cardiac stress was seen in *WT/CD38$^{-/-}$* mice.

C1, C2 Images showing the absence of fibrosis in heart from WT and *WT/CD38$^{-/-}$* mice, evaluated by Masson's trichrome staining of collagen (blue) on cross-sections (**C1**), . Scale bars: 300 µm. (**C2**) quantification of collagen staining area (% total area) in the heart of WT and *WT/CD38$^{-/-}$* mice (n=3 mice per group).

D Isoproterenol-induced heart failure**.** The Kaplan–Meier curve shows the survival rate of WT (n=6) and *WT/CD38$^{-/-}$* (n=7) mice following isoproterenol subcutaneous injection at 2.5 mg/kg/d for 10 days.

E Histogram showing isoproterenol-induced heart hypertrophy in WT and *WT/CD38$^{-/-}$* mice, expressed as heart weight/body weight ratio (HW/BW). At such low isoproterenol concentration, no significant hypertrophy was observed. NaCl groups: n=6 WT, n=8 *WT/CD38$^{-/-}$*; isoproterenol groups: n=6 WT, n=7 *WT/CD38$^{-/-}$* mice.

F Plasma levels of cardiac stress biomarkers after isoproterenol subcutaneous injection at 2.5 mg/kg/d during 10 days in WT and *WT/CD38$^{-/-}$* mice: isoproterenol-induced an increase of brain natriuretic peptide (BNP)(**F_1**) and of cardiac troponin I (cTnI) (**F_2**) levels in WT mice whereas these markers levels were unchanged in *WT/CD38$^{-/-}$* mice after isoproterenol treatment. NaCl groups: n=5 WT, n=5 *WT/CD38$^{-/-}$*; isoproterenol groups: n=5 WT, n=7 *WT/CD38$^{-/-}$* mice.

G, H Bar graphs showing the averaged Ca^{2+} sparks (**G**) and waves (**H**) frequencies in cardiomyocytes isolated from WT (n=21 cells) and *WT/CD38$^{-/-}$* (n=41 cells) mice.

I Bar graphs showing the fractional release (**I_1**) and the post-rest potentiation (**I_2**) in cardiomyocytes from WT (n=29 cells) and *WT/CD38$^{-/-}$* (n=28 cells) mice. These parameters were calculated as described in the method section.

Data information: Each dot of the graphs represents a mouse. **A,B,F** in duplicate; **D,E** one value/mouse; **C** in triplicate . After normality and variance comparison tests**,** significance was assessed using: **A**, **B, C_2, F_1**, **G**, **H, I_1**: Mann-Whitney test; **D**: Log-rank (Mantel-Cox) and Gehan-Breslow-Wilcoxon tests; **E**: ANOVA; **F_2**: ANOVA followed by unpaired Student's t-tests; **I_2**: unpaired Student's t-test with Welch's correction. Values are expressed as means ± SEM. Significance: *p<0.05, **p<0.01, ***p<0.001.

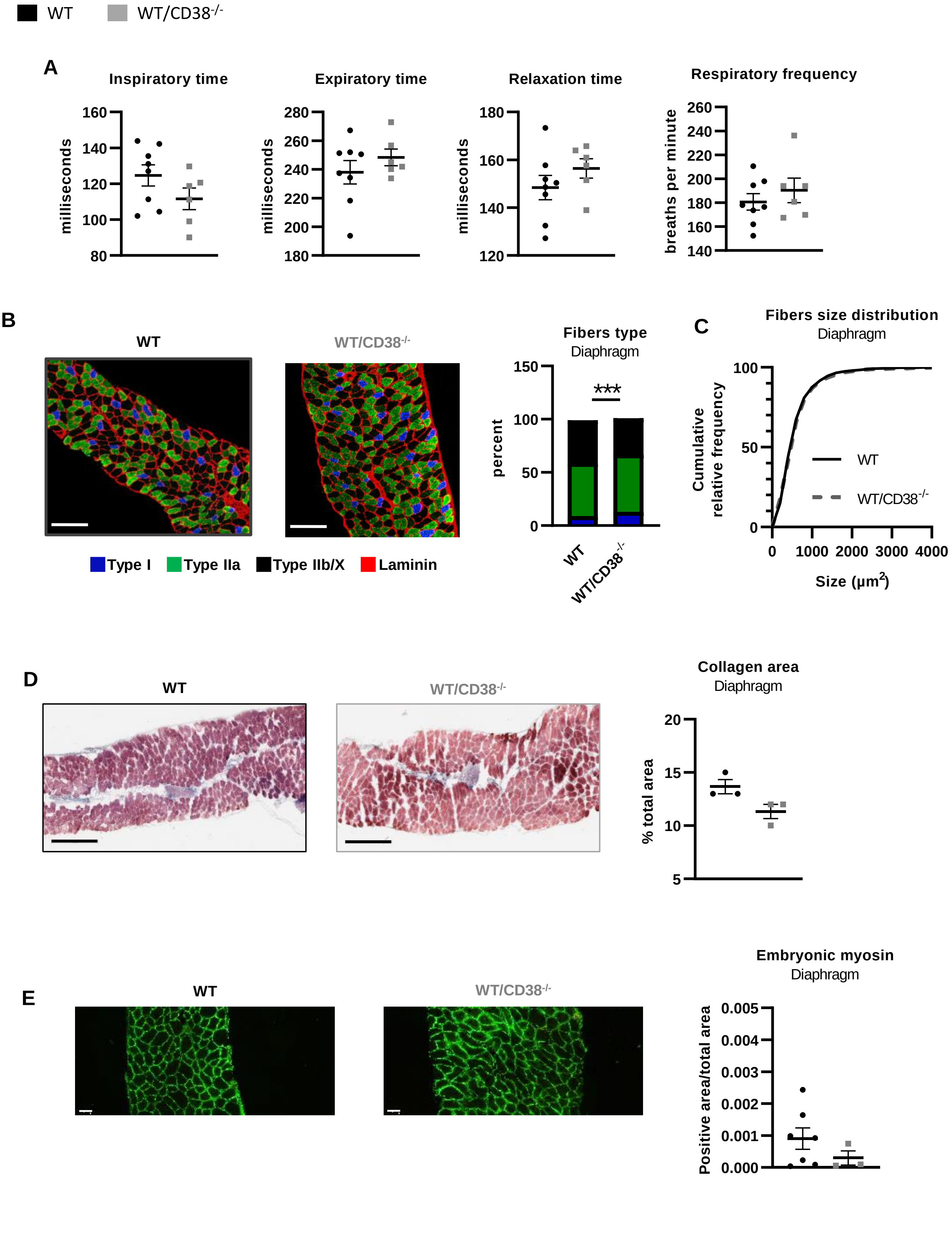

Appendix Figure S4

Appendix Figure S4. Respiratory function and diaphragm structure of WT and *WT/CD38$^{-/-}$* mice.

A Measurement of the ventilatory mechanic parameters by barometric plethysmography: dot plots showing inspiratory (Ti), expiratory (Te) and relaxation times and the respiratory frequency, in WT (n=8) and *WT/CD38$^{-/-}$ mice* (n=6).

B Muscle fiber typology: images showing the immunostaining of slow MyHC (Type I) fiber, fast MyHCs (Type IIa and IIb/X) fibers, along with laminin (red) on transverse cross-sections from diaphragms of WT and *WT/CD38$^{-/-}$ mice*. Scale bars: 100 µm. Histogram: percentage of fiber type (I, IIa, IIb/x) in diaphragm of WT and *WT/CD38$^{-/-}$* mice (n=7, n= 3 mice respectively per group).

C Fibers size distribution in diaphragm of WT and *WT/CD38$^{-/-}$* mice (n=7, n=3 mice respectively per group).

D Left panel: images showing the absence of fibrosis, evaluated by Masson's trichrome staining of collagen (blue), on transverse cross-sections of diaphragm from WT and *WT/CD38$^{-/-}$* mice. Scale bars: 300 µm. Right panel: quantification of collagen staining area (% total area) in the diaphragm of WT and *WT/CD38$^{-/-}$* mice (n=3 per group).

E Left panel: images showing the absence of fibers expressing embryonic myosin evaluated by immunostaining along with laminin (green) on transverse cross-sections from diaphragms of WT and *WT/CD38$^{-/-}$* mice. Scale bars 50 µm. Right panel: quantification of fibers expressing the embryonic myosin in diaphragms of WT (n=3) and *WT/CD38$^{-/-}$* (n=3) mice. The results are expressed by calculating the ratio of positive embryonic myosin fibers area/total area.

Data information: Each dot of the graphs represents a mouse. **A,B,C,E** one value/mouse, **D** in duplicate. After normality and variance comparison tests**,** significance was assessed using: **A**: unpaired Student's t-test; **B**: Chi-square test; **C**: Kolmogorov-Smirnov test, **D**,**E**: Mann-Whitney test. Values are expressed as means ± SEM. Significance: *$p<0.05$, **$p<0.01$, ***$p<0.001$**.**

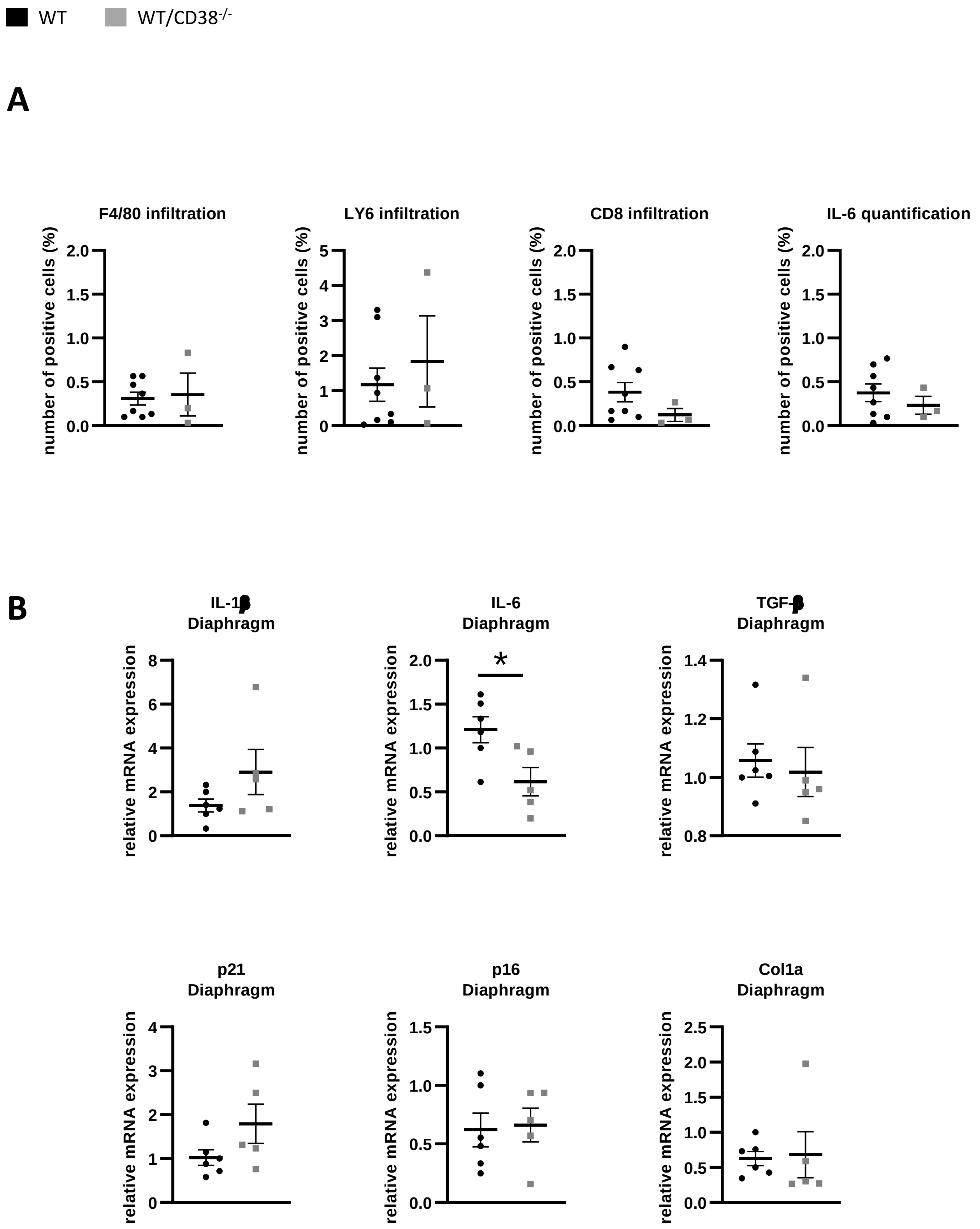

Appendix Figure S5

Appendix Figure S5. Deletion of CD38 has no effect on cell infiltrations and inflammation markers in diaphragm of WT/*CD38*$^{-/-}$ mice.

A Dot plots showing the percentage of various infiltration markers in diaphragms from WT (n=8) and *WT/CD38*$^{-/-}$ (n=3) mice, evaluated by immunostaining of myeloid cells. F4/80 represents macrophages, Ly-6G/6C monocytes, granulocytes and neutrophils, CD8 cytotoxic T-lymphocytes, and IL-6 positive cells.

B Dot plots showing qPCR analysis of mRNA expression levels of cytokine interleukin-1beta (IL-1β) and -6 (IL-6), transforming growth factor-beta (TGF-β), cyclin-dependent kinase inhibitor 1 (p21) and senescence markers (cell-cycle inhibitor p16, INK4a), Col1A1 (Collagen Type I Alpha 1 Chain) in diaphragms of WT (n=6) and *WT/CD38*$^{-/-}$ (n=5) mice.

Data information: **A,B** Each dot of the graphs represents a mouse. **A** one value/mouse; **B** in duplicate. After normality and variance comparison tests**,** significance was assessed using: **A**: Mann-Whitney test; **B**: unpaired Student's t-test excepted for Col1a1 : Mann-Whitney test. Values are expressed as means ± SEM. Significance: *p<0.05, **p<0.01, ***p<0.001**.**

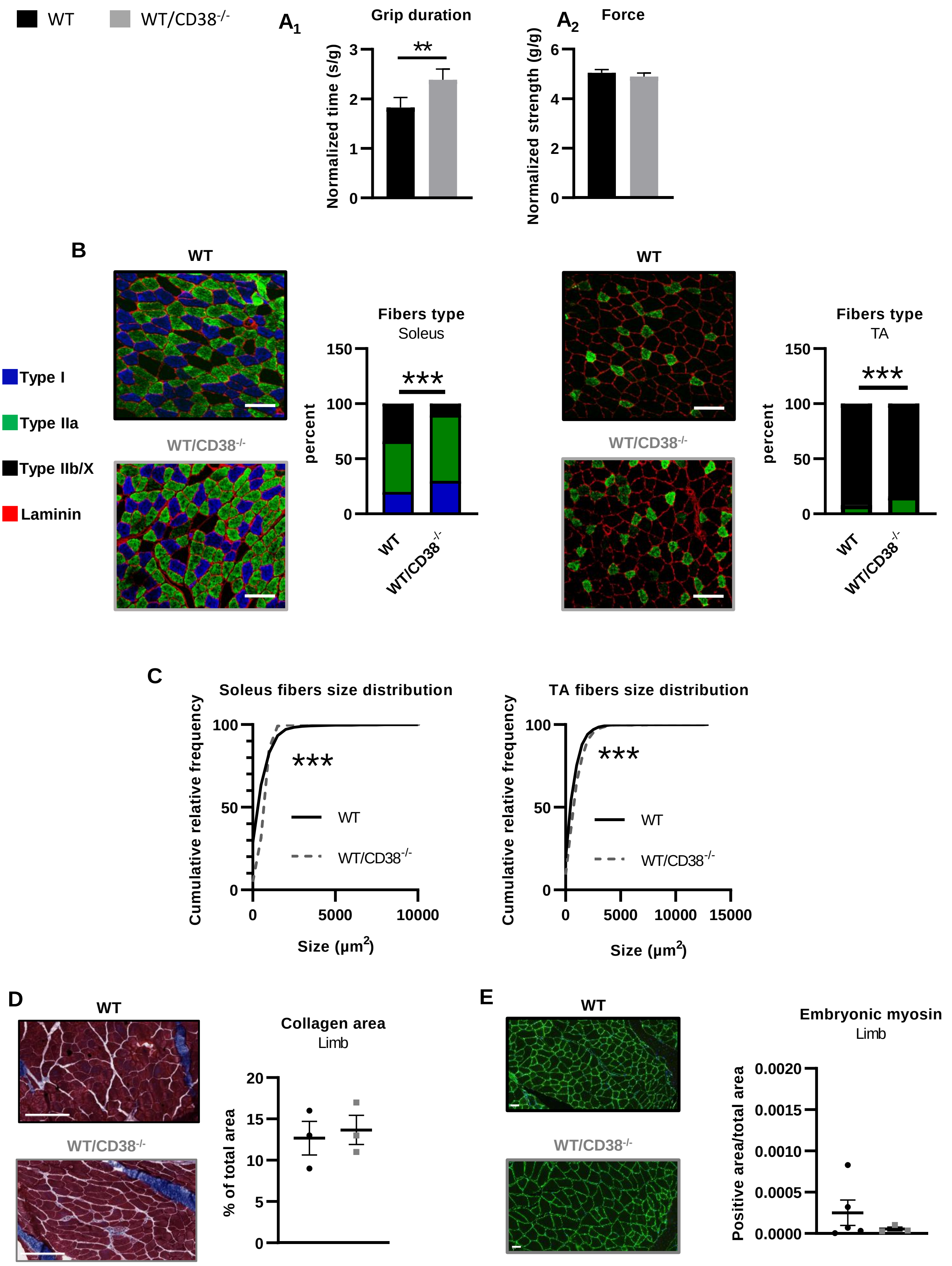

Appendix Figure S6

Appendix Figure S6. Effect of CD38 deletion on skeletal muscle structure and function in *WT/CD38$^{-/-}$* mice.

A Histograms showing the grip duration (latency to fall) (**A_1**) and the limb maximum (max) **(A_2)** force measured in a grip test in WT (n=89) and *WT/CD38$^{-/-}$* (n=67) mice (age: 9-26 months).

B Muscle fiber typology revealed by immunostaining showing the localization of slow MyHC (Type I), and fast MyHCs (Type IIa and IIb/X) fibers, along with laminin (red) on transverse cross-sections from *soleus* and tibialis (TA) of WT and *WT/CD38$^{-/-}$* mice. Scale bars: 100 µm. Histogram showing the percentage of fibers type distribution in the *soleus* and TA of WT and *WT/CD38$^{-/-}$* mice (n=5, n=3, respectively).

C Fibers size distribution in the *soleus* and the TA of WT and *WT/CD38$^{-/-}$* mice (n=5, n=3, respectively).

D Left panel: images showing the absence of fibrosis, evaluated by Masson's trichrome staining of collagen (blue), on limb transverse cross-sections of WT and *WT/CD38$^{-/-}$* mice. Right panel: quantification of collagen staining area in limb of WT and *WT/CD38$^{-/-}$* mice (n=3 per group). Scale bars: 300 µm.

E Left panel: images showing the absence of fibers expressing embryonic myosin evaluated by immunostaining along with laminin (green) on limb transverse cross-sections of WT and *WT/CD38$^{-/-}$* mice. Scale bars: 50 µm. Right panel: area of fibers expressing the embryonic myosin in limb of WT (n=5) and *WT/CD38$^{-/-}$* (n=3) mice.

Data information: Each dot of the graphs represents a mouse. **A_1,B,C,E** one value/mouse, **A_2,D** in triplicate, After normality and variance comparison tests**,** significance was assessed using: **A, D, E**: Mann-Whitney test); **B**: Chi-square test; **C**: Kolmogorov-Smirnov test.Values are expressed as means ± SEM. Significance: *p<0.05, **p<0.01, ***p<0.001.

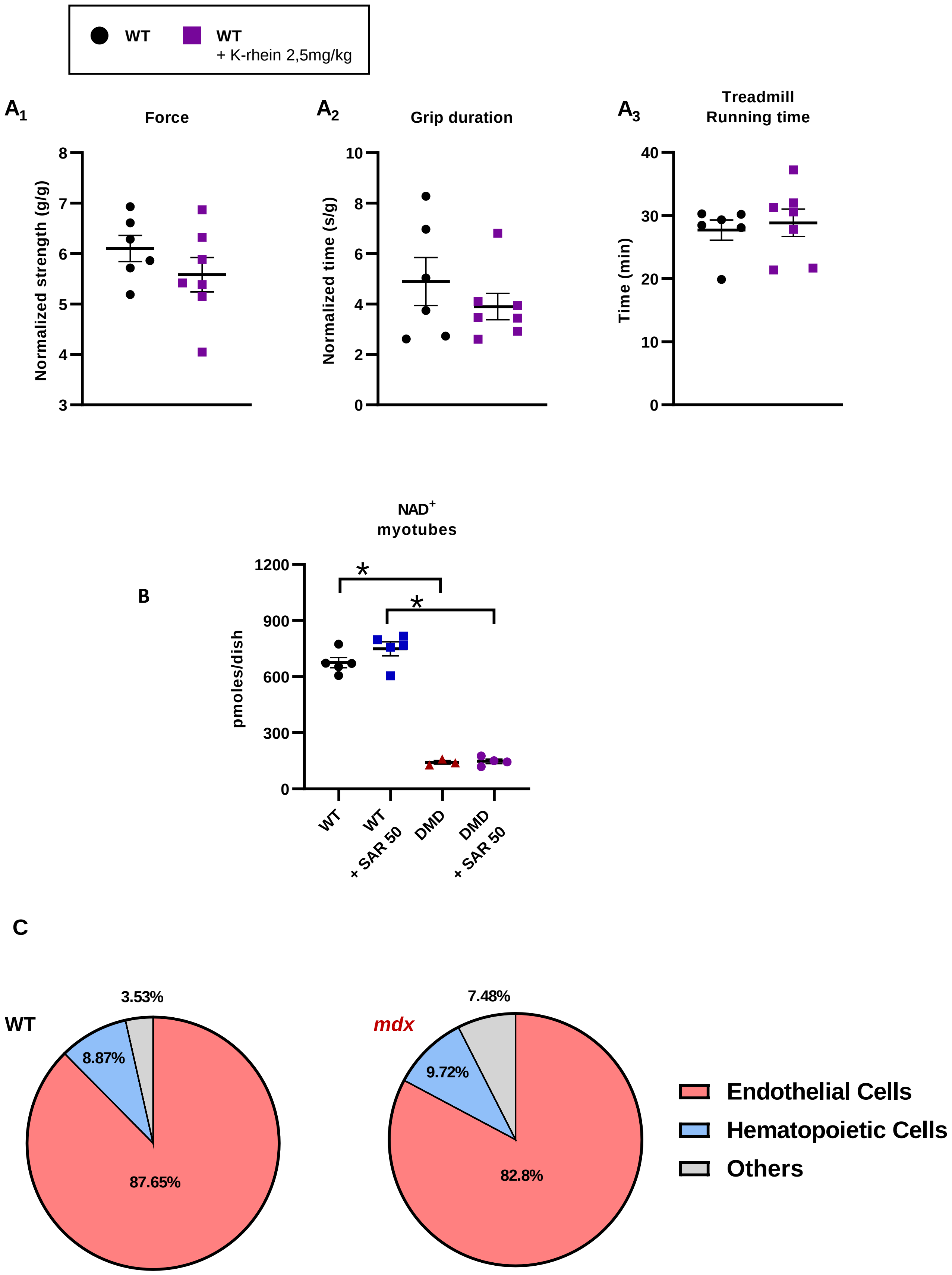

Appendix Figure S7

Appendix Figure S7. Pharmacological effects on CD38 inhibitors on WT mice and human myotubes. Cellular distribution of CD38 in *mdx* mice.

A. Dot plots showing measurement of the grip duration (**A_1**), the force (**A_2**) and the treadmill (**A_3**) performances of WT (n=6) and K-rhein-treated WT (n=7) mice.

B. NAD^+ levels in human healthy myotubes and DMD myotubes untreated and treated by SAR650984 (isatuximab) for 48 hours. Healthy myotubes untreated (n=5 dishes) and treated (n=5 dishes) by SAR650984, DMD myotubes untreated (n=3 dishes) and treated (n=4 dishes) by SAR650984.

C. Pie chart representation of flow cytometry analysis of surface CD38 expression in endothelial (CD31) and hematopoietic cells (CD45) in heart tissue of 16-month-old WT and *mdx* mice (n=3 mice per group).

Data information: Each dot of the graphs represents a mouse. **A_1** in triplicate; **A_2,A_3**, one value/mouse; **C** in duplicate. After normality and variance comparison tests**,** significance was assessed using: **A_1**: unpaired Student's t-test; **A_{2-3}, B, C:** Mann-Whitney test.Values are expressed as means ± SEM. Significance: *$p<0.05$, **$p<0.01$, ***$p<0.001$.

A

Lesion incidence summary		
	mdx	*mdx* + K-rhein
	N=9	N=10
Liver		
mixed inflammatory cell infiltrates, minimal in all mice :		
without cell death	7	8
with cell death	2	2
Kidney		
hyalin casts, minimal	5	2
glomerular changes, minimal	3	1
interstitial fibrosis, multifocal, mild	8	8

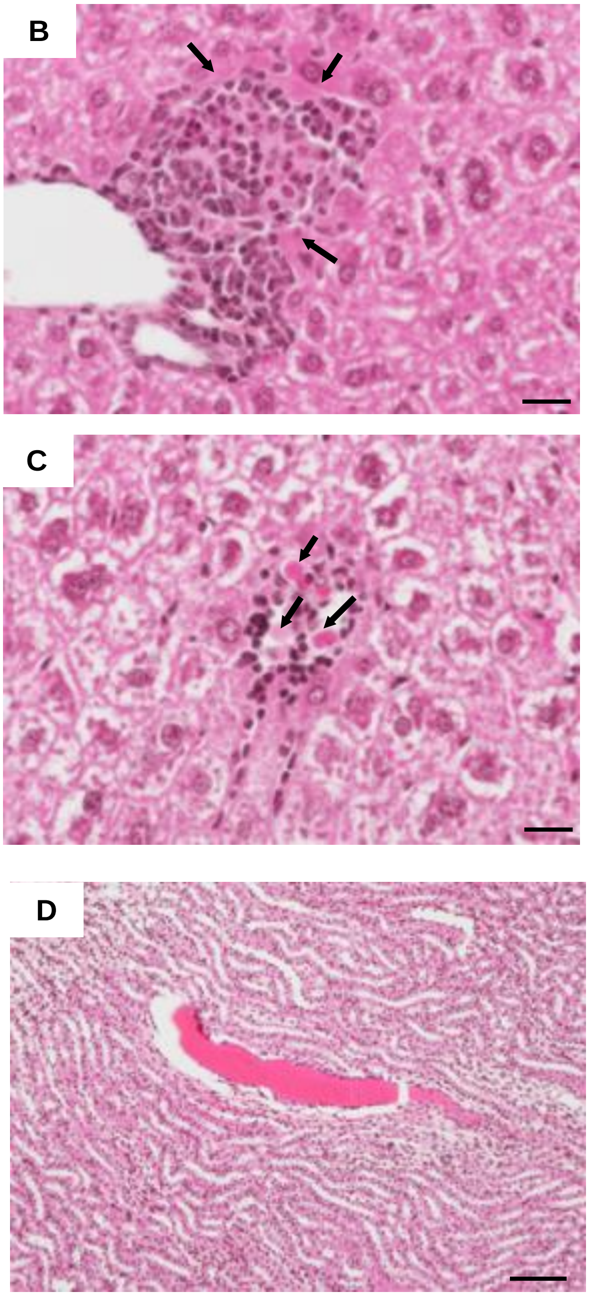

Appendix Figure S8

Appendix Figure S8. Histological evaluation of systemic toxicity of K-rhein in *mdx* mice treated for 5 weeks (5mg/kg/d). Staining was performed with the saffron eosin hemalun method.

A Table of lesion incidence presenting key histopathological findings and their incidence in K-rhein-treated *mdx* mice and control (NaCl 0,9%) *mdx* mice.

B Illustration of some key histopathological findings in the liver and kidney of K-rhein-treated *mdx* mice and untreated *mdx* mice. **(B)** Minimal focal mixed inflammation cell infiltration of a K-rhein treated *mdx* mouse. Small number of similar inflammation cell infiltration foci was observed scattered in the hepatic parenchyma (arrow). Hemalun-eosin-saffron stain. Scale bar: 20 µm. **(C)** Minimal focal mixed inflammation cell infiltration (arrows) in the liver of a untreated *mdx* mouse. Small number of lesions was observed scattered in the hepatic parenchyma. Hemalun-eosin-saffron stain. Scale bar: 20 µm. **(D)** Hyaline cast in the tubular lumen in the renal medulla of a K-rhein treated *mdx* mouse. Hemalun-eosin-saffron stain (Scale bar: 100 µm).
