# Supplementary material for: CD38‐NADase is a new major contributor to Duchenne muscular dystrophic phenotype
Source: EMBO Mol Med. 2022 Mar 17;14(5):e12860. doi: 10.15252/emmm.202012860 (PMC9081905; doi:10.15252/emmm.202012860)
Supplement: Supplementary file 8 [file EMMM-14-e12860-s003.pdf]

EMBO Molecular Medicine

The CD38-NADase is a new major contributor to Duchenne muscular dystrophic phenotype

Antoine de Zelicourt, Abdallah Fayssoil, Mbarka Dakouane-Giudicelli, Isley De Jesus, Karaoui Ahmed, Faouzi Zarrouki, Florence Lefebvre, Arnaud Mansart, Jean-Marie Launay, Jérome Piquereau, Mariana Tarragó, Marcel Bonay, Anne Forand, Sophie Moog, France Piétri-Rouxel, Elise Brisebard, Claudia Chini, Sonu Kashyap, Matthew Fogarty, Gary Sieck, Mathias Mericskay, Eduardo Chini, Ana Gomez, José-Manuel Cancela, and Sabine de la Porte
DOI: 10.15252/emmm.202012860

Corresponding authors: Sabine de la Porte (sabine.delaporte@uvsq.fr) , José-Manuel Cancela (jose-manuel.cancela@u-psud.fr)

Review Timeline:

Submission Date:	1st Jun 20
Editorial Decision:	16th Jun 20
Revision Received:	8th Feb 22
Editorial Decision:	17th Feb 22
Revision Received:	2nd Mar 22
Accepted:	3rd Mar 22

Editor: Zeljko Durdevic

Transaction Report:

(Note: With the exception of the correction of typographical or spelling errors that could be a source of ambiguity, letters and reports are not edited. Depending on transfer agreements, referee reports obtained elsewhere may or may not be included in this compilation. Referee reports are anonymous unless the Referee chooses to sign their reports.)

16th Jun 2020

Dear Dr. de la Porte

Thank you for the submission of your manuscript to EMBO Molecular Medicine. We have now heard back from the three referees who agreed to evaluate your manuscript. As you will see from the reports below, the referees acknowledge the interest of the study. However, they raise serious and partially overlapping concerns that should be addressed in a major revision of the present manuscript. Particular attention should be given to the validation of the findings in a clinically more relevant in vivo model.

Overall it is clear that publication of the manuscript cannot be considered at this stage. I also note that addressing the reviewers concerns in full will be necessary for further considering the manuscript in our journal and this appears to require a lot of additional work and experimentation. I am unsure whether you will be able or willing to address those and return a revised manuscript within the six months deadline. On the other hand, given the potential interest of the findings, I would be willing to consider a revised manuscript with the understanding that the referee concerns must be fully addressed and that acceptance of the manuscript would entail a second round of review.

Please note that EMBO Molecular Medicine encourages a single round of revision only and therefore, acceptance or rejection of the manuscript will depend on the completeness of your responses included in the next, final version of the manuscript. For this reason, and to save you from any frustrations in the end, I would strongly advise against returning an incomplete revision and would also understand your decision if you chose to rather seek rapid publication elsewhere at this stage.

I look forward to receiving your revised manuscript.

Should you find that the requested revisions are not feasible within the constraints outlined here and choose, therefore, to submit your paper elsewhere, we would welcome a message to this effect.

Yours sincerely,

Zeljko Durdevic

Zeljko Durdevic
Editor
EMBO Molecular Medicine

***** Reviewer's comments *****

Referee #1 (Remarks for Author):

This is a potentially interesting and important paper that would significantly move the field forward in the search for a pharmacological treatment for DMD.

Unfortunately, the data are inconsistent, unsatisfactorily presented and flawed at least in part.
As it is now, this manuscript should not be published and extensive revision would be necessary.
Specific comments are listed below.

Introduction:

Authors should briefly describe here rather than in the Discussion the structure and function of CD38 in skeletal muscle.

Results:

In general, time-lapse analysis of calcium spikes both in mouse cardiomyocytes and human myotubes would be much more informative than a single image.

Fig.1 A. Muscles of mdx mice are infiltrated by lymphocytes that express high level of CD38. Authors should demonstrate that the increase of expression is intrinsic to muscle and not consequent to inflammatory infiltrates.

Fig. 2B. Is the transverse section of the heart (left panel) at the same cranio-caudal level of the ventricle? If it was so, then the enlargement of the mdx mouse would not be reversed in the double mutant. This figure is not described at all in the text. I would like to see, even in a supplementary figure, sections of the three hearts at different levels as this would be much more informative.

Suppl. Fig. 1A. It is unclear why a different representation of collagen accumulation is used for the heart (2B) where wt, mdx and mdx/CD38- are shown and for skeletal muscle where wt is not shown. More importantly the quantification hardly reflects the images shown. As known, accumulation of collagen is modest in the limb (TA? It should be specified) but massive in the diaphragm. Images shown reflect this but a 10% difference between the mdx limb, where collagen is barely visible, and the diaphragm, where it occupies more half of the microscopic field, is hard to believe. Also, images clearly show that the diaphragm of the double mutant is still considerably worse than wt and this should be discussed.

Fig. 2C. The experiment is interesting and the results potentially very important. I would have liked to see more controls (wt isoproterenol) and also mdx/CD38- with NaCl only. Then, why the read out is weight rather than EF that would be more informative and has been shown in Fig. 1A?

Text states: "Similar results were obtained concerning the respiratory function (Appendix Fig S1B)." However, S1B shows Azan-Mallory staining of Diaphragm and not any measure of respiratory function.

Fig.3B Exactly the same tests carried out on double mutant mice in 3A are performed on K-Rehin-treated mdx in 3B. It is unclear why data are shown as bars in A and as individual data in B. Much more serious, the same wt animals have about 2s/g as normalised time on the grip test in A and about 5s/g in B. With this variability between controls, all the differences measured are meaningless and so appears to be the grip test as conducted here.

Fig. 3C A different inhibitor 78-c is administered IP twice a day for 6 months for a total of 360 injections. Has an animal welfare committee approved this protocol? Moreover, the two different drugs are not described so that the reader has no idea about their nature, effect and specificity. Also, it is unclear why K-Rehin is administered for a brief period to young mice and 78-C for 6 months to old mice, why the treadmill is tested only in the second case. The distance run is plotted against days and not at different month intervals as one would expect. What are 1, 6 and 7 days, the last of 180? This is not specified. In the second set of experiments wt controls are not shown.

Referee #2 (Remarks for Author):

Comments for the Authors

In the present study, the authors claim that CD38 deletion in mdx mice led to fully restored heart function and structure, as well as skeletal muscle performance improvements, based on their findings regarding the up-regulation of CD38 in mdx mice. Furthermore, they showed that treating mdx mice with two CD38 inhibitors or myotubes from Duchenne muscular dystrophy (DMD) patients with a monoclonal anti-CD38 antibody improved skeletal muscle performance and reduced the pathological and spontaneous RyR Ca2+ activity with elevated NAD+ levels. The authors argue that the beneficial effects of CD38 inhibition is responsible for the fully restored NAD+ levels in muscles. The authors conclude that therapeutic anti-CD38 intervention could be relevant for DMD patients.

Major points

1. The authors demonstrated the up-regulation of CD38 in mdx mice via Western blot analysis. However, it is also important to see the transcriptional activity of CD38 in mdx mice by measuring mRNA levels.
2. Regarding the reduction of pathological Ca2+ activity in cardiomyocytes in mdx/CD38-/- mice, the authors assume CD38 as a known producer of RyR modulators, which is responsible for the excessive Ca2+ activity and oversensitization of the RyRs. However, the authors did not show the product(s) of CD38 from mdx cardiomyocytes. It is important to look at Ca2+ second

messenger potentially responsible for the pathological Ca2+ activity in cardiomyocytes from mdx mice, as the authors anticipated by citing previous papers.
3. In the present study, mice were extensively exposed to two CD38 inhibitors. This raises concern about the toxicity of the chemicals themselves. More concerning would be their potentially detrimental effect on the physiological function of CD38, such as in immune cells, where it functions as a receptor as well as an effector system in Ca2+ signaling.
4. The authors suggest the idea of developing pure inhibitory antibodies for selective anti-CD38 therapeutic intervention. When compared to Isatuximab that was used in the present study, what would the authors consider as a 'pure inhibitory antibody'?
5. The authors used the mdx mice model to evaluate the role of CD38 in DMD. However, unlike human DMD, mdx mice have a significantly longer life span and comparatively lesser muscle destruction, because utrophin is upregulated in skeletal muscle tissue to protect itself. Therefore, many studies regarding the development of drugs for DMD treatment show treatment efficacy in mouse models where mdx and other genes (e.g.,utrophin) are removed together (ref. 1-3; see below). The authors need to include phenotypical analysis by treating an additional DMD mouse model with a CD38 inhibitor to confirm its effects.
6. In this study, the authors have only included a trichrome stain for their pathological examination to confirm the role of CD38. The authors should show the effects of CD38 inhibition through additional pathological findings such as changes in muscle size, muscle type transition, and muscle regeneration.

Ref 1. Stabilization of the cardiac sarcolemma by sarcospan rescues DMD-associated cardiomyopathy. JCI Insight. 2019;4(11):e123855.
Ref 2. Reducing Sarcolipin Expression Mitigates Duchenne Muscular Dystrophy and Associated Cardiomyopathy in Mice. Nat Commun. 2017;20;8(1):1068.
Ref 3. Animal models of Duchenne muscular dystrophy: from basic mechanisms to gene therapy. Dis Model Mech. 2015;8(3):195-213.

Referee #3 (Comments on Novelty/Model System for Author):

In this manuscript, based on evidence from mostly physiological studies in animal and cell models, the authors - de Zélicourt/de la Porte and co-workers - identify CD38-NADase activity as an important actor in the pathobiological mechanisms that underlie DMD. Although the paper is generally well written, the rationale of the study and the design of experiments is fine, and the message of the work is clear and should of value for the pre-clinical and clinical muscle research community, there are a several problematic issues in this study, which need to be addressed further.

Major concerns and points of criticism:
1. The message of this study is not entirely novel, as the importance of NAD+ homeostasis in the etiology and progression of DMD has been addressed in various studies earlier. However, while there are numerous reports on the effects of NAD+ repletion and DMD progression and links with PARP, Nampt and glycolytic v.s. mitochondrial activity (mostly muscle studies!) have been well established, the connection between DMD and CD38 activity has not been studied as intensively. Thus, the findings regarding CD38's role in cardiac pathology as reported here provide new insight. Still, it is important to know that one open access publication with somewhat contradictory findings is currently under review for Skeletal Muscle BMC on Research Square. This is open information on the WEB: Paper is entitled "Complementary NAD+ replacement strategies fail to functionally protect dystrophin-deficient muscle" by David Frederick, Alan V. McDougal, Melisa Semenas, Johanna Vappiani, Andrea Nuzzo, John C. Ulrich, Eugene L. Stewart, Daniel C. Sévin, H. and Fritz Kramer and can be approached via DOI: 10.21203/rs.3.rs-28529/v1 or https://www.researchsquare.com/article/rs-28529/v1.
2. Throughout the study molecular and physiological assays are performed on cell and tissue materials from WT, mdx and mdx/CD38-/- mice, but unfortunately, CD38-/- controls are not simultaneously and parallel analyzed (this remark is pertinent for most studies shown in Fig.1-3 and Suppl.Fig.1). Given the fact that both the mdx and CD38-/- lineages (and probably the WT mice also) have been maintained for prolonged periods on a C57B background in different laboratory environments, it is imperative to have proper controls for all types of background effects. Particularly interesting would be a comparison between the NAD+ levels - and the subcellular distribution patterns thereof - in muscles and hearts of CD38-/- and mdx/CD38-/- mice. As shown in Fig.1A - the expression level of CD38 in individual mice of the WT lineage seems to have a rather constant normalized value for muscle, diaphragm and limb of WT mice but this level varies significantly (even 2-4 fold) between animals that belong to the mdx lineage. This may indeed be a background heterogeneity effect in the breeding population used but needs to be explained.
3. Similarly, throughout this work on muscle tissue more attention should have been focused on the effects of regeneration (i.e. muscle cell heterogeneity, satellite cell content, and thus cell-age heterogeneity in the tissue population) in the different models used. Cell population heterogeneity in the muscle is often a complicating aspect in DMD mouse model studies. See f.e. Duddy et al. Skeletal Muscle (2015) 5:16 -- DOI 10.1186/s13395-015-0041-y for further details.
4. In the experiments shown in Fig.2C-D-E, a study of cardiac effects of isoproterenol on WT mice (to allow comparison between WT, mdx and mdx/CD38-/-) is lacking.
5. To help in the better assessment of specific and also a-specific (fractional) influences of CD38 inhibitors (tri-K salt of 4,5-dihydroxyanthraquinone-2-carboxylic acid (K-rhein) or 78-c) on muscle performance studies as shown in Fig.3B-C-D should also include analyses of WT + NaCL and WT + K-rhein/78c.

6. CD38 in different configurations could have access to both extracellular and intracellular NAD+ and is thus a regulator of NAD+ compartmentalization. To have a better balance between (i) descriptive findings and (ii) mechanistic-clues/-explanations for the "healing" effects of CD38 inhibition or knockout, it would have been particularly interesting to see what effects the mdx (dystrophin-) and/or single or combined CD38 knockout mutation have on the NAD+/NADH levels in the nuclear, cytosolic and mitochondrial compartments of the cell. Studies with NADH autofluorescence monitoring or use of NAD+-fluororeporters in cardiomyocytes or myotubes, with microscopy approaches somewhat similar to those used for the studies shown in Fig. 3 E-F would have been helpful to provide insight in the metabolic effects and form a nice addition to this study. Additionally, to gain insight in effects on differentiation status, integrated stress or cell death, the transcriptional re-programming of cardiomyocytes and myoblasts-myotubes could have been studied.
7. More data are needed to evaluate the cell-based experiments in which the humanized anti-CD38 SAR650984 (isatuximab-irfc antibody was used. Does this Ab provoke both extracellular and intracellular effects? and what were the NAD+ levels in the myotubes studies in Fig.3 F? (and 3E?).
8. In the Materials and Methods more detailed specification of the age of animals for any of the specific tests should be given. Were always individual animals of exactly similar age from the different lineages used for testing?
Minor point: Improvement of use of language by a native English speaker is needed for rephrasing of some text.

Referee #3 (Remarks for Author):

See remarks given for points 1-4 above: - Technical quality (lack of appropriate controls) - novelty (NAD+ has been linked to DMD progression before; CD38 role is novel) - medical impact (difficult to say at this stage of research) - adequacy of model system (the value of mdx mice as model for human DMD has been a topic of dispute for more than 3 decades now) above.

******* Reviewer's comments *******

Referee #1 (Remarks for Author):

This is a potentially interesting and important paper that would significantly move the field forward in the search for a pharmacological treatment for DMD.

We thank the referee for its positive comments

Unfortunately, the data are inconsistent, unsatisfactorily presented and flawed at least in part.
As it is now, this manuscript should not be published and extensive revision would be necessary.
Specific comments are listed below.

We have performed all the requested experiments and we found that our data together with the new experiments are very consistent. Overall, CD38 thus appears to be a major contributor to the DMD.
We have addressed the questions raised point by point in the following paragraph.

Introduction:

Authors should briefly describe here rather than in the Discussion the structure and function of CD38 in skeletal muscle.

We have now added a sentence in the introduction describing the structure and function of CD38 in skeletal muscle.
"*CD38 is a 45kDa single-chain transmembrane enzyme, member of the ADP-ribosyl cyclase family. In muscle and cardiomyocytes, CD38 is both expressed as a type-II and -III transmembrane protein, with its catalytic site exposed to the extracellular space and to the cytosol, respectively (Liu et al, 2017; Lin et al, 2017b; Zhao et al, 2012).*"

Results:

In general, time-lapse analysis of calcium spikes both in mouse cardiomyocytes and human myotubes would be much more informative than a single image.

We have now added the requested time-lapse images of the calcium spikes in human myotubes (DMD patient and healthy subject) and it is now illustrated in the revised manuscript (**Fig. 6G**). As long as the calcium imaging experiment in cardiomyocytes is concerned, the time-lapse images and analysis where already described in the Fig 2 and now better described in **Fig 2G,H.**

Fig.1 A. Muscles of mdx mice are infiltrated by lymphocytes that express high level of CD38. Authors should demonstrate that the increase of expression is intrinsic to muscle and not consequent to inflammatory infiltrates.

We have performed additional experiments on CD38 expression which are now described in the revised manuscript. It is important to note that in our study, CD38 expression in heart and diaphragm of old *mdx* mice was found 2- to 3-fold increase compared with WT mice (**Fig. 1K,L**). We also looked at CD38 expression in heart and diaphragm of young (3 months old) *mdx* mice. No increase in CD38 protein expression was observed in young *mdx* mice

compared with WT mice despite that inflammation is its maximum level at this age (**Appendix Figure S1E,F**) (Porter et al, 2002; Lagrota-Candido et al, 2002; Howard et al, 2021), and the data do not support a role of inflammatory infiltrates in CD38 expression.

Moreover, we also performed flow cytometry experiments on heart from old *mdx* mice and found that looking at CD38 surface expression the majority of CD38 is in endothelial cells in heart tissue (**Appendix Figure S7C).** With regard to hematopoetic cells (which include immune cells), we found no differences between WT and *mdx* mice. In addition, in *mdx* hearts, inflammation is modest, and it is unlikely that a low level of immune cells migration expressing CD38 could explain the difference found in CD38 expression in old *mdx* mice. Nevertheless, and regardless of the higher expression of CD38 in old *mdx* mice and its precise localization in cellular types, our study clearly shows that CD38 deletion or inhibition is beneficial for *mdx* mice irrespective of the age, and was also beneficial in isolated myocytes.

We have now described and discussed these points in the discussion section of the revised manuscript as following:

"*CD38 is an ubiquitous enzyme with NAD^{+} -glycohydrolase activity and its distribution has been extensively studied. CD38 is reported to be expressed in myocytes (Lewis et al, 2012; Guedes et al, 2015; Lee et al, 2015; Park et al, 2018), but also in endothelial cells, fibroblasts and immune cells (Partida-Sanchez et al, 2007; Partida-Sánchez et al, 2001; Boslett et al, 2018; Tarragó et al, 2018). Although we cannot exclude a role for CD38 in non-muscle cells, our results on improved NAD^{+} levels measured in subcellular compartments of isolated cardiomyocytes from mdx/CD38$^{-/-}$ mice; and on Ca^{2+} signalling in isolated cardiomyocytes from mdx/CD38$^{-/-}$ mice; and human DMD myotubes point to an important role of CD38 in muscle functioning. In our study, CD38 expression in young mdx mice is not upregulated but its deletion is clearly beneficial. However, we observed in aged mdx mice higher expression of CD38 in heart, diaphragm and limb. Regarding the mechanisms involved in this high CD38 expression that may occur in aged mdx mice, many studies have shown that reactive oxygen species (Kumasaka et al, 1999; Okabe et al, 2000; Wilson et al, 2001; Zhang et al, 2004) and inflammatory molecules can regulate CD38 expression and/or activity (Bauvois et al, 1999; Deshpande et al, 2003; Kang et al, 2006; Karakasheva et al, 2015). Interestingly, it has been shown that during aging, senescent cells can secrete inflammatory molecules leading to an increase of CD38 expression, which contributes to the NAD^{+} decline linked to aging (Aksoy et al, 2006; Camacho-Pereira et al, 2016; Chini et al, 2019, 2020). Since DMD displays an important oxidative stress and an important inflammatory response (Lawler, 2011; Terrill et al, 2013; Rosenberg et al, 2015), it is therefore likely that in this disease, these molecules play a major role in the up-regulation of CD38 expression that might occur in muscle, endothelial cells or immune cells during aging. We found in heart tissue that the majority of CD38 surface expression measured using flow cytometry is in endothelial cells. However, for the endothelial cells, the number of CD38+ cells has a trend to be lower in mdx mice, with little change observed in hematopoetic cells, or in other cells expressing CD38 (**Appendix Figure S7C**). Thus, it is possible that in DMD, the inflammatory and fibrotic stages may have an influence on the type of cells that express CD38 in the tissue. However, regardless of the specific cells that express CD38, it remains clear that its deletion has brought, directly or indirectly, numerous beneficial effects in different type of muscular tissues or type of isolated myocytes in three different DMD models, namely the mdx and the mdx/utr$^{-/-}$ mice, and the myotubes from DMD patient.*"

Fig. 2B. Is the transverse section of the heart (left panel) at the same cranio-caudal level of the ventricle? If it was so, then the enlargement of the mdx mouse would not be reversed in the double mutant. This figure is not described at all in the text. I would like to see, even in a supplementary figure, sections of the three hearts at different levels as this would be much more informative.

The illustration chosen was misleading since no enlargement of ventricle in *mdx* mouse could be detected using Masson's Trichrome staining of fixed heart sections as illustrated below. We have changed the images in the revised manuscript (**Fig 2C**). The enlargement found at the echocardiography concerns only the inner of the left ventricle of the mdx mouse (ventricular dilatation), and not the external diameter of the heart, which could be mistaken here. We apology for the illustration chosen which was misleading since we found no enlargement in the mdx heart slices (see image below):

WT mice

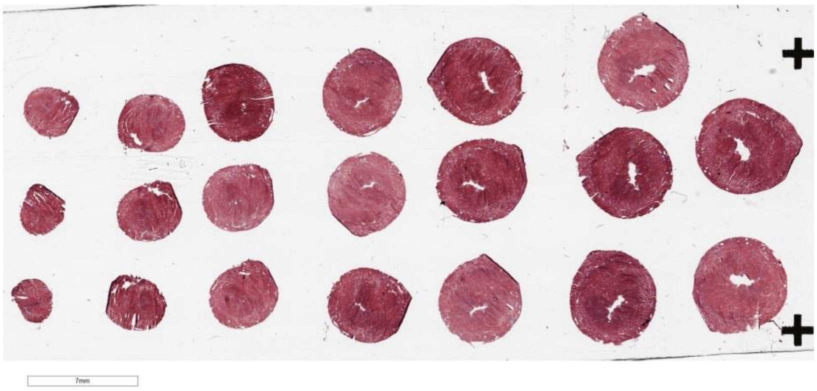

Mdx mice:

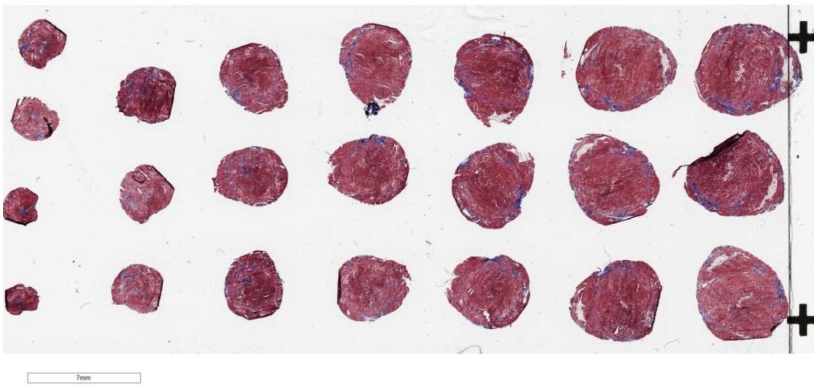

Suppl. Fig. 1A. It is unclear why a different representation of collagen accumulation is used for the heart (2B) where wt, mdx and mdx/CD38- are shown and for skeletal muscle where wt is not shown. More importantly the quantification hardly reflects the images shown. As known, accumulation of collagen is modest in the limb (TA? It should be specified) but massive in the diaphragm. Images shown reflect this but a 10% difference between the mdx limb, where collagen is barely visible, and the diaphragm, where it occupies more half of the microscopic field, is hard to believe. Also,

images clearly show that the diaphragm of the double mutant is still considerably worse than wt and this should be discussed.

- For skeletal muscle section in WT mice : limb is now shown to illustrate the total absence of necrosis in WT mice (**Fig. 5F**). Accumulation of collagen is shown and calculated in the transverse cross section of the limb (including TA, GAS, soleus) (**Fig. 5F**).
- Concerning the values of collagen in the limb and diaphragm. The quantification in the limb has been made on a whole limb section which includes all muscle types, therefore interstitial collagen is highly present between the fibres, fibre groups and between the different muscle types compared with a more limited presence of interstitial collagen in the diaphragm. This constitutive interstitial collagen should not be confused with the collagen resulting from necrosis/fibrosis. Considering only the non-constitutive collagen, the percentage of collagen is in fact 30% in the diaphragm (**Fig. 3D)** and 17 % in the limb of mdx **mice (Fig. 5F**) which represents a reduction of 43% of the collagen staining between the *mdx* diaphragm and *mdx* limb.

Also, images clearly show that the diaphragm of the double mutant is still considerably worse than wt and this should be discussed.

From our images it is clear that CD38 deletion, partially but very significantly, reduced fibrosis in *mdx* diaphragm by 56% and improved the respiratory function. These results should be seen in regard that the diaphragm is the most affected in DMD and it is strongly solicited for the respiratory function (now stated in the revised manuscript). These features likely make the diaphragm a muscle difficult to fully protect in the *mdx* mouse. Nevertheless, this point should not overshadow all the very positive results that we found concerning the diaphragm and the respiratory function in *mdx/CD38*$^{-/-}$ mice. Indeed, we now also performed additional experiments by evaluating the fiber phenotype, muscle regeneration process (embryonic myosin), and numerous inflammatory and senescence markers in the diaphragm (including an analysis of the type 1 collagen mRNA levels) demonstrating an overall improvement in the diaphragm structure (**Fig 4**).

Fig. 2C. The experiment is interesting and the results potentially very important. I would have liked to see more controls (wt ± isoproterenol) and also mdx/CD38- with NaCl only.

As requested by the reviewer, we added the requested groups such as the controls wt ± isoproterenol and *mdx/CD38*$^{-/-}$ with NaCl only. The data are now described and illustrated in the revised manuscript:
- Controls (wt ± isoproterenol) (see **Appendix Figure S3D, E, F** in the revised manuscript): as expected in WT mice with a low dose of isoproterenol, no significant effect of isoproterenol at a dose of 2.5 mg/kg/day was observed on the cardiac hypertrophy of WT mice, but an increase in cardiac stress markers (BNP and c-TnI plasma levels) was found.
- We also added the data on *mdx/CD38*$^{-/-}$ with NaCl only (see the new figure of the revised manuscript **Fig. 2 E and F**) : no effect of NaCl injections on the survival rate, heart weight/body weight ratio or on BNP and c-TnI plasma levels.

Then, why the read out is weight rather than EF that would be more informative and has been shown in Fig. 1A?

In fig 1A, the mice evaluated by echocardiography were 7 months old, an age that permits to detect the early sign of cardiomyopathy linked to DMD. In the isoproterenol-induced heart hypertrophy experiments, we injected younger mice (3 months old) with no apparent cardiac

dysfunction. It has been reported by (Chang et al, 2018) and by our previous experiments that a two-week treatment with isoproterenol would not be sufficient to detect any significant alteration in the EF parameter of the young *mdx* mice and WT mice, and thus we did not perform echocardiographic evaluation of these mice. However, we believe that the parameters we recorded are the one classically recognized and the more informative in this experiment, namely the survival rate, the measure of the heart weight/body weight ratio, histological analyses (Masson's trichrome stain), and measures of plasma cardiac stress markers (BNP and c-TnI) (see **Figures 2D,E,F**).

Text states: "Similar results were obtained concerning the respiratory function (Appendix Figure S1B)." However, S1B shows Azan-Mallory staining of Diaphragm and not any measure of respiratory function.

We apologize for the mistake. The correction has been made and now the data on respiratory function are integrated in the body of the text and described in **Fig. 3A** in the revised manuscript.

Fig.3B Exactly the same tests carried out on double mutant mice in 3A are performed on K-Rehin-treated mdx in 3B. It is unclear why data are shown as bars in A and as individual data in B. Much more serious, the same wt animals have about 2s/g as normalised time on the grip test in A and about 5s/g in B. With this variability between controls, all the differences measured are meaningless and so appears to be the grip test as conducted here.

The bars represent a large number (WT (n=89), *mdx* (n=52) and *mdx/CD38*$^{-/-}$ (n=58) mice) of animals grouped in an age window (age: 9 to 26 months) (now described in **Fig.5C**). While the dot graph representation was chosen due to the smaller number of animals of 3-month-old (WT (n=9), *mdx* (n=7) and K-rhein-treated (n=10) *mdx* mice.) (**Fig.6A)**.
The variability that you pointed out is solely due to the age mice difference between the two experiments that you questioned. As reported in previous studies (Connolly et al, 2001), performances decline with age, including in the WT mice, therefore as expected the value for the grid duration vary with age from 5s/g for the young mice (**Fig.6A)** to decrease to 2s/g for old mice (**Fig 5C**).

Fig. 3C A different inhibitor 78-c is administered IP twice a day for 6 months for a total of 360 injections. Has an animal welfare committee approved this protocol? Moreover, the two different drugs are not described so that the reader has no idea about their nature, effect and specificity. Also, it is unclear why K-Rehin is administered for a brief period to young mice and 78-C for 6 months to old mice, why the treadmill is tested only in the second case. The distance run is plotted against days and not at different month intervals as one would expect. What are 1, 6 and 7 days, the last of 180?

As now added in the M&M section, the protocol for 78-c administration was approved by the IACUC (Institutional animal care and use committee) of the Department of Anesthesiology and Kogod Aging Center, Mayo Clinic, Rochester, Minnesota, USA

In the revised manuscript, we now specify that K-rhein and 78C inhibitors are based on different chemical structure. K-rhein is an uncompetitive antagonist of CD38 derived from flavonoids (Blacher et al, 2015) and 78C is an uncompetitive CD38 antagonist derived from 4-amino-quinolines (Hogan et al, 2019).

The 78c-treated mice were tested during several days starting at the end of the 180 days of treatments. As described in the figure 6E, the *mdx* control mice were less performant with time, in contrary of the 78c-treated mice. The purpose of the protocol was to look at the beneficial effect of 78c on a long-term treatment of 6 months without intermediate tests that could have affected the final results. This was designed to reveal the potential therapeutic effects of this drug by mimicking long-term patient treatments. Additionally, in the 78c protocol, the treadmill was used chronically over few days, to worsen the *mdx* mice phenotype, as described by TREAT-NMD or (Hyzewicz et al, 2015), further mimicking the muscular degeneration seen in DMD patient, which permits to better evaluate the long-term protective effect of the 78c in the *mdx* mouse. This could not be addressed with the short-term protocol used with the short-living and very fragile *mdx/utr-/-* mice treated with K-rhein. Please note that the two different protocols led to the same conclusion, the beneficial effect of CD38 inhibitor treatment in mice.

This is not specified. In the second set of experiments wt controls are not shown.

The effect of 78C on WT controls have been published elsewhere by one of us (Tarragó et al, 2018) and the relevant quotation is present in the manuscript. The previous work described that 78c improved WT mice performance in a treadmill test.

Referee #2 (Remarks for Author):

Comments for the Authors

In the present study, the authors claim that CD38 deletion in mdx mice led to fully restored heart function and structure, as well as skeletal muscle performance improvements, based on their findings regarding the up-regulation of CD38 in mdx mice. Furthermore, they showed that treating mdx mice with two CD38 inhibitors or myotubes from Duchenne muscular dystrophy (DMD) patients with a monoclonal anti-CD38 antibody improved skeletal muscle performance and reduced the pathological and spontaneous RyR Ca^{2+} activity with elevated NAD^+ levels. The authors argue that the beneficial effects of CD38 inhibition is responsible for the fully restored NAD^+ levels in muscles. The authors conclude that therapeutic anti-CD38 intervention could be relevant for DMD patients.

We thank the referee for its positive and constructive comments.

Major points

1. The authors demonstrated the up-regulation of CD38 in mdx mice via Western blot analysis. However, it is also important to see the transcriptional activity of CD38 in mdx mice by measuring mRNA levels.

As requested by the reviewer, we performed mRNA levels analysis of CD38 and it is now described in the revised manuscript as following, Page 3:

"*In parallel, we found a 2- to 3-fold increase in CD38 expression in heart (**Fig. 1K**) and diaphragm (**Fig. 1L**) of old mdx mice compared with WT mice, with no change detected in mRNA levels (**Fig. 1M,N**). In contrast, no difference in CD38 protein expression was observed in young (3-month-old) mdx mice compared with WT mice (**Appendix Figure S1E**) whereas the level of CD38 mRNA was increased (**Appendix Figure S1F**). The discrepancies*

between protein and mRNA levels could be related to the CD38 protein turn over which might be higher in young mdx mice than in the older ones."

2. Regarding the reduction of pathological Ca2+ activity in cardiomyocytes in mdx/CD38-/- mice, the authors assume CD38 as a known producer of RyR modulators, which is responsible for the excessive Ca2+ activity and oversensitization of the RyRs. However, the authors did not show the product(s) of CD38 from mdx cardiomyocytes. It is important to look at Ca2+ second messenger potentially responsible for the pathological Ca2+ activity in cardiomyocytes from mdx mice, as the authors anticipated by citing previous papers.

As requested by the referee, we have measured the endogenous levels of various modulators produced by CD38 when degrading the cellular NAD^+ in the heart and diaphragm tissues. We have first performed experiments in isolated cardiomyocytes and found that cytosolic NAD^+ is consumed by CD38 in these cells (see **Appendix Figure S1C**). Due to the technical limitations of metabolite detection in our assays in isolated cells, we then measured various products of CD38 in the heart and diaphragm tissues. From our data, it appears that the basal level of cyclic ADP-ribose, a RyR modulator produced by CD38 was increased in *mdx*/$CD38^{-/-}$ muscle tissues which could be due to a higher SARM1 activity under the drive of higher NMN levels (Zhao et al, 2019; Essuman et al, 2017) observed in *mdx/CD38*$^{-/-}$ mice (**Appendix Figure S1A**). On the other hand, the ADP-ribose level, the most abundant product of CD38, was dramatically reduced both in heart and diaphragm in *mdx*/$CD38^{-/-}$ mice. This last result suggests that the beneficial effect of CD38 deletion on Ca^{2+} signaling could be linked to ADP-ribose, possibly through its ability to open the TRPM2 channel. In fact, in various cell types including cardiomyocytes, ADP-ribose is known to trigger Ca^{2+} influx through TRPM2 located at the plasma membrane, which could lead to RyR sensitization through Ca^{2+}-induced Ca^{2+}release (Miller et al, 2013; Fliegert et al, 2017; Lee et al, 2018).

These data and the text have been changed accordingly in the revised manuscript in the relevant section (Results, Discussion and Figures).

3. In the present study, mice were extensively exposed to two CD38 inhibitors. This raises concern about the toxicity of the chemicals themselves. More concerning would be their potentially detrimental effect on the physiological function of CD38, such as in immune cells, where it functions as a receptor as well as an effector system in Ca2+ signaling.

We performed the experiments requested by the reviewer in collaboration with a well-recognized French toxicology platform, with a strong experience in DMD models (Larcher *et al*, 2014; Le Guiner *et al*, 2014, 2017) , and the data are now described in the revised manuscript as following:
- Toxicology study (Page 7): "We evaluated the potential toxicological effects of K-rhein. Key histologic features in liver and kidney from control (NaCl 0.9%) and 5-week K-rhein-treated *mdx* mice (9-10 mice per group) were analyzed. No sign of toxicity was observed in the K-rhein treated *mdx* mice compared with untreated *mdx* mice (**Appendix Figure S8**)."

- Concerning the lymphocyte function, we have not looked at it specifically, however in a new set of experiments we observed that the status of inflammatory and fibrosis markers, as well as the overall muscle performance, has been considerably improved when CD38 was deleted or inhibited in *mdx* mice. This shows a clear improvement in their health status. Importantly,

it is well documented that targeting CD38 when highly expressed during aging or in multi-myeloma is beneficial. For instance, CD38 inhibition prevent age-dependent decline in muscle functions and the use of antibodies targeting CD38 has been used successfully in multi-myeloma disease (Baum et al, 2020). Therefore, we believe that our results clearly show that targeting CD38 is beneficial in DMD where NAD^+-dependent consumption is detrimental to the survival of muscles. Finally, we could perform long-lasting experiments with *mdx*/$CD38^{-/-}$ mice as old as 24 months, without noticing premature death that could be due to higher infection rate.

In addition, the levels of inflammatory markers in the diaphragm were not different between WT and $CD38^{-/-}$ mice (**Appendix Figure S5A)**. This indicates that in non-inflammatory conditions the CD38 deletion by itself does not change the inflammatory status of the mice.

Very importantly, we and others have demonstrated that chronic inhibition of CD38 by the small molecule 78c of a blocking antibody AB68 has no obvious detrimental effects in mice (Chini CCS. Nature Metabolism 2020; and Tarrago M et al. Cell Metabolism 2018). .... **CONFIDENTIAL We removed the following figure and its comments related since it is part of a manuscript under evaluation and not yet published.**

4. The authors suggest the idea of developing pure inhibitory antibodies for selective anti-CD38 therapeutic intervention. When compared to Isatuximab that was used in the present study, what would the authors consider as a 'pure inhibitory antibody'?

We thank the referee for the comment. The expression 'pure inhibitory antibody' was unsuitable. We are now giving detailed explanation, Page 10.
"*Importantly, all anti-CD38 antibodies currently used in the clinic for multiple myeloma do not target the catalytic site, but rather an allosteric site, and are potentially cytotoxic through mechanisms such as antibody dependent cell-mediated cytotoxicity (ADCC) or complement dependent cytotoxicity (CDC). Therefore, the development of antibodies directly targeting the CD38 catalytic site with reduced cytotoxic effect should now be a priority (Chini et al, 2018) for further development of such a therapy for DMD.*"

5. The authors used the mdx mice model to evaluate the role of CD38 in DMD. However, unlike human DMD, mdx mice have a significantly longer life span and comparatively lesser muscle destruction, because utrophin is upregulated in skeletal muscle tissue to protect itself. Therefore, many studies regarding the development of drugs for DMD treatment show treatment efficacy in mouse models where mdx and other genes (e.g.,utrophin) are removed together (ref. 1-3; see below). The authors need to include phenotypical analysis by treating an additional DMD mouse model with a CD38 inhibitor to confirm its effects.

We performed the experiments requested by the reviewer by using DKO mice for dystrophin and utrophin (*mdx/utr*$^{-/-}$ mice), and the beneficial effect on *mdx/utr*$^{-/-}$-treated K-rhein mice is now described in the revised manuscript as following in **(Fig. 6**).

"*Furthermore, we investigated the therapeutic benefit of K-rhein in the double knockout utrophin-dystrophin (mdx/utr*$^{-/-}$*) mouse, a model which develops a more severe muscular dystrophic phenotype than the mdx model, with a reduced life expectancy and growth,*

associated with severe motor activity impairments (Grady et al, 1997; Deconinck et al, 1997; Goyenvalle et al, 2015). Newborn four/five-day-old mdx/utr$^{-/-}$ mice were treated 3 days a week with subcutaneous injections of K-rhein (0.6 or 2.5 mg/kg/d) and were evaluated after one month of treatment for their performance on a treadmill, grip and force tests. In the exhaustion treadmill test, we found that mdx/utr$^{-/-}$ mice performance was improved by the K-rhein treatment, with a dose-dependent effect on the distance traveled, the maximum speed reached and the maximum running time ***(Fig. 6B)****. Performances in the grip duration were also improved in mdx/utr$^{-/-}$ mice* ***(Fig. 6C)****.*"

6. In this study, the authors have only included a trichrome stain for their pathological examination to confirm the role of CD38. The authors should show the effects of CD38 inhibition through additional pathological findings such as changes in muscle size, muscle type transition, and muscle regeneration.

As requested by the reviewer, we performed additional experiments by measuring muscle fiber diameter size, fiber type population analysis, and muscle regeneration and it is now described in the result section of the revised manuscript (**Fig. 3 and 5**, respectively) as following:

Concerning the diaphragm (result section page 5, **Fig 3B, C, Appendix Figure S4B**):

Fiber type and size distribution. "*Mature mammals myofibers are classified, according to the myosin heavy chain (MyHC) they express, as slow-contracting aerobic (Type I) and fast-contracting anaerobic fibers (Type IIa, IIb and IIx). Muscle fiber type expression remains plastic under specific conditions such as in respiratory diseases (Polla et al, 2004). To identify the effects of the deletion of CD38 on muscle fibers patterning phenotype, we quantified the number of myofibers of each type, in sections of diaphragms from WT, CD38$^{-/-}$* ***(Appendix Figure S4B)****, mdx and mdx/CD38$^{-/-}$ adult mice* ***(Fig. 3B)****. Compared with WT, mdx diaphragm has more type IIa fibers and a reduced number of type I fibers, which was fully reverted in mdx/CD38$^{-/-}$ diaphragm to the WT mice profile* ***(Fig. 3B)****. Then, fiber size distribution (cross-sectional area) was quantified since it has been reported that, due to the repeated cycles of necrosis and regeneration, mdx mice display a smaller fiber size distribution (Briguet et al, 2004; Dumonceaux et al, 2010; Pertl et al, 2013). Indeed, we found that the mean fiber size distribution was different between WT and mdx mice, with a higher proportion of small diameter fibers in mdx mice, which was restored to a WT profile in mdx/CD38$^{-/-}$ mice* ***(Fig. 3C)****. Collectively, these data indicate that CD38 deletion prevented the alterations of muscle fiber type and size distribution observed in mdx mice diaphragm*"

Concerning the muscle regeneration in the diaphragm (result section page 5, **Fig 3E**):

"*Finally, we performed an immunostaining of positive embryonic myosin fibers, as index of the activation of satellite cells due to the cycles of degeneration/regeneration of mdx muscles. Totally absent in WT mice, the regeneration process observed in mdx mice was strongly reduced in the diaphragm of mdx/CD38$^{-/-}$ mice* ***(Fig. 3E)****. This also reflects the protective effect of CD38 deletion in mdx mice.*"

Concerning the skeletal muscle (result section page 6, **Fig 5D,E,G**),

Fiber type and size distribution: "*We also investigated the relative proportion of each fiber type (I, IIa, IIb/x) in sections of soleus and tibialis anterior (TA). The relative proportion of types IIa and IIb/x fibers was mostly reversed from mdx profile to WT profile in mdx/CD38$^{-/-}$ mice* ***(Fig. 5D)****, with, in addition, an increase of the proportion of slow aerobic type I fibers in*

the soleus, known to be more resistant to degeneration (Webster et al, 1988; Consolino & Brooks, 2004; Selsby et al, 2012; Chalkiadaki et al, 2014) ***(Fig. 5D)****. In soleus, the mean fiber size distribution was not different between WT and mdx mice, but larger size fibers were observed in mdx/CD38$^{-/-}$ mice* ***(Fig. 5E****), which could explain a better muscle function. For TA, no difference was observed concerning the fibers size of the 3 groups (not illustrated).”*

.

Muscle regeneration in the limb (result section page 6 **Fig. 5G)**. “*Similar to diaphragm, muscular degeneration triggers myogenesis in the limb to compensate for the muscle loss. We therefore evaluated myogenesis through embryonic myosin immunostaining: it was totally absent in sections from WT mice limb, clearly present in mdx mice, and strongly reduced in mdx/CD38$^{-/-}$ mice* ***(Fig. 5G)****, which supports again the protective effect of CD38 deletion in mdx mice.*”

Ref 1. Stabilization of the cardiac sarcolemma by sarcospan rescues DMD-associated cardiomyopathy. JCI Insight. 2019;4(11):e123855.
Ref 2. Reducing Sarcolipin Expression Mitigates Duchenne Muscular Dystrophy and Associated Cardiomyopathy in Mice. Nat Commun. 2017;20;8(1):1068.
Ref 3. Animal models of Duchenne muscular dystrophy: from basic mechanisms to gene therapy. Dis Model Mech. 2015;8(3):195-213.

Referee #3 (Comments on Novelty/Model System for Author):

In this manuscript, based on evidence from mostly physiological studies in animal and cell models, the authors - de Zélicourt/de la Porte and co-workers - identify CD38-NADase activity as an important actor in the pathobiological mechanisms that underlie DMD. Although the paper is generally well written, the rationale of the study and the design of experiments is fine, and the message of the work is clear and should of value for the pre-clinical and clinical muscle research community, there are a several problematic issues in this study, which need to be addressed further.

We thank the referee for its comments.

Major concerns and points of criticism:
1. The message of this study is not entirely novel, as the importance of NAD+ homeostasis in the etiology and progression of DMD has been addressed in various studies earlier. However, while there are numerous reports on the effects of NAD+ repletion and DMD progression and links with PARP, Nampt and glycolytic v.s. mitochondrial activity (mostly muscle studies!) have been well established, the connection between DMD and CD38 activity has not been studied as intensively. Thus, the findings regarding CD38's role in cardiac pathology as reported here provide new insight. Still, it is important to know that one open access publication with somewhat contradictory findings is currently under review for Skeletal Muscle BMC on Research Square. This is open information on the WEB: Paper is entitled "Complementary NAD+ replacement strategies fail to functionally protect dystrophin-deficient muscle" by David Frederick, Alan V. McDougal, Melisa Semenas, Johanna Vappiani, Andrea Nuzzo, J
ohn C. Ulrich, Eugene L. Stewart, Daniel C. Sévin, H. and Fritz Kramer and can be approached via DOI: 10.21203/rs.3.rs-28529/v1 or https://www.researchsquare.com/article/rs-28529/v1.

The authors of Frederick *et al* paper aimed to restore NAD^+ levels in *mdx* mice either by using a new CD38 inhibitor (GSK978A) or by using a supplementation with nicotinamide riboside (NR). In their studies, contrary of previous studies reported, both approaches failed to restore the NAD^+ levels in *mdx* skeletal muscles in acute or chronic treatments. Additionally, these treatments (acute and 20 weeks) failed to improve the skeletal muscle structure and function evaluated by eccentric force measurements. I do not believe that the "negative" study published by this group is extremely relevant as a judgement of our own work. First, we have used several state-of-the-art model and techniques, and performed multiple complementary experiments using at least two animal models and two different CD38 small molecule inhibitors. We have analyzed multiple tissues and tissue functions. The study from Frederick has several important aspects that can explain the differences. For example therapeutic effects of a potential treatment in the mdx mouse model should be ideally evaluated through its effect on heart and diaphragm (Fredericks's manuscript did not evaluate these tissues). Also, these team was not able to reproduce data generated by Dr. Johan Auwerx group that demonstrated that Nicotinamide Riboside (NR) supplementation could reverse the dysfunction in animal models of DMD (Science Translational Medicine 8(361):361ra139-361ra139).

Surprisingly, the authors have chosen to treat *mdx* mice with a low dose of GSK978A (3mg/kg/day), despite their inability to demonstrate NAD^+ changes with this dose in *mdx* treated mice. Therefore, it is very difficult to conclude from their study the potential role of CD38. In our hands, using both CD38 deletion in *mdx* mice and pharmacological inhibition, we successfully restored the NAD^+ levels in skeletal muscle, diaphragm and in the heart.

In their paper, Frederick *et al* acknowledge that GSK978A may have not been incorporated in muscle *"Thus, despite broad exposure, the specific pharmacodynamic effects of GSK978A might vary by cell type",* therefore limiting its impact on NAD^+ levels. Intriguingly, GSK978A effect on CD38 was tested on the base-exchange reaction which is a reaction occurring at acidic pH for exchanging nicotinamide from $NADP^+$ with nicotinic acid to form NAADP (Lee, 2012). Clearly, this enzymatic reaction is not the most relevant to use as a screen when looking at NAD^+ hydrolysis, which occurs at neutral pH through the NAD^+ hydrolase activity of CD38 (Lee, 2012). Therefore, it may not be surprising that GSK978A failed to change significantly NAD^+ levels in *mdx* muscles.

Very interestingly, the main result from Frederick *et al* study is actually supportive of our data and demonstrates that "*CD38 inhibition significantly reverts the MDX muscle metabolome to the WT state*". This result indeed, indicate that CD38 inhibition in a DMD model can produce metabolic reprograming of muscle to a more normal state. Thus, we believe that the paper from this group does not invalidate our results for the reasons specified above. Including, not looking at heart, and diaphragm, possible due to dosing of CD38 inhibitor, and also due to the fact that they were not able to reproduce the effect or NR.

2. Throughout the study molecular and physiological assays are performed on cell and tissue materials from WT, mdx and mdx/CD38-/- mice, but unfortunately, CD38-/- controls are not simultaneously and parallel analyzed (this remark is pertinent for most studies shown in Fig.1-3 and Suppl.Fig.1). Given the fact that both the mdx and CD38-/- lineages (and probably the WT mice also) have been maintained for prolonged periods on a C57B background in different laboratory environments, it is imperative to have proper controls for all types of background effects. Particularly interesting would be a comparison between the NAD+ levels - and the subcellular distribution patterns thereof - in muscles and hearts of CD38-/- and mdx/CD38-/- mice. As shown in Fig.1A - the expression level of CD38 in individual mice of the WT lineage seems to have a rather constant normalized value for muscle, diaphragm and limb of WT mice but this level

varies significantly (even 2-4 fold) between animals that belong to the mdx lineage. This may indeed be a background heterogeneity effect in the breeding population used but needs to be explained.

All the mice groups were housed in our animal facility and crossed to generate the *mdx/CD38-/- mice* (see method section). As requested by the reviewer, data on CD38$^{-/-}$ controls are now integrated in the manuscript for the integrality of the experiments done. For example, we found that CD38 deletion in *mdx* mice completely protected the heart from isoproterenol-induced stress, a protection equally observed in WT mice where CD38 was deleted (**Appendix Figure S3D,E,F)**. We have observed no differences in CD38$^{-/-}$ mice in diaphragm and skeletal muscle parameters (structure and function) compared with the WT mice (**Appendix Figures S4, S6).** Deletion of CD38 (CD38$^{-/-}$ mice) had no effect on differentiated muscle patterning phenotype, quantified by the number of myofibers expressing different isoforms in sections of diaphragm muscle from *CD38*$^{-/-}$ mice **(Appendix Figure S4)**. A standardized panel of immunostaining markers F4/80 (macrophage), Ly-6G/6C (monocytes, granulocytes and neutrophils), CD8 (cytotoxic T lymphocytes), and IL-6 positive cells were absent in diaphragm from WT and CD38$^{-/-}$ mice (**Appendix Figure S5A**). We also quantified the relative mRNA expression levels of cytokines such as interleukin-1beta and -6 (IL-1β ; IL-6), cyclin-dependent kinase inhibitor 1 (p21), transforming growth factor-beta (TGF-β), and some specific senescence markers such as cell-cycle inhibitor p16 (INK4a) and finally Col1A1 (Collagen Type I Alpha 1 Chain) for *CD38*$^{-/-}$ mice (**Appendix Figure S5B)** and we observed no differences with the WT mice.

For the western blot analysis of CD38 expression, we checked our data for outliers, (see method section, revised manuscript: "*For each quantitative data set, an outliers research has been performed using the ROUT method (Q=1%)".* We also performed western blot and qPCR of CD38 at two different ages of *mdx* mice (3 months old and 20 months old) and the results are now described in the revised manuscript. We looked at CD38 expression in heart and diaphragm of old *mdx* mice and we found a 2- to 3-fold increase of CD38 protein expression in heart and diaphragm compared with WT mice (**Fig. 1E**). We also looked at CD38 expression in heart and diaphragm of young (3 months old) *mdx* mice. No difference in CD38 protein expression was observed in young *mdx* mice compared with WT mice (**Appendix Figure S1D**). Similar data were obtained in the limb. Therefore, it appears from our additional experiments that CD38 expression changes with age.

3. Similarly, throughout this work on muscle tissue more attention should have been focused on the effects of regeneration (i.e. muscle cell heterogeneity, satellite cell content, and thus cell-age heterogeneity in the tissue population) in the different models used. Cell population heterogeneity in the muscle is often a complicating aspect in DMD mouse model studies. See f.e. Duddy et al. Skeletal Muscle (2015) 5:16 -- DOI 10.1186/s13395-015-0041-y for further details.

As requested by the reviewer, we performed additional experiments by measuring muscle fiber diameter, quantification of fibers type, and muscle regeneration and it is now described in the revised manuscript (**Fig. 3 and 5**, respectively) as following:

Concerning the diaphragm (result section page 5, **Fig 3B, C, Appendix Figure S4B**):
Fiber type and size distribution. "*Mature mammals myofibers are classified, according to the myosin heavy chain (MyHC) they express, as slow-contracting aerobic (Type I) and fast-contracting anaerobic fibers (Type IIa, IIb and IIx). Muscle fiber type expression remains plastic under specific conditions such as in respiratory diseases (Polla et al, 2004). To*

identify the effects of the deletion of CD38 on muscle fibers patterning phenotype, we quantified the number of myofibers of each type, in sections of diaphragms from WT, CD38$^{-/-}$ ***(Appendix Figure S4B)****, mdx and mdx/CD38$^{-/-}$ adult mice (****Fig. 3B)****. Compared with WT, mdx diaphragm has more type IIa fibers and a reduced number of type I fibers, which was fully reverted in mdx/CD38$^{-/-}$ diaphragm to the WT mice profile (****Fig. 3B)****. Then, fiber size distribution (cross-sectional area) was quantified since it has been reported that, due to the repeated cycles of necrosis and regeneration, mdx mice display a smaller fiber size distribution (Briguet et al, 2004; Dumonceaux et al, 2010; Pertl et al, 2013). Indeed, we found that the mean fiber size distribution was different between WT and mdx mice, with a higher proportion of small diameter fibers in mdx mice, which was restored to a WT profile in mdx/CD38$^{-/-}$ mice (****Fig. 3C)****. Collectively, these data indicate that CD38 deletion prevented the alterations of muscle fiber type and size distribution observed in mdx mice diaphragm*"

Concerning the muscle regeneration in the diaphragm (result section page 5, **Fig 3E***)*:
"*Finally, we performed an immunostaining of positive embryonic myosin fibers, as index of the activation of satellite cells due to the cycles of degeneration/regeneration of mdx muscles. Totally absent in WT mice, the regeneration process observed in mdx mice was strongly reduced in the diaphragm of mdx/CD38$^{-/-}$ mice (****Fig. 3E****). This also reflects the protective effect of CD38 deletion in mdx mice."*

<u>Concerning the skeletal muscle </u>(result section page 6, **Fig 5D,E,G***)*,
Fiber type and size distribution: "*We also investigated the relative proportion of each fiber type (I, IIa, IIb/x) in sections of soleus and tibialis anterior (TA). The relative proportion of types IIa and IIb/x fibers was mostly reversed from mdx profile to WT profile in mdx/CD38$^{-/-}$ mice* ***(Fig. 5D)****, with, in addition, an increase of the proportion of slow aerobic type I fibers in the soleus, known to be more resistant to degeneration (Webster et al, 1988; Consolino & Brooks, 2004; Selsby et al, 2012; Chalkiadaki et al, 2014)* ***(Fig. 5D)****. In soleus, the mean fiber size distribution was not different between WT and mdx mice, but larger size fibers were observed in mdx/CD38$^{-/-}$ mice* ***(Fig. 5E****), which could explain a better muscle function. For TA, no difference was observed concerning the fibers size of the 3 groups (not illustrated)."*
.
Muscle regeneration in the limb (result section page 6 **Fig. 5G)**. "*Similar to diaphragm, muscular degeneration triggers myogenesis in the limb to compensate for the muscle loss. We therefore evaluated myogenesis through embryonic myosin immunostaining: it was totally absent in sections from WT mice limb, clearly present in mdx mice, and strongly reduced in mdx/CD38$^{-/-}$ mice* ***(Fig. 5G)****, which supports again the protective effect of CD38 deletion in mdx mice.*"

4. In the experiments shown in Fig.2C-D-E, a study of cardiac effects of isoproterenol on WT mice (to allow comparison between WT, mdx and mdx/CD38-/-) is lacking.

As requested by the reviewer, we have added the control experiment with WT mice treated with isoproterenol. The data are now described and illustrated in the revised manuscript (see **Appendix Figure S3D,E,F**). As expected with the low dose of isoproterenol used (2.5 mg/kg/day), no significant effect was observed in WT mice on the cardiac hypertrophy, but we found an increase in cardiac stress markers (BNP and c-TnI plasma levels). These results agree with the literature showing that *mdx* mice are very sensitive to isoproterenol whereas higher dose of isoproterenol is required to induce heart hypertrophy in WT mice.

5. To help in the better assessment of specific and also a-specific (fractional) influences of CD38 inhibitors (tri-K salt of 4,5-dihydroxyanthraquinone-2-carboxylic acid (K-rhein) or 78-c) on muscle performance studies as shown in Fig.3B-C-D should also include analyses of WT + NaCL and WT + K-rhein/78c.

As requested by the referee, we have added the data obtained in WT mice treated with K-rhein : we observed no effect of K-rhein on the functional performances of WT mice (**Appendix Figure S7A**).
The effect of 78C on WT controls have been published elsewhere by one of us (Tarragó et al, 2018) and now quoted in the revised manuscript. Tarragó et al described that 78c improved WT mice performance in a treadmill test.

6. CD38 in different configurations could have access to both extracellular and intracellular i+ and is thus a regulator of NAD+ compartmentalization. To have a better balance between (i) descriptive findings and (ii) mechanistic-clues/-explanations for the "healing" effects of CD38 inhibition or knockout, it would have been particularly interesting to see what effects the mdx (dystrophin-) and/or single or combined CD38 knockout mutation have on the NAD+/NADH levels in the nuclear, cytosolic and mitochondrial compartments of the cell. Studies with NADH autofluorescence monitoring or use of NAD+-fluororeporters in cardiomyocytes or myotubes, with microscopy approaches somewhat similar to those used for the studies shown in Fig. 3 E-F would have been helpful to provide insight in the metabolic effects and form a nice addition to this study. Additionally, to gain insight in effects on differentiation status, integrated stress or cell death, the transcriptional re-programming of cardiomyocytes and myoblasts-myotubes could have been studied.

The use of NAD^+ fluororeporters would have been valuable, however to set-up this technical approach in our lab would have been very time consuming with some uncertainty on when the requested data will be produced for the revised manuscript. We have therefore chosen to perform subcellular fractionation of cardiomyocytes to measure NAD^+ levels in subcellular compartments. The data are now described in revised manuscript. The NAD^+ levels in the nuclear, cytosolic and mitochondrial compartments of cardiomyocytes have been measured in WT, *mdx* and *mdx/CD38-/-* mice. Our new data performed by measuring subcellular levels of NAD^+ in the cytosol, nucleus and mitochondria indicated that CD38 consumed mostly the cytosolic NAD^+ (**Appendix Figure S1C**).
Concerning the mechanistic clues explaining the healing effects linked to CD38 NAD consumption, we chose not to perform a transcriptional re-programming analysis. This was due to technical difficulties linked to the pandemic and high animal house restrictions. Instead, we performed numerous metabolite measures using various assays (see method section) both in heart and in diaphragm to gain deeper understanding of the mechanisms underlying the beneficial effect of CD38 inhibition in *mdx* mice. In particular, our collaborator Eduardo Chini, a well-recognized expert in CD38 metabolism performed numerous measurements of metabolites relevant to CD38 activity in *mdx/CD38*$^{-/-}$ mice (**Fig 1 and Appendix Figure S1).** Our results show that the beneficial effect of CD38 deletion not only improves NAD^+ levels but also reduces ADPribose levels, the main product of the NAD^+ cleavage by CD38. Knowing that ADPribose act through the TRPM2 channel, we propose that the reduced Ca^{2+} activity observed in mdx/CD38$^{-/-}$ myocytes is likely due to a reduced TRPM2 activity concomitantly with RyRs activity as shown in our cardiomyocyte experiments.

Regarding the additional mechanisms linking CD38 and NAD+ levels, we have given more details in the discussion section of the revised manuscript as following :

"Regarding NAD$^+$ metabolism, CD38 deletion provided a full restoration of the NAD$^+$ levels in all muscle types examined in mdx mice. Our study shows, in a specific pathological context, the importance of CD38 in the excessive cellular NAD$^+$ consumption. The major improvement in the NAD$^+$ levels and the reduced NAM levels observed by inhibiting CD38 is expected to stimulate several beneficial pathways. These pathways could contribute to the positive effects found in the mdx/CD38$^{-/-}$ mice, notably through the activation of sirtuins and its downstream target, the peroxisome-proliferator-activated receptor-gamma coactivator 1alpha (PGC-1α) (Barbosa et al, 2007; Aksoy et al, 2006; Guan et al, 2016; Wang et al, 2018; Tarragó et al, 2018), both known to improve some aspects of the mdx mouse phenotype, including reduced fibrosis and improved treadmill performances (Handschin et al, 2007; Chalkiadaki et al, 2014; Ryu et al, 2016; Capogrosso et al, 2016; Kuno et al, 2018; Sebori et al, 2018), as observed in the present study. Moreover, we also observed that CD38 deletion led to a reversal of the altered muscular fiber phenotype seen in the mdx mouse close to the WT mouse phenotype, notably with an increase of the proportion of slow oxidative fibers in the soleus. This unexpected result might be linked to PGC-1α activity. Indeed, PGC-1α has been proposed to induce, in skeletal muscle cells of the mdx mouse, a switch from glycolytic (fast anaerobic fibers) to oxidative metabolism (slow aerobic fibers), which may contribute to alleviate the dystrophic phenotype of the mdx mouse (Selsby et al, 2012; Ljubicic et al, 2014; Hohenegger et al, 2002; Capogrosso et al, 2016; Lin et al, 2002). »

```
7. More data are needed to evaluate the cell-based experiments in which the
humanized anti-CD38 SAR650984 (isatuximab-irfc antibody was used. Does this
Ab provoke both extracellular and intracellular effects?
```

As requested, we tested the effect of the extemporaneous addition of SAR650984 on the Ca^{2+} waves activity in human DMD myotubes and found no effect (see below). Only a 24 to 48 hours incubation led to a reduction in the Ca^{2+} waves activity as described previously in the manuscript (**Fig 6G,H**), suggesting an intracellular effect of SAR6508984 by internalization of the antibody.

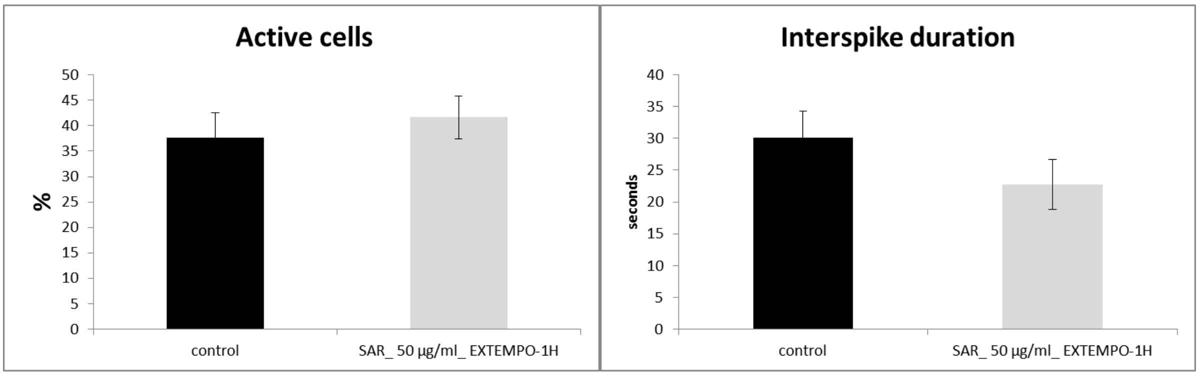

Figure showing the lack of effect of extemporaneous addition of SAR650984 (isatuximab), in human DMD myotubes. Histograms showing the percentage of active cells and the Ca^{2+} wave inter-spike duration (interval between spikes) in myotubes treated extemporaneous with 50 µg/ml of SAR650984 (n=56 cells vs 124 for the untreated DMD myotubes).

```
and what were the NAD+ levels in the myotubes studies in Fig.3 F? (and 3E?)
```

As requested by the reviewer the NAD^+ levels in the myotubes was determined (**Appendix Figure S7B**): we measured a clear decrease in NAD^+ levels in DMD myotubes compared with healthy myotube. However, despite the fact that human myotubes express CD38 (see figure below), SAR650984-treated myotubes (50 µg/ml (**Appendix Figure S7B**)) failed to change the endogenous NAD^+ levels both in healthy and DMD myotubes. This could be due to a peculiar mechanism of action of isotuximab. Indeed, it has been recently reported that CD38 antibody dependent cell-mediated cytotoxicity (ADCC) or complement dependent cytotoxicity (CDC), may act, through allosteric change by targeting, the cyclase activity of CD38 responsible for the cADPRibose production rather than the NAD^+ glycohydrolase activity, which represents the main enzyme reaction catalyzed by CD38 (Baum et al, 2020; Berthelier et al, 1998). Importantly, the Sanofi company has well documented that isotuximab inhibits cADPR production in various cell lines (Baum et al, 2020). Additionally, it appears that one of the main reasons of the lack of effect of isatuximab on NAD in the samples maybe due to the fact that most of the CD38 activity in this cultured cells is likely facing the outside of the cells as an ecto-enzyme. It is also possible the effects are transient and could not captured during our assays.

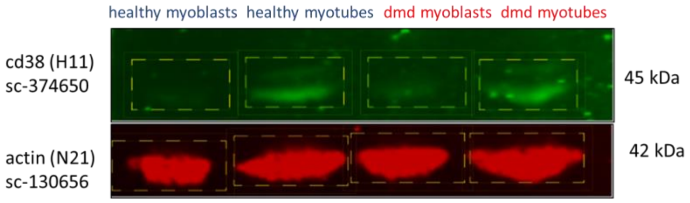

Image showing western blot of CD38 expression in Human DMD differentiated myotubes cell line. Cells were prepared from a skeletal human cell line provided by AFM bank of tissue for research as previously described (Vianello et al, 2014). Briefly, the cells were seeded in dishes coated with Matrigel and grown in a medium consisting of PromoCell skeletal muscle medium with 13% fetal bovine serum. After 5 days, the growth medium was changed to differentiated medium. composed of DMEM and 4.5 mg/ml glucose supplemented 10µg/ml bovine insulin, and 100 µg/ml human transferrin. After another 5 days, the myotubes were analyzed.

8. In the Materials and Methods more detailed specification of the age of animals for any of the specific tests should be given. Were always individual animals of exactly similar age from the different lineages used for testing?

For all the experiments, the age of animals is now stated throughout the text of the revised manuscript.

Minor point: Improvement of use of language by a native English speaker is needed for rephrasing of some text.

A native English speaker has read the text.

Referee #3 (Remarks for Author):

See remarks given for points 1-4 above: - Technical quality (lack of appropriate controls) - novelty (NAD+ has been linked to DMD progression before; CD38 role is novel) - medical impact (difficult to say at this stage of research) - adequacy of model system (the value of mdx mice as model for human DMD has been a topic of dispute for more than 3 decades now) above.

Aksoy P, White TA, Thompson M & Chini EN (2006) Regulation of intracellular levels of NAD: a novel role for CD38. *Biochem Biophys Res Commun* 345: 1386–1392

Barbosa MTP, Soares SM, Novak CM, Sinclair D, Levine JA, Aksoy P & Chini EN (2007) The enzyme CD38 (a NAD glycohydrolase, EC 3.2.2.5) is necessary for the development of diet-induced obesity. *FASEB J* 21: 3629–3639

Baum N, Fliegert R, Bauche A, Hambach J, Menzel S, Haag F, Bannas P & Koch-Nolte F (2020) Daratumumab and Nanobody-Based Heavy Chain Antibodies Inhibit the ADPR Cyclase but not the NAD+ Hydrolase Activity of CD38-Expressing Multiple Myeloma Cells. *Cancers (Basel)* 13: E76

Bauvois B, Durant L, Laboureau J, Barthélémy E, Rouillard D, Boulla G & Deterre P (1999) Upregulation of CD38 gene expression in leukemic B cells by interferon types I and II. *J Interferon Cytokine Res* 19: 1059–1066

Berthelier V, Tixier JM, Muller-Steffner H, Schuber F & Deterre P (1998) Human CD38 is an authentic NAD(P)+ glycohydrolase. *Biochem J* 330 (Pt 3): 1383–1390

Blacher E, Ben Baruch B, Levy A, Geva N, Green KD, Garneau-Tsodikova S, Fridman M & Stein R (2015) Inhibition of glioma progression by a newly discovered CD38 inhibitor. *Int J Cancer* 136: 1422–1433

Boslett J, Hemann C, Christofi FL & Zweier JL (2018) Characterization of CD38 in the major cell types of the heart: endothelial cells highly express CD38 with activation by hypoxia-reoxygenation triggering NAD(P)H depletion. *Am J Physiol, Cell Physiol* 314: C297–C309

Briguet A, Courdier-Fruh I, Foster M, Meier T & Magyar JP (2004) Histological parameters for the quantitative assessment of muscular dystrophy in the mdx-mouse. *Neuromuscul Disord* 14: 675–682

Camacho-Pereira J, Tarragó MG, Chini CCS, Nin V, Escande C, Warner GM, Puranik AS, Schoon RA, Reid JM, Galina A, *et al* (2016) CD38 Dictates Age-Related NAD Decline and Mitochondrial Dysfunction through an SIRT3-Dependent Mechanism. *Cell Metab* 23: 1127–1139

Capogrosso RF, Cozzoli A, Mantuano P, Camerino GM, Massari AM, Sblendorio VT, De Bellis M, Tamma R, Giustino A, Nico B, *et al* (2016) Assessment of resveratrol, apocynin and taurine on mechanical-metabolic uncoupling and oxidative stress in a mouse model of duchenne muscular dystrophy: A comparison with the gold standard, α-methyl prednisolone. *Pharmacol Res* 106: 101–113

Chalkiadaki A, Igarashi M, Nasamu AS, Knezevic J & Guarente L (2014) Muscle-specific SIRT1 gain-of-function increases slow-twitch fibers and ameliorates pathophysiology in a mouse model of duchenne muscular dystrophy. *PLoS Genet* 10: e1004490

Chang SC, Ren S, Rau CD & Wang JJ (2018) Isoproterenol-Induced Heart Failure Mouse Model Using Osmotic Pump Implantation. *Methods Mol Biol* 1816: 207–220

Chini C, Hogan KA, Warner GM, Tarragó MG, Peclat TR, Tchkonia T, Kirkland JL & Chini E (2019) The NADase CD38 is induced by factors secreted from senescent cells providing a potential link between senescence and age-related cellular NAD+ decline. *Biochem Biophys Res Commun* 513: 486–493

Chini CCS, Peclat TR, Warner GM, Kashyap S, Espindola-Netto JM, de Oliveira GC, Gomez LS, Hogan KA, Tarragó MG, Puranik AS, *et al* (2020) CD38 ecto-enzyme in immune cells is induced during aging and regulates NAD+ and NMN levels. *Nat Metab* 2: 1284–1304

Chini EN, Chini CCS, Espindola Netto JM, de Oliveira GC & van Schooten W (2018) The Pharmacology of CD38/NADase: An Emerging Target in Cancer and Diseases of Aging. *Trends Pharmacol Sci*

Connolly AM, Keeling RM, Mehta S, Pestronk A & Sanes JR (2001) Three mouse models of muscular dystrophy: the natural history of strength and fatigue in dystrophin-, dystrophin/utrophin-, and laminin alpha2-deficient mice. *Neuromuscul Disord* 11: 703–12

Consolino CM & Brooks SV (2004) Susceptibility to sarcomere injury induced by single stretches of maximally activated muscles of mdx mice. *J Appl Physiol (1985)* 96: 633–638

Deconinck AE, Rafael JA, Skinner JA, Brown SC, Potter AC, Metzinger L, Watt DJ, Dickson JG, Tinsley JM & Davies KE (1997) Utrophin-dystrophin-deficient mice as a model for Duchenne muscular dystrophy. *Cell* 90: 717–727

Deshpande DA, Walseth TF, Panettieri RA & Kannan MS (2003) CD38/cyclic ADP-ribose-mediated Ca2+ signaling contributes to airway smooth muscle hyper-responsiveness. *FASEB J* 17: 452–454

Dumonceaux J, Marie S, Beley C, Trollet C, Vignaud A, Ferry A, Butler-Browne G & Garcia L (2010) Combination of myostatin pathway interference and dystrophin rescue enhances tetanic and specific force in dystrophic mdx mice. *Mol Ther* 18: 881–887

Essuman K, Summers DW, Sasaki Y, Mao X, DiAntonio A & Milbrandt J (2017) The SARM1 Toll/Interleukin-1 Receptor Domain Possesses Intrinsic NAD+ Cleavage Activity that Promotes Pathological Axonal Degeneration. *Neuron* 93: 1334-1343.e5

Fliegert R, Watt JM, Schöbel A, Rozewitz MD, Moreau C, Kirchberger T, Thomas MP, Sick W, Araujo AC, Harneit A, *et al* (2017) Ligand-induced activation of human TRPM2 requires the terminal ribose of ADPR and involves Arg1433 and Tyr1349. *Biochem J* 474: 2159–2175

Goyenvalle A, Griffith G, Babbs A, El Andaloussi S, Ezzat K, Avril A, Dugovic B, Chaussenot R, Ferry A, Voit T, *et al* (2015) Functional correction in mouse models of muscular dystrophy using exon-skipping tricyclo-DNA oligomers. *Nat Med* 21: 270–275

Grady RM, Teng H, Nichol MC, Cunningham JC, Wilkinson RS & Sanes JR (1997) Skeletal and cardiac myopathies in mice lacking utrophin and dystrophin: a model for Duchenne muscular dystrophy. *Cell* 90: 729–738

Guan X-H, Liu X-H, Hong X, Zhao N, Xiao Y-F, Wang L-F, Tang L, Jiang K, Qian Y-S, Deng K-Y, *et al* (2016) CD38 Deficiency Protects the Heart from Ischemia/Reperfusion Injury through Activating SIRT1/FOXOs-Mediated Antioxidative Stress Pathway. *Oxid Med Cell Longev* 2016: 7410257

Guedes AGP, Deshpande DA, Dileepan M, Walseth TF, Panettieri RA, Subramanian S & Kannan MS (2015) CD38 and airway hyper-responsiveness: studies on human airway smooth muscle cells and mouse models. *Can J Physiol Pharmacol* 93: 145–153

Handschin C, Kobayashi YM, Chin S, Seale P, Campbell KP & Spiegelman BM (2007) PGC-1alpha regulates the neuromuscular junction program and ameliorates Duchenne muscular dystrophy. *Genes Dev* 21: 770–783

Hogan KA, Chini CCS & Chini EN (2019) The Multi-faceted Ecto-enzyme CD38: Roles in Immunomodulation, Cancer, Aging, and Metabolic Diseases. *Front Immunol* 10: 1187

Hohenegger M, Suko J, Gscheidlinger R, Drobny H & Zidar A (2002) Nicotinic acid-adenine dinucleotide phosphate activates the skeletal muscle ryanodine receptor. *Biochem J* 367: 423–431

Howard ZM, Lowe J, Blatnik AJ, Roberts D, Burghes AHM, Bansal SS & Rafael-Fortney JA (2021) Early Inflammation in Muscular Dystrophy Differs between Limb and Respiratory Muscles and Increases with Dystrophic Severity. *The American Journal of Pathology* 191: 730–747

Hyzewicz J, Ruegg UT & Takeda S (2015) Comparison of Experimental Protocols of Physical Exercise for mdx Mice and Duchenne Muscular Dystrophy Patients. *J Neuromuscul Dis* 2: 325–342

Kang B-N, Tirumurugaan KG, Deshpande DA, Amrani Y, Panettieri RA, Walseth TF & Kannan MS (2006) Transcriptional regulation of CD38 expression by tumor necrosis factor-alpha in human airway smooth muscle cells: role of NF-kappaB and sensitivity to glucocorticoids. *FASEB J* 20: 1000–1002

Karakasheva TA, Waldron TJ, Eruslanov E, Kim S-B, Lee J-S, O'Brien S, Hicks PD, Basu D, Singhal S, Malavasi F, *et al* (2015) CD38-Expressing Myeloid-Derived Suppressor Cells Promote Tumor Growth in a Murine Model of Esophageal Cancer. *Cancer Res* 75: 4074–4085

Kumasaka S, Shoji H & Okabe E (1999) Novel mechanisms involved in superoxide anion radical-triggered Ca2+ release from cardiac sarcoplasmic reticulum linked to cyclic ADP-ribose stimulation. *Antioxid Redox Signal* 1: 55–69

Kuno A, Hosoda R, Sebori R, Hayashi T, Sakuragi H, Tanabe M & Horio Y (2018) Resveratrol Ameliorates Mitophagy Disturbance and Improves Cardiac Pathophysiology of Dystrophin-deficient mdx Mice. *Sci Rep* 8: 15555

Lagrota-Candido J, Vasconcellos R, Cavalcanti M, Bozza M, Savino W & Quirico-Santos T (2002) Resolution of skeletal muscle inflammation in mdx dystrophic mouse is accompanied by increased immunoglobulin and interferon-gamma production. *Int J Exp Pathol* 83: 121–132

Larcher T, Lafoux A, Tesson L, Remy S, Thepenier V, François V, Le Guiner C, Goubin H, Dutilleul M, Guigand L, *et al* (2014) Characterization of dystrophin deficient rats: a new model for Duchenne muscular dystrophy. *PLoS ONE* 9: e110371

Lawler JM (2011) Exacerbation of pathology by oxidative stress in respiratory and locomotor muscles with Duchenne muscular dystrophy. *J Physiol (Lond)* 589: 2161–2170

Le Guiner C, Montus M, Servais L, Cherel Y, Francois V, Thibaud J-L, Wary C, Matot B, Larcher T, Guigand L, *et al* (2014) Forelimb treatment in a large cohort of dystrophic dogs supports delivery of a recombinant AAV for exon skipping in Duchenne patients. *Mol Ther* 22: 1923–1935

Le Guiner C, Servais L, Montus M, Larcher T, Fraysse B, Moullec S, Allais M, François V, Dutilleul M, Malerba A, *et al* (2017) Long-term microdystrophin gene therapy is effective in a canine model of Duchenne muscular dystrophy. *Nat Commun* 8: 16105

Lee HC (2012) Cyclic ADP-ribose and nicotinic acid adenine dinucleotide phosphate (NAADP) as messengers for calcium mobilization. *J Biol Chem* 287: 31633–31640

Lee S, Paudel O, Jiang Y, Yang X-R & Sham JSK (2015) CD38 mediates angiotensin II-induced intracellular Ca(2+) release in rat pulmonary arterial smooth muscle cells. *Am J Respir Cell Mol Biol* 52: 332–341

Lee S-H, Kim B-J, Park D-R & Kim U-H (2018) Exercise induces muscle fiber type switching via transient receptor potential melastatin 2-dependent Ca2+ signaling. *J Appl Physiol (1985)* 124: 364–373

Lewis AM, Aley PK, Roomi A, Thomas JM, Masgrau R, Garnham C, Shipman K, Paramore C, Bloor-Young D, Sanders LEL, *et al* (2012) β-Adrenergic receptor signaling increases NAADP and cADPR levels in the heart. *Biochem Biophys Res Commun* 427: 326–329

Lin J, Wu H, Tarr PT, Zhang C-Y, Wu Z, Boss O, Michael LF, Puigserver P, Isotani E, Olson EN, *et al* (2002) Transcriptional co-activator PGC-1 alpha drives the formation of slow-twitch muscle fibres. *Nature* 418: 797–801

Ljubicic V, Burt M, Lunde JA & Jasmin BJ (2014) Resveratrol induces expression of the slow, oxidative phenotype in mdx mouse muscle together with enhanced activity of the SIRT1-PGC-1α axis. *Am J Physiol Cell Physiol* 307: C66-82

Miller BA, Wang J, Hirschler-Laszkiewicz I, Gao E, Song J, Zhang X-Q, Koch WJ, Madesh M, Mallilankaraman K, Gu T, *et al* (2013) The second member of transient receptor potential-melastatin channel family protects hearts from ischemia-reperfusion injury. *Am J Physiol Heart Circ Physiol* 304: H1010-1022

Okabe E, Tsujimoto Y & Kobayashi Y (2000) Calmodulin and cyclic ADP-ribose interaction in Ca2+ signaling related to cardiac sarcoplasmic reticulum: superoxide anion radical-triggered Ca2+ release. *Antioxid Redox Signal* 2: 47–54

Park D-R, Nam T-S, Kim Y-W, Lee S-H & Kim U-H (2018) CD38-cADPR-SERCA Signaling Axis Determines Skeletal Muscle Contractile Force in Response to β-Adrenergic Stimulation. *Cell Physiol Biochem* 46: 2017–2030

Partida-Sánchez S, Cockayne DA, Monard S, Jacobson EL, Oppenheimer N, Garvy B, Kusser K, Goodrich S, Howard M, Harmsen A, *et al* (2001) Cyclic ADP-ribose production by CD38 regulates intracellular calcium release, extracellular calcium influx and chemotaxis in neutrophils and is required for bacterial clearance in vivo. *Nat Med* 7: 1209–1216

Partida-Sanchez S, Gasser A, Fliegert R, Siebrands CC, Dammermann W, Shi G, Mousseau BJ, Sumoza-Toledo A, Bhagat H, Walseth TF, *et al* (2007) Chemotaxis of mouse bone marrow neutrophils and dendritic cells is controlled by adp-ribose, the major product generated by the CD38 enzyme reaction. *J Immunol* 179: 7827–7839

Pertl C, Eblenkamp M, Pertl A, Pfeifer S, Wintermantel E, Lochmüller H, Walter MC, Krause S & Thirion C (2013) A new web-based method for automated analysis of muscle histology. *BMC Musculoskeletal Disorders* 14: 26

Polla B, D'Antona G, Bottinelli R & Reggiani C (2004) Respiratory muscle fibres: specialisation and plasticity. *Thorax* 59: 808–817

Porter JD, Khanna S, Kaminski HJ, Rao JS, Merriam AP, Richmonds CR, Leahy P, Li J, Guo W & Andrade FH (2002) A chronic inflammatory response dominates the skeletal muscle molecular signature in dystrophin-deficient mdx mice. *Hum Mol Genet* 11: 263–272

Rosenberg AS, Puig M, Nagaraju K, Hoffman EP, Villalta SA, Rao VA, Wakefield LM & Woodcock J (2015) Immune-mediated pathology in Duchenne muscular dystrophy. *Sci Transl Med* 7: 299rv4

Ryu D, Zhang H, Ropelle ER, Sorrentino V, Mázala DAG, Mouchiroud L, Marshall PL, Campbell MD, Ali AS, Knowels GM, *et al* (2016) NAD+ repletion improves muscle function in muscular dystrophy and counters global PARylation. *Sci Transl Med* 8: 361ra139

Sebori R, Kuno A, Hosoda R, Hayashi T & Horio Y (2018) Resveratrol Decreases Oxidative Stress by Restoring Mitophagy and Improves the Pathophysiology of Dystrophin-Deficient mdx Mice. *Oxid Med Cell Longev* 2018: 9179270

Selsby JT, Morine KJ, Pendrak K, Barton ER & Sweeney HL (2012) Rescue of dystrophic skeletal muscle by PGC-1α involves a fast to slow fiber type shift in the mdx mouse. *PLoS ONE* 7: e30063

Tarragó MG, Chini CCS, Kanamori KS, Warner GM, Caride A, de Oliveira GC, Rud M, Samani A, Hein KZ, Huang R, *et al* (2018) A Potent and Specific CD38 Inhibitor Ameliorates Age-Related Metabolic Dysfunction by Reversing Tissue NAD+ Decline. *Cell Metab* 27: 1081-1095.e10

Terrill JR, Radley-Crabb HG, Iwasaki T, Lemckert FA, Arthur PG & Grounds MD (2013) Oxidative stress and pathology in muscular dystrophies: focus on protein thiol oxidation and dysferlinopathies. *FEBS J* 280: 4149–4164

Wang L-F, Huang C-C, Xiao Y-F, Guan X-H, Wang X-N, Cao Q, Liu Y, Huang X, Deng L-B, Deng K-Y, *et al* (2018) CD38 Deficiency Protects Heart from High Fat Diet-Induced Oxidative Stress Via Activating Sirt3/FOXO3 Pathway. *Cell Physiol Biochem* 48: 2350–2363

Webster C, Silberstein L, Hays AP & Blau HM (1988) Fast muscle fibers are preferentially affected in Duchenne muscular dystrophy. *Cell* 52: 503–513

Wilson HL, Dipp M, Thomas JM, Lad C, Galione A & Evans AM (2001) Adp-ribosyl cyclase and cyclic ADP-ribose hydrolase act as a redox sensor. a primary role for cyclic ADP-ribose in hypoxic pulmonary vasoconstriction. *J Biol Chem* 276: 11180–11188

Zhang AY, Yi F, Teggatz EG, Zou A-P & Li P-L (2004) Enhanced production and action of cyclic ADP-ribose during oxidative stress in small bovine coronary arterial smooth muscle. *Microvasc Res* 67: 159–167

Zhao ZY, Xie XJ, Li WH, Liu J, Chen Z, Zhang B, Li T, Li SL, Lu JG, Zhang L, *et al* (2019) A Cell-Permeant Mimetic of NMN Activates SARM1 to Produce Cyclic ADP-Ribose and Induce Non-apoptotic Cell Death. *iScience* 15: 452–466

17th Feb 2022

Dear Dr. de la Porte,

Thank you for the submission of your revised manuscript to EMBO Molecular Medicine. I am pleased to inform you that we will be able to accept your manuscript pending the following final amendments:

1) In the main manuscript file, please do the following:
- Make sure that all special characters display well.
- Remove red text color.
- In M&M, provide the antibody dilutions that were used for each antibody.
2) Appendix: Please correct the nomenclature in the table of content and figure legends to "Appendix Figure S1" etc.
3) Conflict of interest: Rename "Conflict of interest" to "Disclosure Statement & Competing Interests". We updated our journal's competing interests policy in January 2022 and request authors to consider both actual and perceived competing interests. Please review the policy https://www.embopress.org/competing-interests and update your competing interests if necessary.
4) Synopsis: Every published paper now includes a 'Synopsis' to further enhance discoverability. Synopses are displayed on the journal webpage and are freely accessible to all readers. They include separate synopsis image and synopsis text.
- Synopsis text: Please provide a short stand first (maximum of 300 characters, including space) as well as 2-5 one sentence bullet points that summarise the paper as a .doc file. Please write the bullet points to summarise the key NEW findings. They should be designed to be complementary to the abstract - i.e. not repeat the same text. We encourage inclusion of key acronyms and quantitative information (maximum of 30 words / bullet point). Please use the passive voice.
- Synopsis image: Please provide a striking image or visual abstract as a high-resolution jpeg file 550 px-wide x (250-400)-px high to illustrate your article.
- Please check your synopsis text and image and submit their final versions with your revised manuscript. Please be aware that in the proof stage minor corrections only are allowed (e.g., typos).
5) Source data: Thank you for providing source data for figure panels. You indicated in your e-mail that you do not wish to include them as supplementary material. Could you please let me know why you prefer not to publish it? We encourage you to include the source data for figure panels that show essential data. Numerical data should be provided as individual .xls or .csv files (including a tab describing the data). For blots or microscopy, uncropped images should be submitted (using a zip archive if multiple images need to be supplied for one panel). Please upload one file per figure (zipp WB source data and .xlsx files were appropriate) for the main Figures and, if provided, zipp source data for all EV Figures as one file. Please check "Author Guidelines" for more information. https://www.embopress.org/page/journal/17574684/authorguide#sourcedata
6) For more information: There is space at the end of each article to list relevant web links for further consultation by our readers. Could you identify some relevant ones and provide such information as well? Some examples are patient associations, relevant databases, OMIM/proteins/genes links, author's websites, etc...
7) As part of the EMBO Publications transparent editorial process initiative (see our Editorial at http://embomolmed.embopress.org/content/2/9/329), EMBO Molecular Medicine will publish online a Review Process File (RPF) to accompany accepted manuscripts. This file will be published in conjunction with your paper and will include the anonymous referee reports, your point-by-point response and all pertinent correspondence relating to the manuscript. Let us know whether you agree with the publication of the RPF and as here, if you want to remove or not any figures from it prior to publication. Please note that the Authors checklist will be published at the end of the RPF.
8) Please provide a point-by-point letter INCLUDING my comments as well as the reviewer's reports and your detailed responses (as Word file).

Please submit your revised manuscript within two weeks. I look forward to reading a new revised version of your manuscript as soon as possible.

Yours sincerely,

Zeljko Durdevic

Zeljko Durdevic
Editor
EMBO Molecular Medicine

*** Instructions to submit your revised manuscript ***

*** PLEASE NOTE *** As part of the EMBO Publications transparent editorial process initiative (see our Editorial at https://www.embopress.org/doi/pdf/10.1002/emmm.201000094), EMBO Molecular Medicine will publish online a Review Process File to accompany accepted manuscripts.

In the event of acceptance, this file will be published in conjunction with your paper and will include the anonymous referee reports, your point-by-point response and all pertinent correspondence relating to the manuscript. If you do NOT want this file to be published, please inform the editorial office at contact@embomolmed.org.

To submit your manuscript, please follow this link:

Link Not Available

When submitting your revised manuscript, please include:

1) a .docx formatted version of the manuscript text (including Figure legends and tables)

2) Separate figure files*

3) supplemental information as Expanded View and/or Appendix. Please carefully check the authors guidelines for formatting Expanded view and Appendix figures and tables at https://www.embopress.org/page/journal/17574684/authorguide#expandedview

4) a letter INCLUDING the reviewer's reports and your detailed responses to their comments (as Word file).

5) The paper explained: EMBO Molecular Medicine articles are accompanied by a summary of the articles to emphasize the major findings in the paper and their medical implications for the non-specialist reader. Please provide a draft summary of your article highlighting
- the medical issue you are addressing,
- the results obtained and
- their clinical impact.
This may be edited to ensure that readers understand the significance and context of the research.
Please refer to any of our published articles for an example.

6) For more information: There is space at the end of each article to list relevant web links for further consultation by our readers. Could you identify some relevant ones and provide such information as well? Some examples are patient associations, relevant databases, OMIM/proteins/genes links, author's websites, etc...

7) Author contributions: the contribution of every author must be detailed in a separate section.

8) EMBO Molecular Medicine now requires a complete author checklist (https://www.embopress.org/page/journal/17574684/authorguide) to be submitted with all revised manuscripts. Please use the checklist as guideline for the sort of information we need WITHIN the manuscript. The checklist should only be filled with page numbers were the information can be found. This is particularly important for animal reporting, antibody dilutions (missing) and exact values and n that should be indicted instead of a range.

9) Every published paper now includes a 'Synopsis' to further enhance discoverability. Synopses are displayed on the journal webpage and are freely accessible to all readers. They include a short stand first (maximum of 300 characters, including space) as well as 2-5 one sentence bullet points that summarise the paper. Please write the bullet points to summarise the key NEW findings. They should be designed to be complementary to the abstract - i.e. not repeat the same text. We encourage inclusion of key acronyms and quantitative information (maximum of 30 words / bullet point). Please use the passive voice. Please attach these in a separate file or send them by email, we will incorporate them accordingly.

You are also welcome to suggest a striking image or visual abstract to illustrate your article. If you do please provide a jpeg file 550 px-wide x 400-px high.

10) A Conflict of Interest statement should be provided in the main text

11) Please note that we now mandate that all corresponding authors list an ORCID digital identifier. This takes <90 seconds to complete. We encourage all authors to supply an ORCID identifier, which will be linked to their name for unambiguous name identification.

Currently, our records indicate that the ORCID for your account is 0000-0002-7725-8646.

Please click the link below to modify this ORCID:
Link Not Available

12) The system will prompt you to fill in your funding and payment information. This will allow Wiley to send you a quote for the article processing charge (APC) in case of acceptance. This quote takes into account any reduction or fee waivers that you may be eligible for. Authors do not need to pay any fees before their manuscript is accepted and transferred to our publisher.

*Additional important information regarding Figures

Each figure should be given in a separate file and should have the following resolution:
Graphs 800-1,200 DPI
Photos 400-800 DPI
Colour (only CMYK) 300-400 DPI"

Figures are not edited by the production team. All lettering should be the same size and style; figure panels should be indicated by capital letters (A, B, C etc). Gridlines are not allowed except for log plots. Figures should be numbered in the order of their appearance in the text with Arabic numerals. Each Figure must have a separate legend and a caption is needed for each panel.

*Additional important information regarding figures and illustrations can be found at https://bit.ly/EMBOPressFigurePreparationGuideline. See also figure legend preparation guidelines: https://www.embopress.org/page/journal/17574684/authorguide#figureformat

The system will prompt you to fill in your funding and payment information. This will allow Wiley to send you a quote for the article processing charge (APC) in case of acceptance. This quote takes into account any reduction or fee waivers that you may be eligible for. Authors do not need to pay any fees before their manuscript is accepted and transferred to our publisher.

***** Reviewer's comments *****

Referee #1 (Comments on Novelty/Model System for Author):

This is a massive amount of data, that have addressed to a large extent the concerns raised by this and other reviewers.
The potential for clinical translation is there, but only a clinical trial may reveal whether inhibiting CD38 may result in a benefit for the patient. A study in a large animal model would be informative but these studies have become economically unaffordable and it would be unfair to request this to the Authors.

Referee #1 (Remarks for Author):

The Authors carried out a massive amount of work to address all the Reviewers concerns. The manuscript is now much improved and I have no further comments.

Referee #2 (Remarks for Author):

The authors have addressed most of my previous concerns and the revised manuscript is significantly improved.

The authors performed the requested editorial changes.

We are pleased to inform you that your manuscript is accepted for publication and is now being sent to our publisher to be included in the next available issue of EMBO Molecular Medicine.

EMBO PRESS

YOU MUST COMPLETE ALL CELLS WITH A PINK BACKGROUND

PLEASE NOTE THAT THIS CHECKLIST WILL BE PUBLISHED ALONGSIDE YOUR PAPER

Corresponding Author Name:	
Journal Submitted to:	
Manuscript Number:	

Reporting Checklist For Life Sciences Articles (Rev. June 2017)

This checklist is used to ensure good reporting standards and to improve the reproducibility of published results. These guidelines are consistent with the Principles and Guidelines for Reporting Preclinical Research issued by the NIH in 2014. Please follow the journal's authorship guidelines in preparing your manuscript.

A- Figures

1. Data

The data shown in figures should satisfy the following conditions:

- the data were obtained and processed according to the field's best practice and are presented to reflect the results of the experiments in an accurate and unbiased manner.
- figure panels include only data points, measurements or observations that can be compared to each other in a scientifically meaningful way.
- graphs include clearly labeled error bars for independent experiments and sample sizes. Unless justified, error bars should not be shown for technical replicates.
- if n< 5, the individual data points from each experiment should be plotted and any statistical test employed should be justified
- Source Data should be included to report the data underlying graphs. Please follow the guidelines set out in the author ship guidelines on Data Presentation.

2. Captions

Each figure caption should contain the following information, for each panel where they are relevant:

- a specification of the experimental system investigated (eg cell line, species name).
- the assay(s) and method(s) used to carry out the reported observations and measurements
- an explicit mention of the biological and chemical entity(ies) that are being measured.
- an explicit mention of the biological and chemical entity(ies) that are altered/varied/perturbed in a controlled manner.

- the exact sample size (n) for each experimental group/condition, given as a number, not a range;
- a description of the sample collection allowing the reader to understand whether the samples represent technical or biological replicates (including how many animals, litters, cultures, etc.).
- a statement of how many times the experiment shown was independently replicated in the laboratory.
- definitions of statistical methods and measures:
  - common tests, such as t-test (please specify whether paired vs. unpaired), simple χ2 tests, Wilcoxon and Mann-Whitney tests, can be unambiguously identified by name only, but more complex techniques should be described in the methods section;
  - are tests one-sided or two-sided?
  - are there adjustments for multiple comparisons?
  - exact statistical test results, e.g., P values = x but not P values < x;
  - definition of 'center values' as median or average;
  - definition of error bars as s.d. or s.e.m.

Any descriptions too long for the figure legend should be included in the methods section and/or with the source data.

In the pink boxes below, please ensure that the answers to the following questions are reported in the manuscript itself. Every question should be answered. If the question is not relevant to your research, please write NA (non applicable). We encourage you to include a specific subsection in the methods section for statistics, reagents, animal models and human subjects.

USEFUL LINKS FOR COMPLETING THIS FORM

http://www.antibodypedia.com	Antibodypedia
http://1degreebio.org	1DegreeBio
http://www.equator-network.org/reporting-guidelines/improving-bioscience-research-reporting	ARRIVE Guidelines
http://grants.nih.gov/grants/olaw/olaw.htm	NIH Guidelines in animal use
http://www.mrc.ac.uk/Ourresearch/Ethicsresearchguidance/Useofanimals/index.htm	MRC Guidelines on animal use
http://ClinicalTrials.gov	Clinical Trial registration
http://www.consort-statement.org	CONSORT Flow Diagram
http://www.consort-statement.org/checklists/view/32-consort/66-title	CONSORT Check List
http://www.equator-network.org/reporting-guidelines/reporting-recommendations-for-tumour-	REMARK Reporting Guidelines (marker prognostic studies)
http://datadryad.org	Dryad
http://figshare.com	Figshare
http://www.ncbi.nlm.nih.gov/gap	dbGAP
http://www.ebi.ac.uk/ega	EGA
http://biomodels.net/	Biomodels Database
http://biomodels.net/miriam/	MIRIAM Guidelines
http://jjj.biochem.sun.ac.za	JWS Online
http://oba.od.nih.gov/biosecurity/biosecurity_documents.html	Biosecurity Documents from NIH
http://www.selectagents.gov/	List of Select Agents

B- Statistics and general methods

Please fill out these boxes (Do not worry if you cannot see all your text once you press return)

1.a. How was the sample size chosen to ensure adequate power to detect a pre-specified effect size?	No statistical methods were used to pre-determine sample sizes but our sample sizes are similar to those reported in our previous publications. In our experimental design, α is controlled and set low, at α = 0.05, to maintain a high specificity (1 – α), which is the chance of a true negative. PAGE 17
1.b. For animal studies, include a statement about sample size estimate even if no statistical methods were used.	The number necessary for each animal group and for each experiment were chosen in order to obtain sufficient power to ensure the statistical validity of the results of the study (with a α= 0,05) usually around 10, while respecting animal welfare rules by limiting the total number of animals used in the study. PAGE 17
2. Describe inclusion/exclusion criteria if samples or animals were excluded from the analysis. Were the criteria pre-established?	An outlier analysis was performed using the ROUT method (Q=1%) on each quantitative data set. Some data has been removed prior to their analysis due to animal death or removal in accordance with the animal welfare guidelines for reducing suffering of the animals during the experimental procedure, missed tissue/blood collection, insufficient sample volumes or poor histological quality. PAGE 17
3. Were any steps taken to minimize the effects of subjective bias when allocating animals/samples to treatment (e.g. randomization procedure)? If yes, please describe.	The animals of the different experimental groups are matched according to age for each protocol. The mice contained in a home cage were randomly assigned to an experimental group. PAGE 11
For animal studies, include a statement about randomization even if no randomization was used.	The mice contained in a home cage were randomly assigned to an experimental group and age-matched. PAGE 11
4.a. Were any steps taken to minimize the effects of subjective bias during group allocation or/and when assessing results (e.g. blinding of the investigator)? If yes please describe.	The following experiments were performed in blind : Fig 1 A-J ; Fig 2 A,B,F; Fig 3 B,C ; Fig 4 A,B; Fig 5 A,D,E; Fig 6 B,D,E,F; Fig S1 A1,A2,C1,C2,D1,D2; Fig S2 A,B,C; Fig S3 A,B,F; Fig S4 B,C; Fig S5 A,B; Fig S6 B,C; Fig S7 B,C; Fig S8
4.b. For animal studies, include a statement about blinding even if no blinding was done	Blind experiments are described in the relevant method section(PAGE 11 and following).
5. For every figure, are statistical tests justified as appropriate?	All the statistics were performed with GraphPad Prism 8. An outlier analysis was performed using the ROUT method (Q=1%) on each quantitative data set.<br>For all groups, normality was tested with Shapiro-Wilk or D'Agostino-Pearson tests. For the comparison between two groups , the variance difference was assessed with a F-test. If all the groups have a normal distribution and equal variances, a two-tailed Student's t-test was performed. Otherwise, if the groups have a normal distribution with a difference in variance, a two-tailed Welch's t-test was performed. If at least one of group has a non-normal distribution, or with n ≤ 3, a two-tailed Mann-Whitney test was performed. For a comparison between at least 3 groups, the variance difference was assessed with a Brown-F orsythe test. If all the groups have a normal distribution and no variance difference, a one-way ANOVA test was performed. If statistically significant, the one-way ANOVA was followed by a Fisher LSD's multiple comparisons test, or by Student's t-tests when appropriate. If all the groups have a normal distribution and a variance difference, a one-way Welch's ANOVA test was used and when significant, it was followed by a Welch's t-test for multiple comparisons. Otherwise, if at least one of the groups has a non-normal distribution, a Kruskal-Wallis test was performed, and if significant, the test was followed by an uncorrected Dunn's test for multiple comparisons, or by Mann-Whitney tests. For survival rates comparison, a log-rank (Mantel-Cox), log-rank for trend and Gehan-Breslow-Wilcoxon tests<br><br>were performed. For the inhibitor (78c) effect and repeated measures of a chronic treadmill exercise, a two-way ANOVA test was performed. For contingency tables comparison, a Chi-square test was performed. The cumulative distribution comparisons were analyzed with a Kolmogorov–Smirnov distribution test. PAGE 17

Do the data meet the assumptions of the tests (e.g., normal distribution)? Describe any methods used to assess it.	For all the groups, normality was tested with Shapiro-Wilk or D'Agostino-Pearson tests. For a comparison between 2 groups, the variance difference was assessed with an F-test. For a comparison between at least 3 groups, the variance difference was assessed with a Brown-Forsythe test. Appropriate tests were then used for mean/median comparisons. PAGE 17
Is there an estimate of variation within each group of data?	For a comparison between groups, the variation within each group is assessed with a Brown-Forsythe test. PAGE 17
Is the variance similar between the groups that are being statistically compared?	If groups with different variances were compared,statistical tests were chosen to account for this. For a comparison between two groups, if the variance was different between them, a Welch's t-test was used instead of a Student's t-test. For a comparison between at least 3 groups, if the variance was different between them, a Welch's ANOVA test was used instead of a regular ANOVA test, and the post-hoc test used for multiple comparison were Welch's t-test instead of Fisher's LSD test. PAGE 17

C- Reagents

6. To show that antibodies were profiled for use in the system under study (assay and species), provide a citation, catalog number and/or clone number, supplementary information or reference to an antibody validation profile. e.g., Antibodypedia (see link list at top right), 1DegreeBio (see link list at top right).	All the antibodies and primer references are detailed in the method section (PAGE 11 and following). All the items were purchased and the providers are listed in the method section.
7. Identify the source of cell lines and report if they were recently authenticated (e.g., by STR profiling) and tested for mycoplasma contamination.	Skeletal human cell line provided by AFM bank of tissue for research . In details, myoblasts were purified with MACS and the final cell lines (DMD 6311 and healthy CHQ), were provided by Drs. Vincent Mouly (Hôpital Pitié-Salpêtrière, Paris, France) and Francesco Muntoni (Centre for Neuromuscular Disease, London, UK). The cell lines were tested for contaminations by the providers. PAGE 15

* for all hyperlinks, please see the table at the top right of the document

D- Animal Models

8. Report species, strain, gender, age of animals and genetic modification status where applicable. Please detail housing and husbandry conditions and the source of animals.	The colony was set up by crossing mdx mice with CD38-/- mice to generate mdx/CD38-/- mice. Mdx mice (C57BL/10ScSn-Dmdmdx/J) were purchased at the Jackson laboratory. CD38-/- mice with a deletion of exon 2 and 3 in the CD38 gene were obtained from the Lund and Randall Laboratory [University of Alabama–Birmingham (UAB), AL, USA]. The CD38-/- mice have been backcrossed 10 times onto the C57BL/6 inbred strain background and were shown to exhibit no residual enzymatic activity in vitro (Partida-Sánchez et al, 2001). The animals are by 3-4 mice/cage and have enviromental enrichement, access to food and water ad libitum. PAGE 11
9. For experiments involving live vertebrates, include a statement of compliance with ethical regulations and identify the committee(s) approving the experiments.	The experimental procedures were designed to eliminate or limit any physical suffering of the mice. Mice will be monitored for possible pain, distress or discomfort. This follow-up includes an evaluation of the weight loss, physical appearance and behavior of the animal. Animal care and experimental procedures complied with the European Communities Council Directive (CEE 86/609/EEC), EU Directive 2010/63/EU, and local ethic committee (Paris Centre et Sud, N°59). The protocols were approved by the French Departmental Direction of Animal Protection (2015-11 #1027). For experiments with 78c the protocol was IACUC (Institutional animal care and use committee) approved. PAGE 11
10. We recommend consulting the ARRIVE guidelines (see link list at top right) (PLoS Biol. 8(6), e1000412, 2010) to ensure that other relevant aspects of animal studies are adequately reported. See author guidelines, under 'Reporting Guidelines'. See also: NIH (see link list at top right) and MRC (see link list at top right) recommendations. Please confirm compliance.	Our study complies with the french animal welfare guidelines. PAGE 11

E- Human Subjects

11. Identify the committee(s) approving the study protocol.	NA
12. Include a statement confirming that informed consent was obtained from all subjects and that the experiments conformed to the principles set out in the WMA Declaration of Helsinki and the Department of Health and Human Services Belmont Report.	NA
13. For publication of patient photos, include a statement confirming that consent to publish was obtained.	NA
14. Report any restrictions on the availability (and/or on the use) of human data or samples.	NA
15. Report the clinical trial registration number (at ClinicalTrials.gov or equivalent), where applicable.	NA
16. For phase II and III randomized controlled trials, please refer to the CONSORT flow diagram (see link list at top right) and submit the CONSORT checklist (see link list at top right) with your submission. See author guidelines, under 'Reporting Guidelines'. Please confirm you have submitted this list.	NA
17. For tumor marker prognostic studies, we recommend that you follow the REMARK reporting guidelines (see link list at top right). See author guidelines, under 'Reporting Guidelines'. Please confirm you have followed these guidelines.	NA

F- Data Accessibility

18: Provide a "Data Availability" section at the end of the Materials & Methods, listing the accession codes for data generated in this study and deposited in a public database (e.g. RNA-Seq data: Gene Expression Omnibus GSE39462, Proteomics data: PRIDE PXD000208 etc.) Please refer to our author guidelines for 'Data Deposition'.<br><br>Data deposition in a public repository is mandatory for:<br>a. Protein, DNA and RNA sequences<br>b. Macromolecular structures<br>c. Crystallographic data for small molecules<br>d. Functional genomics data<br>e. Proteomics and molecular interactions	This study includes no data deposited in external repositories.
19. Deposition is strongly recommended for any datasets that are central and integral to the study; please consider the journal's data policy. If no structured public repository exists for a given data type, we encourage the provision of datasets in the manuscript as a Supplementary Document (see author guidelines under 'Expanded View' or in unstructured repositories such as Dryad (see link list at top right) or Figshare (see link list at top right).	**A supplementary Excel file with the datasets (Figures 1 to 6) is provided**
20. Access to human clinical and genomic datasets should be provided with as few restrictions as possible while respecting ethical obligations to the patients and relevant medical and legal issues. If practically possible and compatible with the individual consent agreement used in the study, such data should be deposited in one of the major public access-controlled repositories such as dbGAP (see link list at top right) or EGA (see link list at top right).	NA
21. Computational models that are central and integral to a study should be shared without restrictions and provided in a machine-readable form. The relevant accession numbers or links should be provided. When possible, standardized format (SBML, CellML) should be used instead of scripts (e.g. MATLAB). Authors are strongly encouraged to follow the MIRIAM guidelines (see link list at top right) and deposit their model in a public database such as Biomodels (see link list at top right) or JWS Online (see link list at top right). If computer source code is provided with the paper, it should be deposited in a public repository or included in supplementary information.	NA

G- Dual use research of concern

22. Could your study fall under dual use research restrictions? Please check biosecurity documents (see link list at top right) and list of select agents and toxins (APHIS/CDC) (see link list at top right). According to our biosecurity guidelines, provide a statement only if it could.	NA
